# Genetic diversity for starch quality and alkali spreading value in sorghum

**DOI:** 10.1002/tpg2.20067

**Published:** 2020-12-01

**Authors:** Stefanie Griebel, Adeyanju Adedayo, Mitchell R. Tuinstra

**Affiliations:** ^1^ Department of Agronomy Purdue University West Lafayette IN 47907 USA; ^2^ Department of Crop Sciences, Division of Plant Breeding Methodology University of Göttingen Göttingen 37075 Germany

## Abstract

Sorghum is an important food crop in many parts of Africa and Asia. Landraces of sorghum are known to exhibit variation in food quality traits including starch and protein content and composition. In this study, a panel of diverse sorghum breeding lines and 788 sorghum conversion (SC) lines representing the global germplasm diversity of the crop were evaluated for variation in starch quality based on alkali spreading value (ASV). A small number of genotypes with stable expression of the ASV+ phenotype across seasons were identified; mostly representing *Nandyal* types from India. Genetic studies showed the ASV+ phenotype was inherited as a recessive trait. Whole genome resequencing of ASV+ donor lines revealed SNPs in genes involved in starch biosynthesis. A genome wide association study (GWAS) identified a significant SNP associated with ASV near *Sobic.010G273800*, a starch branching enzyme I precursor, and *Sobic.010G274800* and *Sobic.010G275001*, both annotated as glucosyltransferases. Physiochemical analyses of accessions with contrasting ASV phenotypes demonstrated an environment dependent lower starch gelatinization temperature (GT), amylose content of approximately 22%, and good gel consistency. The starch quality attributes of these lines could be valuable in food products that require good gel consistency and viscosity.

## Core Ideas

- Diverse sorghum accessions exhibit variation in alkali spreading value (ASV)
- A cluster of *Nandyal* type *durra* sorghums from India exhibit the ASV+ phenotype
- Testcrosses demonstrated recessive inheritance for the ASV+ phenotype
- GWAS identified significant SNPs and candidate genes for ASV

**Abbreviations**
ASV: Alkali spreading value
FDR: false discovery rate
GAPIT: Genome Association and Prediction Integrated Tool
GBS: genotyping by sequencing
GT: gelatinization temperature
GWAS: genome wide association study
LD: linkage disequilibrium
LMM: logistic mixed model
MAF: minor allele frequency
MLM: mixed linear model
PC: principal component
PCA: principal component analysis
SAP: Sorghum Association Panel
SBE: Starch Branching Enzyme
SC: sorghum conversion
SDP: Sorghum Diversity Panel
SNP: single nucleotide polymorphism
SS: Starch Synthase
TASSEL: Trait Analysis by aSSociation, Evolution and Linkage
T_c_
: completion starch GT
T_o_
: onset starch GT
T_p_
: peak starch GT
WL: West Lafayette.

## 1 INTRODUCTION

Sorghum [*Sorghum bicolor* (L.) Moench] serves as a staple food crop for millions of people in Africa and Asia (FAO, 2016; House, Gomez, Sun, Murty, & Verma, 2000; Kimber, 2000). It is commercially important in many regions of the world but especially important in low‐income and food‐deficient countries (FAO, 2019). The utilization of sorghum is regionally and culturally dependent and of great economic value for smallholder farmers because it is less expensive than other imported cereals (House et al., 2000; Taylor & Dewar, 2000).

*Sorghum bicolor* is one of three sorghum species and has three subspecies (Dahlberg, 2000). The cultivated sorghums belong to the subspecies *Sorghum bicolor* subsp. *bicolor* and are classified into five races based on plant/panicle/seed characteristics (Dahlberg, 2000; Harlan & de Wet, 1972). The five basic cultivated races are bicolor, guinea, kafir, caudatum, and durra that combine to form intermediate races (Dahlberg, 2000; Harlan & de Wet, 1972). Phenotypic evaluations and genomic studies reported that the five sorghum races originated in Africa and Asia (Billot et al., 2013; Brenton et al., 2016; Dahlberg, 2000; Deu, Rattunde, & Chantereau, 2006; Harlan & de Wet, 1972; Kimber, Dahlberg, & Kresovich, 2013; Morris et al., 2013; Sukumaran et al., 2012); with northeast Africa as the center of diversity (Paterson et al., 2009).

The sorghum germplasm collections represent tremendous genetic diversity with more than 36,000 accessions available and stored at ICRISAT in India (Billot et al., 2013; Kimber et al., 2013) and more than 40,000 accessions at the USDA National Center for Genetic Resources Preservation (USDA‐ARS‐PGRCU) (Kimber et al., 2013). Most of the exotic sorghum accessions stored at USDA‐ARS‐PGRCU are photoperiod sensitive so the Sorghum Conversion Program was initiated by Stephens, Miller, and Rosenow (1967) to make the collection more readily available for research and breeding in the temperate zone. The program converted alien sorghums from the world collection into photoperiod insensitive, short‐statured, early‐maturing sorghums (Rosenow et al., 1997a, 1997b; Stephens et al., 1967). Over 800 sorghum conversion lines (SC lines) are currently available (Hayes et al., 2015; Kimber et al., 2013). The collection of SC lines was developed to represent the genetic diversity of the crop and provides the basis for sorghum diversity panels such as the Sorghum Association Panel (SAP) (Adeyanju, Little, Yu, & Tesso, 2015; Boyles et al., 2017; Casa et al., 2008; Cuevas, Rosa‐Valentin, Hayes, Rooney, & Hoffmann, 2017; Mace et al., 2013; Morris et al., 2013; Shenstone et al., 2018; Sukumaran et al., 2012) and the Sorghum Diversity Panel (SDP) (Hayes et al., 2015).

The sorghum diversity panels are important resources used in genome wide association studies (GWAS). Several different panels have been genotyped using genotype‐by‐sequencing (GBS) strategies and these markers are associated with phenotypes to identify quantitative trait loci (QTLs) of interest (Adeyanju et al., 2015; Boyles et al., 2017; Morris et al., 2013). The sorghum linkage disequilibrium (LD) decay varies among collections and is reported to be approximately 15 kb (Kimber et al., 2013) with slower LD decay in inbred lines (19.7 kb) than in landraces (10.3 kb) (Mace et al., 2013) and can be as fast as 1 kb (Morris et al., 2013). Mixed linear models are commonly used for GWAS (Adeyanju et al., 2015; Huang et al., 2010; Morris et al., 2013; Zhao et al., 2011). Recently, the Logistic Mixed Model (LMM) was proposed for analysis of binary traits in maize and sorghum diversity panels (Shenstone et al., 2018).

The alkali spreading phenotype (ASV) has been reported to identify sorghum genotypes with contrasting starch thermal properties (Griebel et al., 2019a). The key gene for ASV in rice is the *ALK* (*SSII‐3*) gene that also controls starch gelatinization temperature (GT) (Gao et al., 2011; Tian et al., 2009). GWAS for ASV has been conducted in rice with several genomic regions associated with the trait (Huang et al., 2010; Huang et al., 2012; Mogga, Sibiya, Shimelis, Lamo, & Yao, 2018; Song, Arif, Zhang, Sze, & Zhang, 2019; Zhao et al., 2011). Only a few of the associations were close to candidate genes such as one on chromosome 7 that was close to amylase inhibitors (Huang et al., 2012), chromosome 6 near the *ALK (SSII‐3)* gene (Huang et al., 2010; Zhao et al., 2011), chromosome 8 within 200 kb of the *SSII‐2* (Zhao et al., 2011), and chromosome 5 (Song et al., 2019). The ASV hits were not confirmed across years (Zhao et al., 2011). Griebel et al. (2019b) showed that *SSIIa* and *SBEIIb* are key genes controlling the alkali spreading phenotype in EMS mutant populations of sorghum. These genes have contrasting effects on starch GT, amylose content, and paste viscosity profiles (Griebel et al., 2019b).

**Core Ideas**

- Diverse sorghum accessions exhibit variation in alkali spreading value (ASV)
- A cluster of *Nandyal* type *durra* sorghums from India exhibit the ASV+ phenotype
- Testcrosses demonstrated recessive inheritance for the ASV+ phenotype
- GWAS identified significant SNPs and candidate genes for ASV

At present, nothing is known about the expression, inheritance, or genetic architecture for ASV in the standing variation of sorghum. Thus, we aim to (1) evaluate a diverse collection of more than 800 breeding and SC‐lines of sorghum for ASV, (2) conduct a GWAS to determine the genetic architecture and candidate genes for ASV, (3) characterize the starch phenotypes of accessions with ASV+ phenotypes, and (4) determine the mode of inheritance for the ASV+ trait.

## 2 MATERIALS AND METHODS

### 2.1 Plant material

A sorghum diversity panel of 840 lines (SC lines and breeding lines) was grown in West Lafayette in 2013 and 2017. Plants were self‐pollinated each year and seeds for each line were used for subsequent analyses. SC489, SC491, SC587 and SC589 were found to exhibit a stable ASV+ phenotype and seeds of these accessions were produced in West Lafayette 2017 and 2018. Three biological replicates per line were evaluated in 2017 and four biological replicates were evaluated in 2018. Tx623, Sepon82 and Macia were used as controls in both seasons with two biological replicates each year.

### 2.2 Alkali spreading test

The alkali test was conducted as described in Griebel et al. (2019a). Preliminary testing of ASV at 1.5%, 1.8%, and 2.0% KOH was used to identify the optimal conditions for testing the genotypes in the diversity panel. Sorghum seeds were cut longitudinally and each half was placed into a well of a 96 well plate. The wells were filled with 1.8% KOH and treated for 24 h. Each genotype was evaluated with two seeds in West Lafayette 2013 (preliminary study) and eight seeds in West Lafayette 2017. A positive ASV phenotype was scored as 1 (complete gelatinization) and negative ASV, similar to Tx623, Sepon82, and Macia, as 0 (no swelling, no disintegration). SC489, SC491, SC587 and SC589 and their crosses from genetic studies were screened for ASV using 32 seeds each.

### 2.3 Mode of inheritance for ASV

SC489, SC491, SC587 and SC589 were crossed to ATx623 in West Lafayette 2018 to produce F_1_ hybrid seeds. The F_1_ seeds were screened for ASV using 32 seeds each.

### 2.4 CTAB DNA extraction

DNA was extracted from individual plants using a CTAB extraction protocol (Griebel et al., 2019b; Murray & Thompson, 1980). In summary, tissues samples were ground in 800 μL Extraction Buffer (1.2 M NaCl, 100 mM Tris pH 8, 20 mM EDTA pH8, 2% CTAB, 0.1% BME) in a 2 ml Eppendorf tube. The samples were heated to 60 °C for 45 min followed by extraction with 800 μl chloroform (24 chloroform:1 isoamyl alcohol). The aqueous faction was combined with 1070 μl Dilution Buffer (100 mM Tris pH 8, 20 mM EDTA pH 8, 2% CTAB) and incubated at 60 °C for 30 min. The samples were centrifuged and the pellets were washed with 70% ethanol and dissolved in high‐salt TE with RNaseA (1 M NaCl, 10 mM Tris pH 8, 2 mM EDTA pH 8, RNaseA 50 μg/m). After RNaseA treatment, the DNA was precipitated with pure ethanol and re‐suspended in TE Buffer (10 mM Tris pH 8, 1 mM EDTA pH 8).

### 2.5 Next generation sequencing (NGS) and analyses

Next generation sequencing analyses including reads filtering, contaminants cleaning, mapping to the reference genome, and SNP calling using GATK were conducted as described in Griebel et al. (2019b).

### 2.6 Physiochemical analyses

Flour preparation, starch extraction and starch thermal properties using differential scanning calorimetry (DSC) were analysed as described in Griebel et al. (2019a). The samples were milled into a fine flour using a ball mill. Seeds were milled in 30s intervals to avoid starch damage. Starch extraction protocol is explained in detail in Griebel et al. (2019a) and Benmoussa and Hamaker (2011). In short, flour samples were treated with NaCl and sodium metabisulfite followed by a sonication step. The samples were than filtered through a standard 230 mesh‐screen and sonicated again. After centrifugation, a sucrose density solution (65%) was used to obtain starch pellets. Starch thermal properties were conducted in duplicate for each starch sample using differential scanning calorimetry (DSC, Q2000‐1223, TA instruments, New Castle, DE, USA) (Griebel et al., 2019a). Tzero hermetic aluminium pans and lids were used containing a starch:deionized water ratio of 1:3 and samples were stored at 4 °C overnight before being analysed. The heating profile was chosen from 20 °C to 120 °C at a rate of 5 °C/min. TA instruments universal analysis software was used for data analysis.

Moisture determination, viscosity analyses, and amylose and amylopectin analyses were determined following the procedures described in Griebel et al. (2019b). Viscosity analysis required adjustment for moisture content using a standard oven method (Bradley Jr., 2014; Griebel et al., 2019b). The Rapid Visco Analyzer (RTE‐100, model 4, Newport Scientific, Australia) was used to run paste viscosity analysis in the setting of standard method 1. The samples were prepared as water‐flour dispersions of 11.86% (Bouvier & Campanella, 2014). Amylose and amylopectin analysis was conducted with the K‐AMYL Kit from Megazyme.

### 2.7 Proximate analysis NIRS

NIRS was used to predict protein (db), moisture and starch (db) of whole sorghum seeds from samples produced in Puerto Rico 2016/2017 and West Lafayette 2017 using a Perten NIRS (Model DA 7250, Serial Number 1211581). Two technical replications were conducted for each sample.

### 2.8 Statistical analysis for starch quality traits

Linear mixed model spreading analysis of variance (ANOVA) with the Tukey‐Kramer multiple comparison adjustment was performed in SAS 9.4 using the GLIMMIX procedure to compare starch traits across genotypes and environments as described in Griebel et al. (2019b). Parts of the data analysis for this paper were generated using SAS software, Version 9.4 of the SAS System for Windows (SAS Institute Inc., Cary, NC, USA).

### 2.9 Principal component analysis

The race designations and country of origin for the Sorghum Conversion lines were kindly provided by R.R. Klein (personal communication, 2019) with additional information summarized from the USDA GRIN database (USDA‐ARS, 2019). The countries of origin were grouped by continent for the minority of lines from Asia and the Americas and kept a single group for India. The majority of the lines are from the African continent and those countries were grouped into regions based on United Nation classifications (United Nations, 2019). The non‐SC lines were treated as breeding lines and were not given a race or country designation. Principal component analysis was conducted using GAPIT (based on prcomp) (R. Lipka, personal communication, 2019) with 5 PCs. The PCs were plotted using R v. 3.5.1 and the package “ggfortify” (Horikoshi & Tang, 2016; R core Team, 2013; Tang, Horikoshi, & Li, 2016).

### 2.10 Genome wide association analysis

The imputed genotype‐by‐sequencing data were kindly provided by Dr. Patrick Brown and raw data are available at University of Illinois database, https://doi.org/10.13012/B2IDB-7570206_V1 (P. Brown, personal communication, 2019; Brown, 2017). The GBS data were aligned to the sorghum reference genome version 3 from Phytozome11 (Goodstein et al., 2012, version 11) and imputed using the Beagle 4 software (P. Brown, personal communication, 2019). 80,103 SNP markers at a minor allele frequency (MAF) of 0.025 were selected from the total set of markers and used in the genome wide association study.

The ASV data were prepared as binary data with 0 = ASV‐ and 1 = ASV+. ASV− were only lines that had no swelling and disintegration at all in any of the seeds tested. Trait correlations were calculated using SAS 9.4 and the proc corr procedure. The ASV data from 2013 and 2017 were used as input phenotypes for the GWAS using a mixed linear model (MLM) (Lipka et al., 2012) with 80,103 SNP markers. The Genome Association and Prediction Integrated Tool (GAPIT) (Lipka et al., 2012; Zhang et al., 2010) was used for the mixed linear model (MLM). The PCs were calculated using GAPIT and set to 5. The MLM in GAPIT was run with the default settings and the Van Raden kinship algorithm. The significant SNPs were determined using a bonferroni (Bonferroni, 1936) threshold of 6.242E‐07 and at an FDR of adjusted p‐values at 0.05 (Benjamini & Hochberg, 1995). Manhattan plots were created using the GWAS results and the R package “qqman” (R Core Team, 2013; Turner, 2014).

### 2.11 Neighbour‐joining tree

The neighbor joining tree was created using TASSEL 5.2.51 (Bradbury et al., 2007).

## 3 RESULTS

### 3.1 Race and geographic classification of the sorghum diversity panel

The sorghum diversity panel consisted of 788 SC lines from the sorghum conversion program and 51 sorghum breeding lines included as checks. The SC lines originated from diverse countries before being converted in the sorghum conversion program (Supplemental Table S1). The countries of origin are predominantly India (171 SC lines), Ethiopia (139 SC lines), Sudan (116 SC lines) and 305 SC lines from 25 other African countries (Supplemental Table S1). The races represented in the diversity panel are predominantly bicolor (37), caudatum (268), durra (171), durra‐bicolor (61), guinea (124), kafir (21) and kafir‐caudatum (22) (Supplemental Table S1).

**FIGURE 1:**
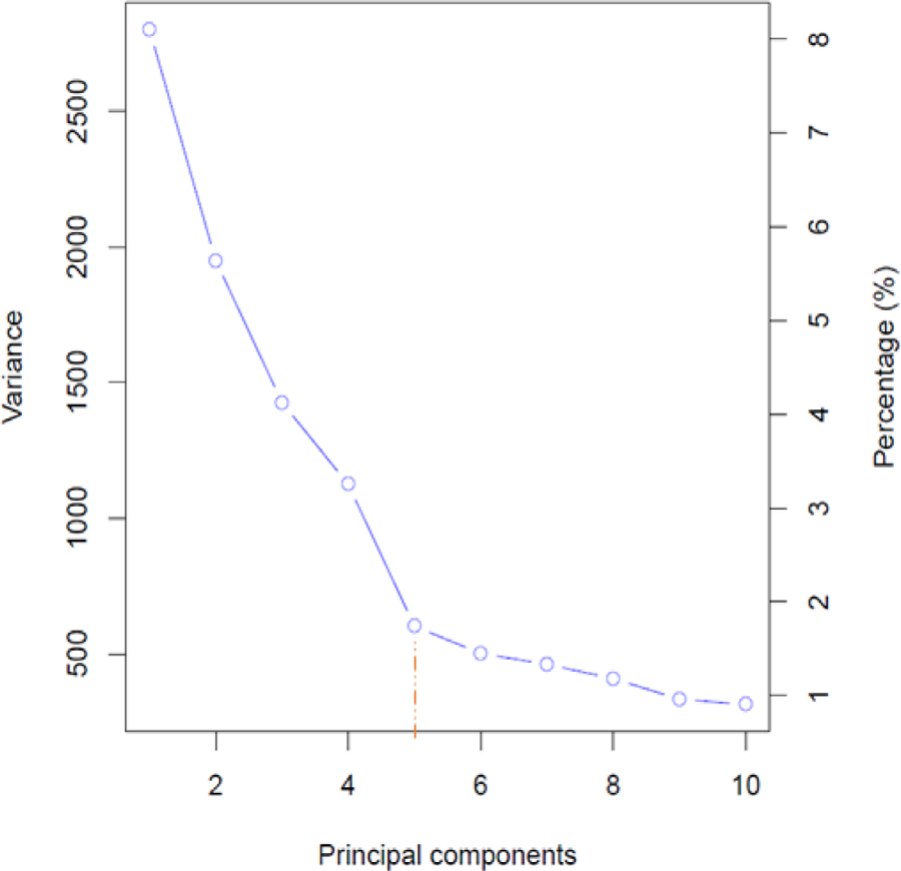
Scree plot of principal components (x‐axis) and their contribution to the variance determined from GBS data. Threshold (red line) at the characteristic elbow was used to estimate the optimal number of PCs

The GBS data (P. Brown, personal communication, 2019; Brown, 2017) were used to conduct principal component analysis to determine the covariates used for GWAS and to understand how the sorghum lines in the diversity panel clustered based on ASV, race, and origin. A scree plot from the principal component (PC) analysis showed that the first five PCs explained much of the variation (approximately 23%) from the SNP markers (Figure 1). The red line was drawn at the characteristic elbow as suggested by Wang et al. (2013).

**FIGURE 2:**
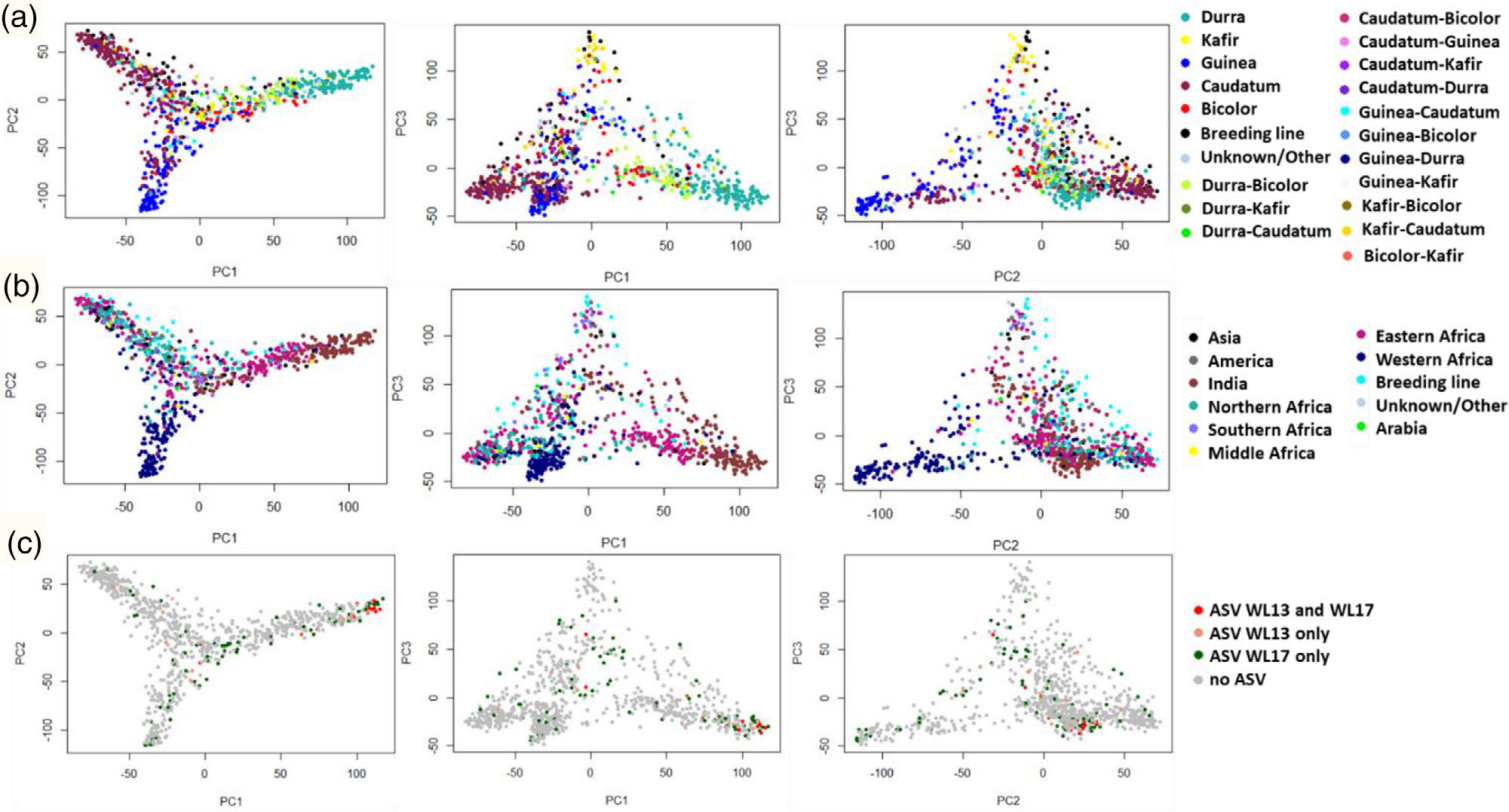
Principal components showing clustering of the sorghum diversity panel by (a) race, (b) geographic origin, and (c) ASV phenotype in 2D plots with principal components 1, 2 and 3. Genotypes were evaluated in West Lafayette in 2013 (WL13) and 2017 (WL17)

The first 3 PCs were plotted against each other and colored by their race classification, region of origin and ASV phenotype (Figure 2; Supplemental Table S1). PCs 4 and 5 explained the least variation and were not plotted in these comparisons. The PC1 captured most of the variation and differentiated the durra types from India and some durra‐bicolors from eastern Africa (Figure 2). The PC2 differentiated caudatum types originating from northern and eastern Africa as well as guineas and some caudatums from West Africa. The PC3 differentiated kafirs from eastern and southern Africa. The PC4 was dominated by the durra‐bicolor cluster and some bicolors from eastern Africa (not shown). PC5 did not show a clear distinction between groups but differentiated a group of guineas from West Africa listed as a second group in PC2 (not shown).

### 3.2 Variation in the ASV phenotype in the sorghum diversity panel

**FIGURE 3:**
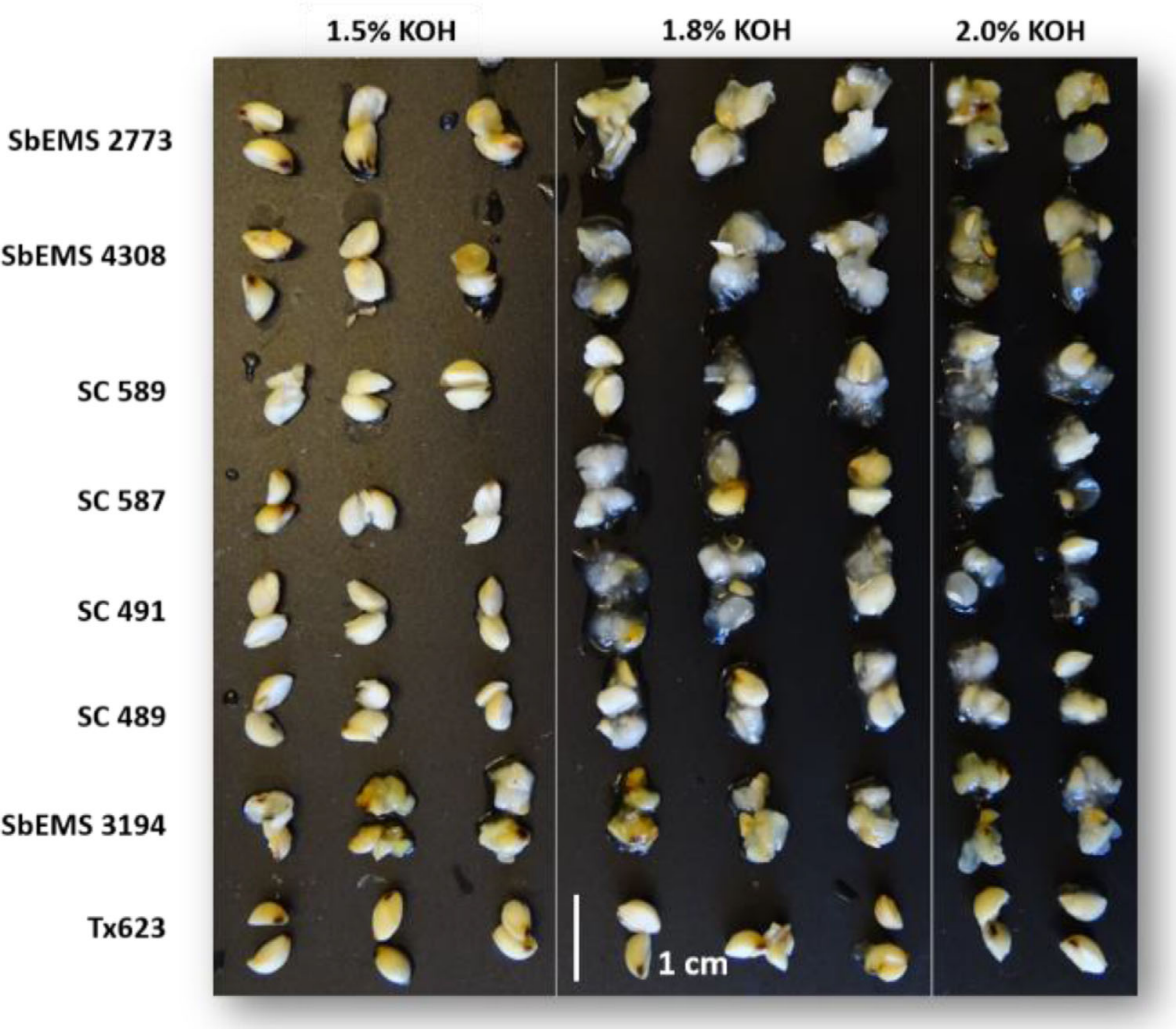
ASV phenotypes at 1.5%, 1.8% and 2.0% KOH for known SbEMS mutants and four SC lines having an ASV+ phenotype and Tx623 having an ASV‐ phenotype

The ASV assay described by Griebel et al. (2019a) was effective in distinguishing variation in starch quality in the standing variation of sorghum (Figure 3). Comparing concentrations of 1.5%, 1.8% and 2.0% KOH showed that Tx623 did not exhibit any ASV phenotype. Three SbEMS mutants with known ASV+ phenotype (Griebel et al., 2019a) and four SC lines clearly showed the expected ASV+ phenotype at 1.8% and 2.0%, while 1.5% KOH was not effective in differentiating the ASV phenotype.

Variation in the ASV phenotype was found in the sorghum diversity panel in 2013 and 2017 but the observed trait correlations between years was low (r = 0.23). The study in 2013 was set up as an exploratory study with 2 samples per genotype to determine whether variation for ASV could be detected among lines. There were 24 lines that exhibited the ASV phenotype in 2013 (salmon dots in Figure 2c; Supplemental Table S1). Subsequent studies showed that the expression of ASV was more variable in diverse sorghum accessions than in genetic mutants (Griebel et al., 2019b) so the study of ASV was repeated in 2017 with 8 seeds per genotype. Ninety‐five lines showed ASV in 2017 (green dots in Figure 2c; Supplemental Table S1) with only 13 lines exhibiting the phenotype in both years. The principal component analysis helped to show that these 13 lines (red dots in Figure 2c) clustered together at the tip of PC1 and were closely‐related *durra* sorghums from India (Figure 2). The genotypes exhibiting an ASV+ only in 2013 or only in 2017 demonstrated no clear race or geographic characteristics (Figure 2c).

**FIGURE 4:**
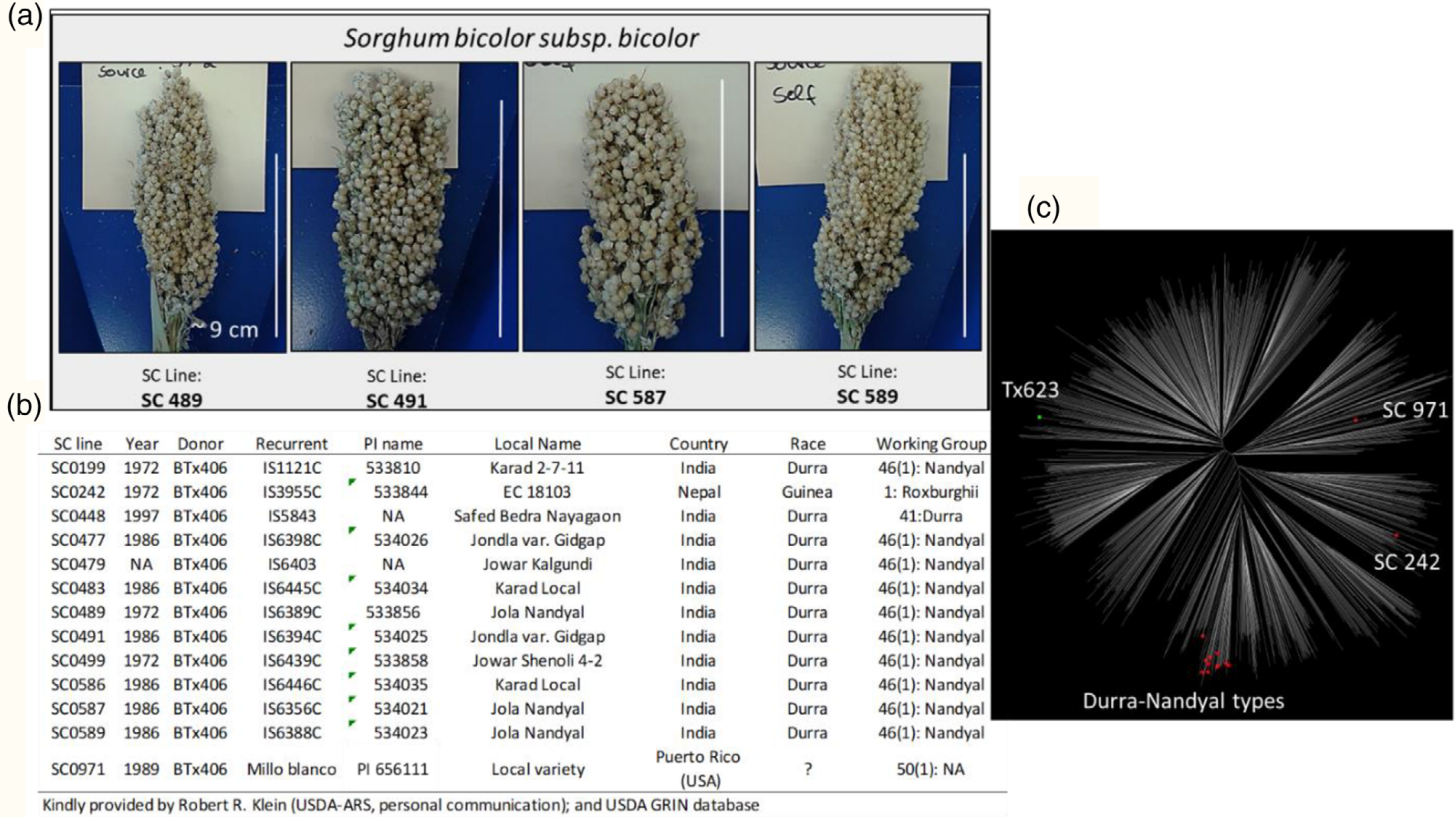
Origin and genetic relationships among sorghum lines exhibiting ASV phenotypes – (a) Panicles from a subset of lines exhibiting ASV+ phenotypes – SC489, SC491, SC587 and SC589. (b) SC lines exhibiting an ASV+ phenotype in West Lafayette 2013 and 2017. (c) A neighbor joining tree depicting genetic relationships among accessions with ASV+ phenotypes

The passport data of accessions exhibiting stable ASV+ phenotypes over years showed that 11 of the 13 lines were *durra* types representing the working group 46(1) Nandyal (Figure 4a, b, and c). The other two accessions were a *guinea* sorghum representing working group 1: Roxburghii and another accession that had no clear race classification (R. Klein, personal communication, 2019). A neighbor‐joining tree created in TASSEL (Bradbury et al., 2007) was used to depict the relationships among lines and showed that the Nandyal accessions clustered together and were closely related (Figure 4c). We selected four SC‐lines, SC489, SC491, SC587 and SC589 for further genetic, genomic and physiochemical analysis.

### 3.3 ASV+ sorghum accessions share SNPs in starch branching enzymes

Whole genome re‐sequencing of SC489, SC491, SC587 and SC589 revealed more than 200 common homozygous SNPs in candidate genes for starch biosynthesis. However, only 107 SNPs were shared in all four lines and 59 of these SNPs were in genes for starch branching enzymes, like SBEIIb *Sobic.004G163700* (Table 1).

**TABLE 1:** Whole genome re‐sequencing analyses show SC487, SC491, SC587, and SC589 share common SNPs in starch biosynthesis genes

Gene	Gene description	SNP, INDELS position (REF → ALT)
Sobic.001G083900	similar to Alpha 1,4‐glucan phosphorylase L isozyme	6491557 (G → C), 6493277 (A → C), 6496774 (T → C)
Sobic.001G239500	similar to putative uncharacterized protein SSI	24609069 (A → G), 24609559 (C → T), 24610385 (C → T), 24610644 (G → A), 24610664 (G → A), 24612553 (A → G), 24612704 (G → A), 24612729 (T → A), 24613491 (T → C), 24614286 (TA → T)
Sobic.002G116000	similar to GBSS IIa	14336638 (T → A), 14336679 (C → T), 14338839 (CT → C), 14340488 (A → G), 14340846 (G → A)
Sobic.002G233600	similar to Isoamylase‐type starch debranching enzyme ISO3	62457714 (C → T), 62458735 (T → G), 62461676 (C → A), 62461956 (C → T), 62462103 (G → A), 62462895 (T → C), 62462994 (T → C), 62463176 (G → A), 62466487 (A → T), 62467260 (G → A), 62467479 (T → C), 62468169 (T → A)
Sobic.003G213800	similar to Putative 1,4‐alpha‐glucan branching enzyme	54790394 (A → G), 54790433 (C → T), 54790535 (T → C), 54790620 (G → A), 54790751 (G → T), 54791826 (T → G), 54791826 (T → G), 54792264 (G → A), 54792265 (A → G), 54793301 (A → AAGTGAAATTATG), 54793305 (T → A)
Sobic.004G163700	ae1, SbeIIb, similar to 1,4‐alpha‐glucan‐branching enzyme 2	51292259 (A → AAC), 51292274 (C → CT), 51292275 (C → A), 51292342 (A → G), 51292345 (G → C), 51292352 (G → A), 51292365 (T → C), 51292416 (G → C), 51292465 (T → C), 51292472 (A → T), 51292476 (T → C), 51292514 (G → A), 51292538 (TTTTC → T), 51292549 (A → G), 51292554 (CA → C), 51292556 (GCCCC → G), 51292563 (A → G), 51292716 (A → C), 51293242 (T → A), 51294803 (C → T), 51296235 (A → G), 51297245 (A → G), 51299170 (A → G), 51299810 (T → A), 51300412 (A → C), 51300803 (T → A), 51302117 (A → AT), 51302133 (G → A), 51303947 (A → ATT), 51303977 (A → G), 51304083 (G → C), 51304124 (A → AGCG), 51304166 (AG → A)
Sobic.006G066800	BEIIa, 1,4‐Alpha‐Glucan‐Branching Enzyme 2‐1	42708514 (T → TC), 42710309 (A → C), 42710479 (G → A), 42710744 (T → C), 42711487 (AT → A), 42712138 (C → T), 42712157 (T → C), 42712302 (T → C), 42712575 (A → C), 42713817 (G → A), 42713964 (G → T),42714307 (C → A), 42715316 (A → AAAC), 42716348 (G → A), 42717317 (AGG → A)
Sobic.006G221000	SS3, similar to Starch synthase IIIb‐1	56789566 (C → CCTCT), 56789696 (C → T), 56789794 (G → A), 56790054 (G → T), 56790087 (G → A), 56790140 (T → A), 56790142 (T → A), 56790147 (A → G), 56790164 (G → A)
Sobic.007G068200	similar to Starch synthase DULL1	7589674 (GGA → G), 7596076 (G → T)
Sobic.010G022600	similar to GBSS 1	1861246 (CT → C), 1861269 (G → GCTA)
Sobic.010G047700	Starch Synthase 2	3699364 (T → C), 3700536 (G → T)
Sobic.010G093400	similar to SSIIa, su2	8300714 (CTA → C), 8303592 (C → T), 8304081 (T → C)

Gene description from Campbell et al. (2016) and *Phytozome* version 12.1 *(2019)*.

### 3.4 Genome‐wide association study of ASV in sorghum

**FIGURE 5:**
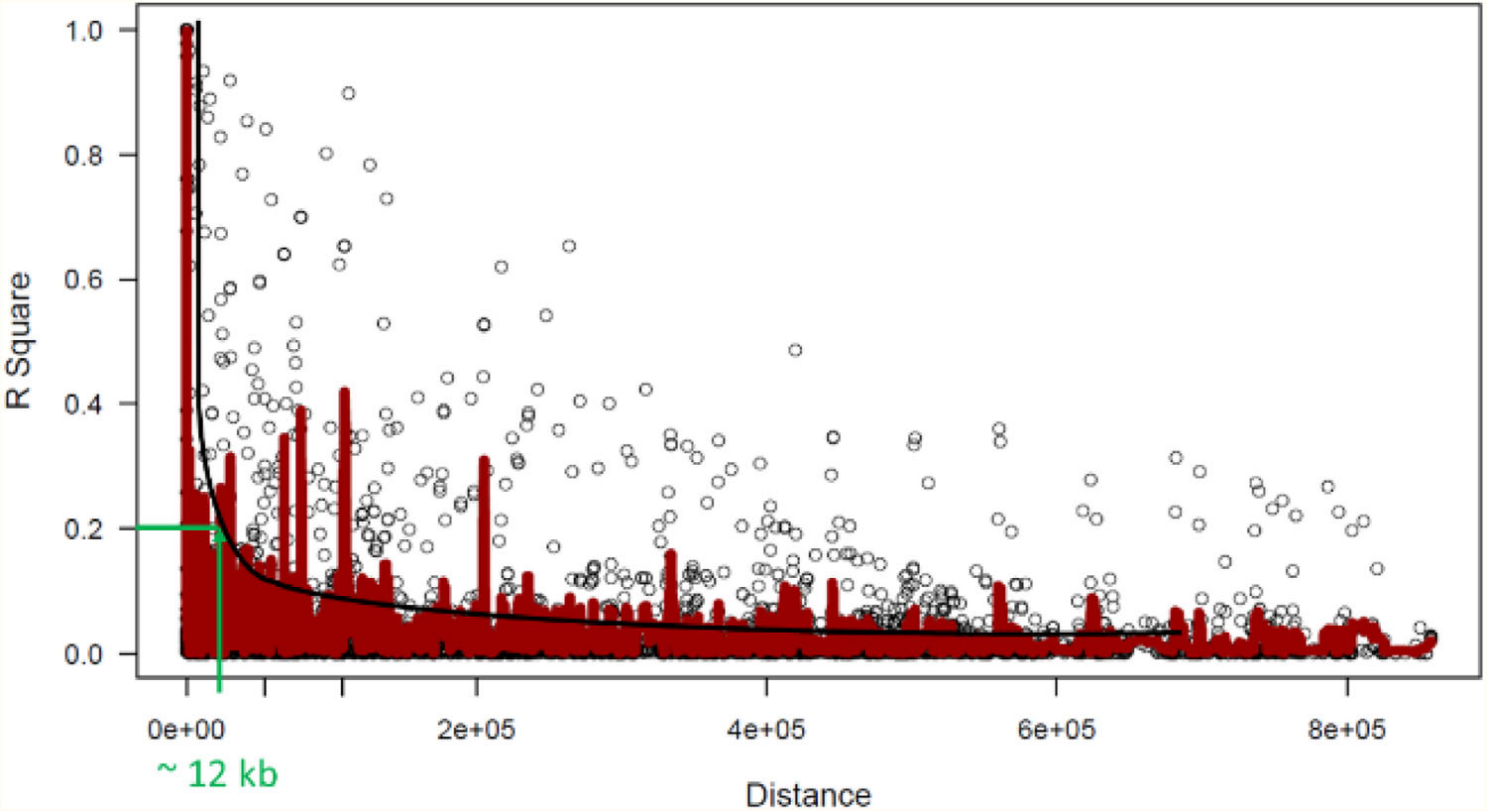
Linkage Disequilibrium (LD) decay in the sorghum diversity panel

**FIGURE 6:**
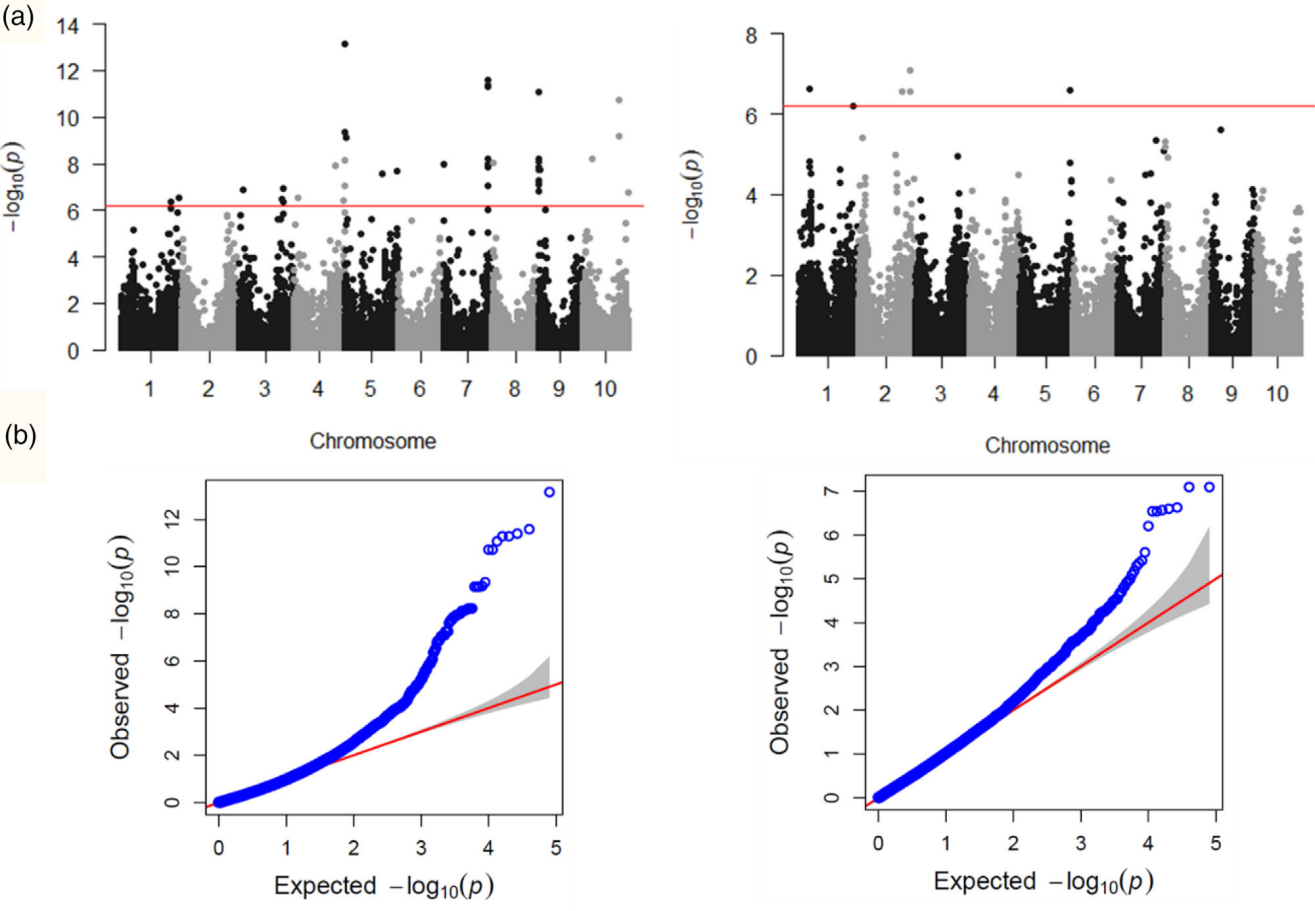
Genome Wide Association Study (GWAS) results for ASV for grain samples produced in 2013 (left) and 2017 (right) with (a) Manhattan plots and (b) QQ plots from Mixed Linear Model (MLM)

The genome wide linkage disequilibrium (LD) decay in the SDP was estimated using GAPIT and shown to be approximately 12 kb (Figure 5). GWAS using the MLM model detected 53 SNPs associated with ASV in 2013 and 8 SNPs in 2017. Using the FDR (Benjamini & Hochberg, 1995) adjusted p values at 0.05, 205 SNPs exhibited significant associations for ASV in 2013 and 14 SNPs in 2017 (Figure 6). We did not run a combined analysis across environments due to the low trait correlation of 0.23 between environments. Therefore, we report GWAS with environment specific loci associated with ASV.

All significant SNPs from 2017 and the top ten significant SNPs from 2013 including S10_60808604 and S04_67849488 were further evaluated using the integrated genome viewer (IGV; Robinson et al., 2011; Thorvaldsdóttir, Robinson, & Mesirov, 2013). The IGV was used to identify flanking genes that were approximately 15 kb up or downstream of a significant SNP, related to the estimated genome‐wide LD decay of approximately 12 kb in this study. Those genes and their annotations from phytozome (Phytozome 12, version 12.1, 2019, Goodstein et al., 2012) are listed in Table 2. One significant SNP (S10_60808604) on chromosome 10 was approximately 113 kb away from *Sobic.010G273800*, a 1,4‐alpha‐glucan branching enzyme I precursor (Table 2; Campbell et al., 2016). The SNP S10_60808604 is also flanked by two other genes, *Sobic.010G274800* (approximately 9 kb away) and *Sobic.010G275001* (approximately 15 kb away), that are predicted to be glycosyltransferases (Goodstein et al., 2012, version 12.1) and may be involved in starch biosynthesis. More work is needed to validate the role of these genes, if any, in expression of the ASV phenotype.

**TABLE 2:** GWAS results from MLM with significant SNPs and candidate genes for West Lafayette 2013 (WL13) (10 most significant SNPs and S10_60808604, S04_67849488) and 2017 (WL17) at FDR 0.05

Env.	SNP	P value	MAF	FDR adj. P value	Candidate Gene Annotation
WL13	S10_60808604	1.75E‐07	0.03	0.0003	Sobic.010G273800.2.p – similar to starch branching enzyme I precursor (Campbell et al., 2016); Sobic.010G274700.1.p ‐ (1 of 55) PF00069//PF00560//PF08263//PF13855 ‐ Protein kinase domain (Pkinase)//Leucine Rich Repeat (LRR_1)//Leucine rich repeat N‐terminal domain (LRRNT_2)//Leucine rich repeat LRR_8); Sobic.010G274800.1.p ‐ similar to Glycosyltransferase QUASIMODO1, putative, expressed: Sobic.010G274900.1.p ‐ similar to Putative uncharacterized protein; Sobic.010G275001.1.p ‐ (1 of 2) PF01697 ‐ Glycosyltransferase family 92 (Glyco_transf_92)
WL13	S05_875962	7.03E‐14	0.03	5.63E‐09	Sobic.005G010000.1.p ‐ Predicted protein; Sobic.005G010100.1.p ‐ similar to BTB/POZ domain containing protein, expressed; Sobic.005G010200.1.p ‐ similar to Hypoxia induced protein conserved region containing protein, expressed
WL13	S05_875955	4.60E‐10	0.08	4.10E‐06	
WL13	S07_59182141	2.61E‐12	0.05	8.18E‐08	Sobic.007G157400.2.p ‐ (1 of 8) 4.2.1.105 ‐ 2‐hydroxyisoflavanone dehydratase; Sobic.007G157500.1.p ‐ similar to Putative uncharacterized protein; Sobic.007G157550.1.p ‐ (1 of 5) PTHR23024//PTHR23024:SF137 ‐ Member ‘GDXG’ Family of Lipolytic enzymes; Sobic.007G157600.1.p ‐ Predicted protein; Sobic.007G157700.1.p ‐ similar to NADPH HC toxin reductase
WL13	S09_1041464	8.27E‐12	0.03	1.10E‐07	Sobic.009G011600 ‐ no annotation; Sobic.009G011700.1.p – similar to Putative uncharacterized protein; Sobic.009G011400.1.p – similar to Putative uncharacterized protein; Sobic.009G011500.1.p – Predicted protein; Sobic.009G011600 no annotation; Sobic.009G011700.1.p – similar to Putative uncharacterized protein
WL13	S07_59500280	4.08E‐12	0.05	8.18E‐08	Sobic.007G160300.1.p ‐ similar to D‐type cyclin; Sobic.007G160400.1.p ‐ similar to Putative uncharacterized protein
WL13	S07_59224740	5.10E‐12	0.05	8.18E‐08	Sobic.007G158000.1.p ‐ weakly similar to Putative uncharacterized protein
WL13	S07_59224782				
WL13	S10_48542785	1.87E‐11	0.03	1.88E‐07	Sobic.010G164200.1.p ‐ similar to Putative uncharacterized protein; Sobic.010G164300.1.p ‐ (1 of 2) PTHR23041:SF65 ‐ E3 UBIQUITIN‐PROTEIN LIGASE CCNB1IP1 HOMOLOG
WL13	S10_48542812	1.87E‐11	0.03	1.88E‐07	
WL13	S10_48403358	6.62E‐10	0.04	4.36E‐06	Sobic.010G164100.1.p ‐ (1 of 1) PTHR19139//PTHR19139:SF183 ‐ AQUAPORIN TRANSPORTER
WL13	S04_67849488	7.15E‐09	0.07	3.10E‐05	Sobic.004G349500.1.p ‐ Similar to Plant integral membrane protein TIGR01569 containing protein expressed Sobic.004G349600.1.p – similar to MATE efflux protein like Sobic.004G349650.1 – no annotation; Sobic.004G349800.2‐ no annotation
WL17	S02_70502500	8.07E‐08	0.07	0.003	Sobic.002G337700.1.p ‐ similar to Putative uncharacterized protein; Sobic.002G337800.1.p ‐ similar to Myb protein
WL17	S02_70502518	8.07E‐08	0.07		
WL17	S02_70502913	2.84E‐07	0.06		
WL17	S02_70502916	2.84E‐07	0.06		
WL17	S01_15639633	2.31E‐07	0.03	0.003	Sobic.001G183550.1 – no annotation Sobic.001G183600.1.p ‐ similar to Putative uncharacterized protein Sobic.001G183633.1 – no annotation Sobic.001G183666.1 – no annotation
WL17	S02_59605124	2.66E‐07	0.03	0.003	Sobic.002G204400.1.p ‐ similar to Putative uncharacterized protein; Sobic.002G204500.1.p ‐ similar to Phospholipase D lambda; Sobic.002G204600.1.p ‐ weakly similar to Putative uncharacterized protein; Sobic.002G204700.1.p ‐ (1 of 5) PTHR23067//PTHR23067:SF47 ‐ Double‐stranded RNA‐Binding ZINC FINGER
WL17	S05_68193148	2.54E‐07	0.04	0.003	Sobic.005G196600.1.p ‐ Predicted protein; Sobic.005G196700.1.p ‐ similar to Sugar transporter family protein, expressed; Sobic.005G196800.1.p ‐ similar to Os12g0514100 protein; Sobic.005G196900.3.p ‐ similar to Potyvirus VPg interacting protein, putative, expressed; Sobic.005G196950.1.p ‐ similar to Os12g0514600 protein
WL17	S01_73843118	6.23E‐07	0.05	0.006	Sobic.001G464200.1.p ‐ similar to XPG I‐region family protein, expressed; Sobic.001G464300.1.p ‐ similar to Helix‐loop‐helix DNA‐binding domain containing protein; Sobic.001G464400.3.p ‐ similar to Putative uncharacterized protein; Sobic.001G464500.1.p ‐ similar to CUE domain containing protein, expressed; Sobic.001G464600.1.p ‐ similar to Transposon protein, putative, unclassified, expressed
WL17	S09_11993119	2.52E‐06	0.35	0.022	Sobic.009G082400.1 – no annotation; Sobic.009G082500.1 – no annotation; Sobic.009G082550.1 – no annotation
WL17	S02_5699759	3.79E‐06	0.07	0.030	Sobic.002G058900.1.p ‐ Predicted protein; Sobic.002G059000.1.p ‐ (1 of 303) PF00646 ‐ F‐box domain (F‐box); Sobic.002G059100.2 – no annotation; Sobic.002G059200.1.p ‐ similar to Histone H2A
WL17	S07_51741290	4.39E‐06	0.04	0.032	Sobic.007G120000.1.p ‐ similar to Os08g0398300 protein
WL17	S08_253410	5.01E‐06	0.20	0.033	Sobic.008G002600.1.p ‐ (1 of 1) PTHR10209//PTHR10209:SF143 ‐Oxidoreductase, 2OG‐FE II Oxygenase family; Sobic.008G002700.1.p ‐ Predicted protein; Sobic.008G002800.1.p ‐ similar to Fatty acid desaturase DES1; Sobic.008G002950.1.p ‐ (1 of 34) PF13365 ‐ Trypsin‐like peptidase domain (Trypsin_2)
WL17	S08_274611	6.67E‐06	0.18	0.041	Sobic.008G003100.2.p ‐ (1 of 34) PF13365 ‐ Trypsin‐like peptidase domain (Trypsin_2); Sobic.008G003200.1.p ‐ similar to Putative fatty acid desaturase
WL17	S07_62928259	8.11E‐06	0.08	0.046	Sobic.007G197700.1.p ‐ similar to BHLH transcription factor(GBOF‐1)‐like; Sobic.007G197800.1.p ‐ similar to Os09g0502000 protein; Sobic.007G197900.1 – no annotation; Sobic.007G198000.1.p ‐ similar to Os08g0524400 protein

### 3.5 ASV exhibits recessive inheritance

SC489, SC491, SC587 and SC589 exhibited stable expression of the ASV+ phenotype. These donors were crossed to ATx623 (cytoplasm sterile) and seeds from the crosses were evaluated to determine if the ASV is inherited as a dominant or recessive trait (Table 3). Very few of the hybrid seeds exhibited the ASV+ phenotype indicating that ASV is inherited as a recessive trait.

**TABLE 3:** Ratio of seeds exhibiting ASV+ phenotypes in crosses between SC489, SC491, SC587 and SC589 with ATx623

Environment	Pedigree	Ratio of Seeds Exhibiting ASV+ to ASV‐^a^
2018	ATx623/BTx623	0:32
2018	ATx623/SC489	1:31
2018	ATx623/SC491	1:31
2018	ATx623/SC587	0:32
2018	ATx623/SC589	0:32

^a^ ASV 1.8% KOH, 24 h.

### 3.6 Instability of the ASV+ phenotype

Four SC lines were evaluated for ASV in 2017, and 2018 (Table 4). These lines exhibited the ASV+ phenotype in each year; however, the expression of the ASV+ phenotype was variable over years. For lines exhibiting the ASV+ phenotype, fewer than 22 seeds of 32 seeds tested exhibited the ASV+ phenotype depending on the individual environment, panicle source, or season. Genotypes exhibiting the ASV‐ phenotype exhibited a consistent phenotype over years.

**TABLE 4:** Evaluation of the ASV phenotype of SC489, SC491, SC587 and SC589 and controls in West Lafayette 2017 and 2018

Environment	Pedigree	Ratio of Seeds Exhibiting ASV+ to ASV‐ ^a^
2017	Tx623	0:32
2017	Macia	0:32
2017	Sepon82	0:32
2017	SC 489	19:13
2017	SC 491	17:15
2017	SC 587	8:24
2017	SC 589	11:21
2018	Tx623	0:32
2018	Macia	0:32
2018	Sepon82	0:32
2018	SC 489	13:19
2018	SC 491	10:22
2018	SC 587	18:14
2018	SC 589	16:16

Samples used in the starch analysis.

^a^ Ratio of seeds exhibiting ASV+:ASV‐ phenotype in 2 to 4 panicles tested.

### 3.7 Physiochemical analysis

NIRS predictions indicated that SC489, SC491, SC587, and SC589 and controls exhibited total starch (db) values ranging between 70% and 80% and protein (db) between 9% and 14% in 2013 and 2017 with values being slightly higher in 2017 (Table 5).

**TABLE 5:** Near‐infrared spectroscopy predicted moisture, starch, and protein contents of grain samples from sorghum landraces from 2013 and 2017

	2013			2017		
	Moisture	Starch	Protein	Moisture	Starch	Protein
Pedigree	(%)	(db)	(db)	(%)	(db)	(db)
SC489	11.2 B	77.4 AB	10.5 AB	11.4 ABC	80.1 A	11.0 BC
SC491	10.5 C	76.1 AB	9.3 B	11.4 AB	79.4 AB	12.1 B
SC587	14.3 A	79.8 A	9.3 B	11.5 AB	80.7 A	10.9 BC
SC589	10.8 BC	77.8 AB	10.1 AB	11.6 A	77.7 BC	13.5 A
Macia	10.9 BC	72.2 B	11.0 A	11.3 ABC	76.6 C	11.1 BC
Sepon82	10.9 BC	72.7 B	10.9 AB	11.2 BC	77.4 BC	10.8 BC
T×623	10.8 BC	75.9 AB	10.9 AB	11.1 C	79.2 AB	10.0 C

2013 ‐ one biological replication and two technical replications.

**FIGURE 7:**
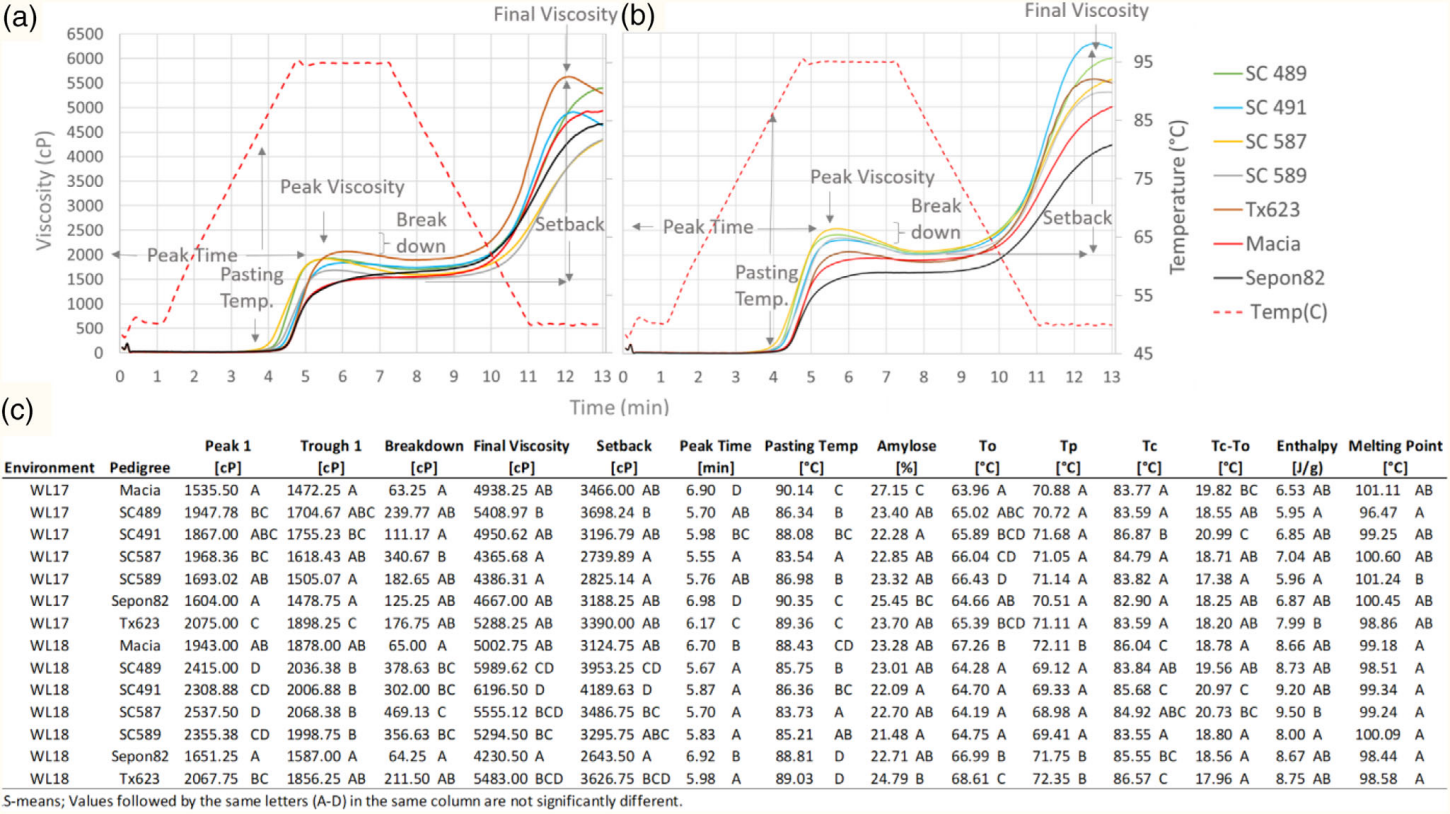
Starch characteristics of sorghum accessions with contrasting ASV phenotypes ‐ (a) paste viscosity profiles in 2017, (b) paste viscosity profiles in 2018, and (c) starch thermal properties and physiochemical characteristics

G × E interactions were detected for amylose, starch thermal properties, and paste viscosity profiles (Figure 7). The amylose values of the four SC‐lines varied between approximately 21% and 24% with the ASV+ lines being numerically lower than the controls in 2017 and 2018 (Figure 7). Significant differences for amylose were observed only for SC489, SC587 and SC589 in the season 2017 in comparison to Macia and for SC491 and SC589 in 2018 in comparison to Tx623. The starch thermal properties for SC489, SC491, SC587, and SC589 were similar to the control genotypes in 2017 but exhibited significantly lower starch GT for onset (T_o_) and peak (T_p_) in 2018 (Figure 7).

Evaluation of paste viscosity behavior showed similar profiles of the four SC‐lines in comparison to the controls (Figure 7). However, Macia and Sepon82, behaved differently than Tx623 and the SC lines and exhibited no breakdown and lower final viscosity in 2018.

## 4 DISCUSSION

The sorghum diversity panel (SDP) included nearly 800 sorghum conversion lines and represents the genetic diversity of the crop (Stephens et al., 1967; Rosenow et al., 1997a, 1997b). The SDP is larger than the commonly used Sorghum Association Panel (SAP) (Adeyanju et al., 2015; Boyles et al., 2017; Casa et al., 2008; Cuevas et al., 2017; Mace et al., 2013; Morris et al., 2013; Shenstone et al., 2018; Sukumaran et al., 2012). The race and regional classification from our principal component analysis confirmed previous reports on the different sorghum races and geographic origins. The SDP is well‐represented by durra types and durra‐bicolor types from India and Eastern Africa, guinea types from Western Africa, kafir types from Eastern and Southern Africa, and caudatum types from diverse environments across Africa (Morris et al., 2013; Billot et al., 2013; Sukumaran et al., 2012; Harlan & de Wet, 1972; Dahlberg, 2000). The bicolor types exhibited no clear geographical classification (Morris et al., 2013; Deu et al., 2006; Brenton et al., 2016; Billot et al., 2013).

Recent genetic studies of sorghum EMS mutants demonstrated the ASV phenotype was generally stable across years and locations (Griebel et al., 2019b). The pattern of expression for ASV was less consistent in the SDP. The ASV phenotyping experiment in 2013 was set up as an exploratory study and 24 accessions exhibiting the ASV trait were identified (Figure 3). Follow‐up studies of these accessions indicated more variable expression of ASV so the phenotyping study of the sorghum diversity panel was repeated in 2017 with eight seeds per genotype. Thirteen accessions exhibited the ASV+ phenotype across two growing seasons (Figure 4). Analyses of genetic diversity confirmed that 11 of these SC lines cluster together as durras representing the Nandyal working group originating from India (Figure 2). The other lines exhibiting a stable ASV+ phenotype included a guinea from Western Africa and a line of no clear race nor geographic classification.

The race durra is reported as having ovate panicles, produce little hairs, and are very stiff and densely packed in comparison to the bicolors (Figure 4) (Dahlberg, 2000). The durra race is sub‐divided into the working groups 50, 51 and 52 (Dahlberg, 2000). The working group 51 describes durra types named Nandyal (Dahlberg, 2000). The Nandyal types are reported as short season durras (Harlan & de Wet, 1972) producing bold, glutinous grains (Golomb et al., 1976). The Nandyal sorghums included in this study exhibited densely packed panicles, creamy to yellow seed color, a strong glutinous and pearly endosperm, and medium to tall plant size (Dahlberg, 2000).

Glutinous seeds are often called waxy (Martin & Jenkins, 1950). An earlier study by Waniska (1976) was able to distinguish between waxy and non‐waxy sorghum genotypes by treating the whole, pearled sorghum kernels with NaOH. Glutinous rice is desired in many Asian cultures and countries (Roder, Keoboulapha, Vannalath, & Phouaravanh, 1996) as it becomes soft and sticky after cooking (Wang et al., 2007; Golomb, 1976; Martin & Jenkins, 1950), slightly sweet (Golomb et al., 1976) and tender and glossy when eating, which is a result of low amylose content, low starch GT (Wang et al., 2007) and high gel consistency (Wang et al., 2007; Golomb et al., 1976). The four selected sorghum genotypes SC489, SC491, SC587 and SC589 exhibited similar physiochemical behaviours with slightly lower amylose content, high gel consistency and in some seasons a slightly lower starch GT (Figure 7). These sorghums may be suitable for use in sweet dishes and alcoholic beverages and less suitable for non‐sticky and dishes where less viscosity is desired (Golomb et al., 1976).

The ASV trait has been shown to be controlled by the *ALK* gene, a starch synthase (SS) II in rice (Gao et al., 2011; Wang et al., 2007; Tian et al., 2009) and by a SSIIa and SBEIIb in sorghum (Griebel et al., 2019b). SC489, SC491, SC587 and SC589 have several unique SNPs in starch biosynthesis genes compared to Tx623 that may be involved in the expression of the ASV+ phenotype (Table 1). The 107 SNPs in common in the four lines are not necessarily due to the ASV+ phenotype but due to the close relatedness of the four lines from the same working group (Figure 4c). However, of the 107 SNPs, 59 are in starch branching genes, one being *SBEIIb*, reported previously as a key gene causing ASV in sorghum (Griebel et al., 2019a). Several genes control amylose content and gel consistency (GC) in rice (Tian et al., 2009; Gao et al., 2011). Genes associated with amylose content in rice include *Wx, ALK, SSIII‐2, SSI, PUL* and for gel consistency *ISA, SBE3* (Tian et al., 2009). In sorghum, ASV and starch GT are influenced by *SSIIa* and *SBEIIb* (Griebel et al., 2019b). The physiochemical behaviour of the four SC lines in the current study is similar to our previous study, where low amylose content, high gel consistency, and lower starch GT was observed in sorghum *ssIIa* mutants (Griebel et al., 2019b). The previously reported information on genetics and physiochemical behaviour could help to narrow down the putative candidate genes in the current study and may point to the gene that causes the ASV variation in the SDP.

This is the first GWAS reported on the ASV phenotype in the standing variation of sorghum. The GWAS for ASV in sorghum produced results similar to studies in rice with poor trait correlations and inconsistent performance across years (Zhao et al., 2011). This might be influenced by fewer kernels tested in 2013 than in 2017, which could be evaluated in a follow up study in multiple environments and with a larger number of seeds per genotype being tested. The LD decay in rice is slower (Huang et al., 2010) than in sorghum with reports of LD ranging from 1 kb up to 20 kb (Figure 5; Morris et al., 2013; Mace et al., 2013). However, some candidate genes in rice were close to the *ALK (SSII‐3)* gene (Huang et al., 2010; Zhao et al., 2011) and the *SSII‐2* (Zhao et al., 2011). In this study, the GWAS results do not show a significant SNP in close proximity to *Sobic.004G163700*, a *SBEIIb*, and *Sobic.010G093400*, a *SSIIa*, reported as the causal genes for ASV in sorghum (Griebel et al., 2019b). The GWAS identified three candidate genes for ASV. The SNP S10_60687678 was 9 kb away from *Sobic.010G274800*, 15 kb away from *Sobic.010G275001*, and 113 kb away from *Sobic.010G273800* (Table 3). *Sobic.010G273800 is* annotated as a 1,4‐alpha glucan branching enzyme I precursor (Table 3) and a known gene involved in starch biosynthesis in the amyloplast (Campbell et al., 2016). The other two genes not reported by Campbell et al. (2016) are annotated as glycosyltransferases (Phytozome 12, version 12.1, 2019; Goodstein et al., 2012). These could be sorghum genes involved in starch biosynthesis and are candidates for further evaluation. The common enzymes involved in starch biosynthesis are starch synthases, which are ADP‐glucose‐dependent (alpha 1,4‐glucan‐4‐glucosyl transferases; Boyer, 1985) transferases, and starch branching enzymes (SBEs) being 1,4‐alpha‐glucan 6‐glucosyl transferases (Tetlow & Emes, 2014; Boyer, 1985).

## 5 CONCLUSIONS

Variation in the ASV phenotype can be found in the standing variation of sorghum. The accessions that exhibit stable ASV across seasons are mainly *durra* types from the working group Nandyal described in previous studies as glutinous varieties. The four selected SC lines exhibited normal amylose content, high GC, and environmentally dependent lower starch GT. These starch characteristics may be valuable for the food industry for product development with different levels of stickiness and viscosity. GWAS showed significant associations for one SNP in proximity to a starch biosynthesis gene *Sobic.010G273800* and two candidate genes *Sobic.010G274800* and *Sobic.010G275001* described as glucosyltransferases. Whole genome resequencing (WGS) revealed several SNPs in genes in the starch biosynthesis pathway that need to be further evaluated. We do recommend a follow up study in additional environments and with a larger number of seeds being tested per genotype to evaluate the effect of growth environment on the ASV expression in the sorghum diversity panel. However, with more than 800 lines it might be an elaborate and costly work to do even so the ASV test itself is cheap.

## AUTHOR CONTRIBUTIONS

S.G. designed and performed all areas of research, M.R.T. contributed seeds for the sorghum diversity population, A.A. helped with genotyping, S.G. wrote the manuscript; coauthors reviewed and edited the manuscript, and M.R.T. served as the major advisor and project lead.

## CONFLICT OF INTEREST

None.

## Supporting information

Supplemental Material

## Data Availability

The ASV phenotypic data used in GWAS studies are provided in Table S1. The imputed genotype‐by‐sequencing data were kindly provided by Dr. Patrick Brown and raw data are available at University of Illinois database, https://doi.org/10.13012/B2IDB-7570206_V1 (P. Brown, personal communication, 2019; Brown, 2017).
