# Supplementary material for: Genetic diversity for starch quality and alkali spreading value in sorghum
Source: Plant Genome. 2020 Dec 1;14(1):e20067. doi: 10.1002/tpg2.20067 (PMC12899596; doi:10.1002/tpg2.20067)
Supplement: Supplementary file 1 [file TPG2-14-e20067-s001.docx]

**Supplemental Information**

**Table S.**1: Genotypes used in PCA by race, country and ASV (Race, PI name, country: Klein, personal communication, 2019).

| **Taxa and SC code** | **PI name** | **Race** | **ASV-WL13** | **ASV-WL17** | **Country** | **Regions** | **PC1** | **PC2** | **PC3** | **PC4** | **PC5** |
| --- | --- | --- | --- | --- | --- | --- | --- | --- | --- | --- | --- |
| Ajabsido | PI656015 | Breeding-line | 0 | 0 | Breeding-line | Breeding-line | -21.875 | 35.080 | -18.876 | 3.632 | -16.180 |
| BOK11 | PI656002 | Breeding-line | 0 | 0 | Breeding-line | Breeding-line | -1.593 | -10.010 | 135.223 | -12.457 | -38.618 |
| BQL41 | PI656063 | Breeding-line | 0 | 0 | Breeding-line | Breeding-line | -4.560 | 7.637 | 54.734 | -5.500 | -24.400 |
| BTx2752 | PI656018 | Breeding-line | 0 | 0 | Breeding-line | Breeding-line | -5.893 | -0.766 | 57.806 | -8.203 | -17.913 |
| BTx3042 | PI656019 | Breeding-line | 0 | 0 | Breeding-line | Breeding-line | 18.511 | -3.942 | 90.199 | -11.390 | -66.096 |
| BTx3197 | PI655992 | Breeding-line | 0 | 0 | Breeding-line | Breeding-line | -1.500 | -8.957 | 140.438 | -14.179 | -43.557 |
| BTx378 | PI655991 | Breeding-line | 0 | 0 | Breeding-line | Breeding-line | 6.088 | -8.106 | 131.600 | -14.530 | -47.273 |
| BTx399 | PI655993 | Breeding-line | 0 | 0 | Breeding-line | Breeding-line | -24.450 | 27.740 | 42.397 | -4.621 | -42.785 |
| BTx615 | PI656022 | Breeding-line | 0 | 0 | Breeding-line | Breeding-line | -12.770 | 4.841 | 15.165 | -5.301 | -10.685 |
| BTx623 | PI564163 | Breeding-line | 0 | 0 | Breeding-line | Breeding-line | -34.361 | 28.206 | 60.357 | -11.062 | -35.668 |
| BTx641 | PI642793 | Breeding-line | 0 | 0 | Breeding-line | Breeding-line | -13.801 | 15.760 | 50.777 | -6.564 | -47.616 |
| BTx642 | PI656029 | Breeding-line | 0 | 0 | Breeding-line | Breeding-line | 47.733 | 15.392 | 12.306 | 7.792 | -57.738 |
| BTx643 | NA | Breeding-line | 1 | 0 | Breeding-line | Breeding-line | -27.834 | 20.802 | 47.308 | -8.987 | -35.476 |
| BTx645 | NA | Breeding-line | 0 | 0 | Breeding-line | Breeding-line | -20.711 | 23.095 | 53.893 | -7.579 | -47.704 |
| BTxARG-1 | PI561072 | Breeding-line | 0 | 0 | Breeding-line | Breeding-line | -0.815 | -9.756 | 132.639 | -11.346 | -39.101 |
| Caprock | PI655986 | Breeding-line | 0 | NA | Breeding-line | Breeding-line | 24.824 | 2.449 | 70.938 | -9.858 | -66.461 |
| Comb7078 | PI655990 | Breeding-line | 0 | 0 | Breeding-line | Breeding-line | 44.027 | 12.897 | 23.145 | -3.674 | -68.557 |
| Day | PI641874 | Breeding-line | 0 | 0 | Breeding-line | Breeding-line | 34.708 | 12.355 | 29.174 | -1.129 | -58.813 |
| Dorado | PI656034 | Breeding-line | 0 | 0 | Breeding-line | Breeding-line | -72.129 | 66.900 | -14.701 | -3.445 | -9.518 |
| DwfYellMilo | NA | Breeding-line | 0 | 0 | Breeding-line | Breeding-line | 54.617 | 21.040 | -17.112 | 3.388 | -70.371 |
| ElMota | PI656035 | Breeding-line | 0 | 0 | Breeding-line | Breeding-line | -53.617 | 43.044 | -19.484 | -1.055 | -3.988 |
| FeteritaGishesh | PI152651 | Breeding-line | 0 | 0 | Breeding-line | Breeding-line | -62.425 | 47.495 | -12.976 | -0.577 | -14.020 |
| HEGARI | PI34911 | Breeding-line | 0 | 0 | Breeding-line | Breeding-line | -75.775 | 72.084 | -24.603 | -1.994 | -10.693 |
| KS19 | PI655998 | Breeding-line | 0 | 0 | Breeding-line | Breeding-line | -22.487 | -51.836 | 30.371 | -10.227 | -54.239 |
| MR732 | PI656051 | Breeding-line | 0 | 0 | Breeding-line | Breeding-line | -10.450 | 7.357 | 16.037 | -3.669 | -19.645 |
| Macia | PI565121 | Breeding-line | 0 | 0 | Breeding-line | Breeding-line | -68.689 | 62.414 | -17.258 | -2.605 | -11.346 |
| Malisor84_7 | PI656048 | Breeding-line | 0 | 1 | Breeding-line | Breeding-line | -60.301 | 48.161 | 24.464 | -8.666 | -24.263 |

Table S4.1. continued

| Martin | PI655987 | Breeding-line | 0 | 0 | Breeding-line | Breeding-line | 15.076 | 3.054 | 119.591 | -10.798 | -74.018 |
| --- | --- | --- | --- | --- | --- | --- | --- | --- | --- | --- | --- |
| P898012 | PI656057 | Breeding-line | 0 | 0 | Breeding-line | Breeding-line | -66.453 | 64.036 | -22.252 | -1.127 | -10.547 |
| P9517 | PI656058 | Breeding-line | 0 | 0 | Breeding-line | Breeding-line | -27.006 | 12.658 | 66.223 | -7.877 | -18.353 |
| P_721 | PI656055 | Breeding-line | 0 | 0 | Breeding-line | Breeding-line | -56.163 | 53.130 | 9.406 | -4.807 | -35.892 |
| RTAM2566 | PI655977 | Breeding-line | 0 | 0 | Breeding-line | Breeding-line | -59.853 | 67.275 | -20.517 | 0.571 | -19.361 |
| RTAM428 | PI656009 | Breeding-line | 0 | 0 | Breeding-line | Breeding-line | -65.194 | 67.423 | 1.447 | -0.483 | -32.820 |
| RTX2536 | PI656010 | Breeding-line | 0 | 0 | Breeding-line | Breeding-line | -23.155 | -41.395 | -9.023 | -5.710 | -40.448 |
| RTX2737 | PI655978 | Breeding-line | 0 | 0 | Breeding-line | Breeding-line | -11.199 | -39.514 | 5.946 | -6.069 | -39.197 |
| RTx2783 | PI656001 | Breeding-line | 0 | 0 | Breeding-line | Breeding-line | -42.269 | 50.014 | 6.871 | 0.249 | -26.471 |
| RTx2917 | PI629040 | Breeding-line | 0 | 0 | Breeding-line | Breeding-line | -10.459 | 16.511 | 19.960 | -7.198 | -38.448 |
| RTx430 | PI655996 | Breeding-line | 0 | 0 | Breeding-line | Breeding-line | -33.151 | 13.121 | 35.916 | -6.793 | -34.402 |
| RTx434 | PI564165 | Breeding-line | 0 | 0 | Breeding-line | Breeding-line | -17.871 | 43.912 | 11.575 | -1.411 | -48.999 |
| RTx436 | PI561071 | Breeding-line | 0 | 0 | Breeding-line | Breeding-line | -20.894 | 11.638 | 16.947 | -4.897 | -46.331 |
| RTx437 | PI629034 | Breeding-line | 0 | 1 | Breeding-line | Breeding-line | -46.875 | 17.553 | -6.859 | -6.914 | -27.553 |
| Redbine | PI655989 | Breeding-line | 0 | 0 | Breeding-line | Breeding-line | 16.154 | 2.615 | 102.867 | -11.185 | -67.717 |
| SC0002 | PI534118 | Durra | 0 | 1 | Ethiopia | Eastern-Africa | 43.003 | -3.698 | -8.501 | 132.728 | 19.793 |
| SC0003 | NA | Durra | 0 | 0 | Ethiopia | Eastern-Africa | 34.101 | -3.170 | 42.149 | -1.126 | -40.614 |
| SC0004 | PI534119 | Durra | 0 | 0 | Ethiopia | Eastern-Africa | 65.970 | 5.571 | -10.631 | -4.087 | 17.177 |
| SC0006 | PI533902 | Durra-Bicolor | 0 | 0 | Ethiopia | Eastern-Africa | 41.219 | -0.535 | -7.381 | 142.795 | 10.708 |
| SC0007 | PI534120 | Durra-Bicolor | 0 | 0 | Ethiopia | Eastern-Africa | 39.544 | -1.573 | -4.188 | 155.127 | 8.661 |
| SC0010 | PI534121 | Durra-Bicolor | 0 | 0 | Ethiopia | Eastern-Africa | 45.880 | 7.260 | -13.736 | 9.575 | -27.390 |
| SC0012 | PI534122 | Bicolor | 0 | 0 | Ethiopia | Eastern-Africa | 33.003 | -15.308 | -7.582 | 145.899 | 38.153 |
| SC0013 | PI534123 | Durra-Bicolor | 0 | 0 | Ethiopia | Eastern-Africa | 29.172 | -2.078 | 4.936 | 122.018 | 0.179 |
| SC0015 | PI534124 | Bicolor | 0 | 0 | Ethiopia | Eastern-Africa | 16.746 | 1.118 | -4.967 | 113.761 | 3.514 |
| SC0016 | PI534125 | Bicolor | 0 | 0 | Ethiopia | Eastern-Africa | 16.903 | -5.912 | -0.453 | 59.756 | 18.771 |
| SC0017 | PI533903 | Bicolor | 0 | 0 | Ethiopia | Eastern-Africa | 26.694 | -14.469 | -2.977 | 100.114 | 31.394 |
| SC0019 | PI533904 | Bicolor | 0 | 0 | Ethiopia | Eastern-Africa | 30.116 | -12.250 | -4.936 | 119.303 | 30.606 |
| SC0020 | PI534126 | Bicolor | 0 | 0 | Ethiopia | Eastern-Africa | 37.312 | -9.008 | -4.541 | 153.059 | 16.287 |
| SC0021 | PI534127 | Kafir-Bicolor | 0 | 0 | Ethiopia | Eastern-Africa | 40.872 | 3.792 | -1.562 | 159.052 | -5.635 |
| SC0022 | PI656091 | Durra | 0 | 1 | Ethiopia | Eastern-Africa | 52.254 | 6.249 | -13.908 | 3.782 | -28.432 |
| SC0023 | PI534128 | Durra | 0 | 0 | Ethiopia | Eastern-Africa | 10.017 | 8.790 | -11.495 | 0.489 | -3.913 |
| SC0024 | PI534129 | Durra | 1 | 0 | Ethiopia | Eastern-Africa | 56.801 | 13.574 | -2.219 | 8.267 | -49.081 |

Table S4.1. continued

| SC0025 | PI656092 | Durra | 0 | 0 | Ethiopia | Eastern-Africa | 53.909 | 4.687 | -9.683 | -0.610 | -23.727 |
| --- | --- | --- | --- | --- | --- | --- | --- | --- | --- | --- | --- |
| SC0027 | NA | Durra-Bicolor | 0 | 0 | Ethiopia | Eastern-Africa | 57.295 | 6.447 | -17.214 | 63.552 | -27.231 |
| SC0028 | PI534130 | Durra | 0 | 0 | Ethiopia | Eastern-Africa | 57.238 | 8.009 | -19.042 | 5.150 | -27.284 |
| SC0029 | PI533905 | Durra | 0 | 0 | Ethiopia | Eastern-Africa | 57.014 | 13.193 | -8.187 | 10.071 | -58.525 |
| SC0030 | PI534131 | Durra-Bicolor | 0 | 0 | Ethiopia | Eastern-Africa | 46.640 | 2.866 | 1.487 | 104.866 | -16.426 |
| SC0031 | PI533906 | Durra | 0 | 0 | Ethiopia | Eastern-Africa | 37.478 | 6.922 | -3.577 | 1.042 | -15.520 |
| SC0033 | PI534132 | Durra | 0 | 0 | Ethiopia | Eastern-Africa | 53.594 | 4.371 | -4.307 | 0.819 | -22.966 |
| SC0035 | PI534133 | Durra | 0 | 0 | Ethiopia | Eastern-Africa | 60.288 | 5.729 | -20.621 | 4.293 | -26.657 |
| SC0036 | PI533907 | Durra | 0 | 0 | Ethiopia | Eastern-Africa | 62.399 | 9.022 | -18.221 | 1.801 | -27.467 |
| SC0037 | PI534134 | Durra-Bicolor | 0 | 0 | Ethiopia | Eastern-Africa | 41.370 | -3.244 | -6.661 | 156.277 | 13.527 |
| SC0038 | PI534135 | Durra | 1 | 0 | Ethiopia | Eastern-Africa | 60.275 | 4.660 | -26.763 | 3.363 | -17.622 |
| SC0041 | NA | Durra | 0 | 0 | Ethiopia | Eastern-Africa | 15.543 | 4.547 | 1.772 | -2.004 | -13.478 |
| SC0042 | PI576393 | Caudatum | 0 | 0 | Ethiopia | Eastern-Africa | -26.085 | -3.919 | 16.466 | 20.569 | 13.025 |
| SC0043 | PI534136 | Durra-Bicolor | 0 | 0 | Ethiopia | Eastern-Africa | 42.440 | 3.004 | -11.930 | 154.497 | 3.324 |
| SC0044 | PI533908 | Bicolor | 0 | 0 | Ethiopia | Eastern-Africa | 34.895 | -14.684 | -7.456 | 148.827 | 42.494 |
| SC0048 | PI533909 | Caudatum | 0 | 0 | Sudan | Northern-Africa | -39.469 | 34.906 | -6.151 | -3.876 | -2.609 |
| SC0049 | PI656098 | Guinea | 0 | 0 | Sudan | Northern-Africa | -31.757 | 25.552 | -6.149 | 6.216 | -8.330 |
| SC0050 | PI533787 | Guinea-Caudatum | 0 | 0 | Sudan | Northern-Africa | -23.533 | 6.977 | -8.086 | 10.902 | -6.856 |
| SC0051 | PI534137 | Caudatum | 0 | 0 | Sudan | Northern-Africa | -32.456 | 24.592 | -16.450 | -4.183 | 1.968 |
| SC0052 | PI533830 | Caudatum | 0 | 0 | Sudan | Northern-Africa | -29.888 | 27.480 | -19.388 | 1.809 | 7.812 |
| SC0053 | PI533788 | Caudatum | 0 | 0 | Sudan | Northern-Africa | -2.303 | 23.538 | -28.112 | -11.722 | 35.106 |
| SC0054 | PI533960 | Caudatum | 0 | 0 | Sudan | Northern-Africa | -22.938 | 16.819 | -15.357 | -2.612 | 10.319 |
| SC0055 | PI533755 | Caudatum | 0 | 0 | Sudan | Northern-Africa | -24.757 | 18.797 | -24.018 | -0.387 | 15.039 |
| SC0056 | PI533910 | Caudatum | 0 | 0 | Sudan | Northern-Africa | -36.338 | 33.752 | -6.141 | -3.711 | 2.920 |
| SC0057 | PI533789 | Guinea-Caudatum | 0 | 0 | Sudan | Northern-Africa | -33.490 | 20.711 | -22.469 | -0.718 | 18.995 |
| SC0058 | PI533911 | Caudatum | 0 | 0 | Sudan | Northern-Africa | -39.969 | 23.469 | -13.646 | -1.150 | 20.140 |
| SC0059 | PI656102 | Caudatum-Bicolor | 0 | 0 | Sudan | Northern-Africa | -10.814 | -3.523 | 22.632 | 6.749 | 4.151 |
| SC0060 | PI533962 | Caudatum | 0 | 0 | Sudan | Northern-Africa | -71.925 | 58.042 | -26.359 | -5.214 | 7.267 |
| SC0061 | PI576429 | Guinea | 0 | 0 | Sudan | Northern-Africa | -31.640 | -11.467 | -8.177 | 0.208 | 23.775 |
| SC0062 | PI534138 | Caudatum | 0 | 0 | Sudan | Northern-Africa | -21.556 | -5.482 | 22.765 | -4.222 | 3.883 |
| SC0063 | PI533912 | Caudatum | 0 | 0 | Sudan | Northern-Africa | -32.143 | 12.067 | 15.048 | -5.634 | 6.157 |
| SC0064 | PI533757 | Caudatum | 0 | 0 | Sudan | Northern-Africa | -65.785 | 58.400 | -12.879 | -1.597 | 4.910 |

Table S4.1. continued

| SC0066 | PI533913 | Guinea-Caudatum | 0 | 0 | Sudan | Northern-Africa | -31.877 | 0.217 | 18.077 | 4.714 | 5.180 |
| --- | --- | --- | --- | --- | --- | --- | --- | --- | --- | --- | --- |
| SC0067 | PI534139 | Guinea-Caudatum | 0 | 0 | Sudan | Northern-Africa | -18.676 | -4.852 | 5.401 | 3.376 | 4.621 |
| SC0068 | PI534140 | Caudatum | 0 | 0 | Kenya | Eastern-Africa | -33.375 | 26.189 | -7.254 | -3.675 | 9.030 |
| SC0069 | PI595725 | Caudatum | 0 | 0 | Kenya | Eastern-Africa | -33.654 | 6.984 | 22.774 | -2.207 | 14.943 |
| SC0072 | PI 153844 | Durra-Caudatum | 0 | 0 | Kenya | Eastern-Africa | -18.119 | 3.493 | 14.906 | 8.345 | -5.946 |
| SC0073 | PI576430 | Caudatum | 0 | 0 | Kenya | Eastern-Africa | -17.166 | -9.222 | 26.848 | 6.770 | -0.700 |
| SC0074 | PI 153846 | Unknown/Other | 0 | 0 | Kenya | Eastern-Africa | 5.621 | 2.638 | 60.793 | 1.006 | -37.144 |
| SC0075 | PI 153852 | Caudatum | 0 | 0 | Kenya | Eastern-Africa | -12.136 | -3.676 | 37.325 | -4.501 | 14.868 |
| SC0077 | PI534141 | Caudatum | 0 | 1 | Kenya | Eastern-Africa | -16.128 | -2.893 | 34.172 | -4.794 | 20.428 |
| SC0078 | PI533914 | Caudatum | 0 | 0 | Kenya | Eastern-Africa | -10.335 | -8.413 | 48.014 | 1.629 | 19.755 |
| SC0079 | PI533915 | Unknown/Other | 0 | 0 | Kenya | Eastern-Africa | -8.361 | -4.294 | 38.248 | 0.293 | 23.686 |
| SC0080 | PI534142 | Caudatum | 0 | 0 | Kenya | Eastern-Africa | -23.070 | -4.383 | 14.168 | 8.978 | -3.663 |
| SC0083 | PI534143 | Caudatum | 0 | 0 | Uganda | Eastern-Africa | -26.299 | -17.758 | 24.545 | 4.353 | 36.875 |
| SC0084 | PI534144 | Caudatum | 0 | 0 | Uganda | Eastern-Africa | -21.348 | -19.304 | 16.675 | 7.397 | 38.302 |
| SC0085 | PI656109 | Caudatum-Bicolor | 0 | 0 | Uganda | Eastern-Africa | -16.528 | 8.899 | 11.440 | 9.172 | -4.455 |
| SC0086 | PI595726 | Durra-Caudatum | 0 | 0 | Kenya | Eastern-Africa | -20.129 | -20.757 | 19.102 | -0.774 | 31.362 |
| SC0087 | PI595727 | Guinea | 0 | 0 | Kenya | Eastern-Africa | -18.741 | -30.546 | 68.954 | -3.547 | 46.159 |
| SC0090 | PI533916 | Guinea | 0 | 0 | Zaire | Middle-Africa | -25.110 | -12.336 | -7.322 | 2.042 | 30.088 |
| SC0091 | PI534145 | Unknown/Other | 0 | 0 | Zimbabwe | Eastern-Africa | -28.512 | -92.700 | -37.129 | -5.399 | 2.393 |
| SC0092 | NA | Durra | 0 | 0 | Ethiopia | Eastern-Africa | 39.863 | -5.153 | 54.771 | -8.628 | -16.171 |
| SC0093 | PI533751 | Caudatum | 0 | 0 | Sudan | Northern-Africa | -29.741 | -23.806 | -7.346 | -6.460 | 31.330 |
| SC0094 | PI533950 | Guinea | 0 | 0 | Sudan | Northern-Africa | -35.414 | -112.916 | -34.702 | -15.331 | 0.251 |
| SC0096 | PI533951 | Caudatum | 0 | 0 | Nigeria | Western-Africa | -62.138 | 46.142 | -26.155 | -3.332 | 11.514 |
| SC0097 | PI533917 | Guinea | 0 | 0 | Nigeria | Western-Africa | -40.050 | -113.154 | -40.396 | -11.920 | -3.546 |
| SC0098 | PI533790 | Guinea | 0 | 0 | Nigeria | Western-Africa | -35.619 | -112.233 | -43.527 | -9.678 | 2.448 |
| SC0099 | PI576383 | Guinea | 0 | 0 | Nigeria | Western-Africa | -40.464 | -93.750 | -44.524 | -10.894 | 2.742 |
| SC0100 | PI533791 | Guinea | 0 | 1 | Nigeria | Western-Africa | -36.252 | -115.038 | -44.839 | -9.995 | 2.157 |
| SC0101 | PI533954 | Guinea-Caudatum | 0 | 0 | S.Africa | Southern-Africa | -18.690 | -12.248 | 77.019 | -1.696 | 10.514 |
| SC0103 | PI533752 | Caudatum | 0 | 0 | S.Africa | Southern-Africa | -60.510 | 35.506 | -21.867 | -0.612 | 29.758 |
| SC0105 | PI533745 | Durra | 0 | 1 | India | India | 81.716 | 15.231 | -22.949 | -24.987 | 24.464 |
| SC0106 | PI 248238 | Bicolor | 0 | NA | Ethiopia | Eastern-Africa | 38.179 | -3.571 | -5.858 | 159.457 | 11.445 |
| SC0108 | PI533792 | Caudatum | 0 | 0 | Ethiopia | Eastern-Africa | -80.001 | 69.036 | -23.884 | -5.871 | -1.171 |

Table S4.1. continued

| SC0109 | PI533793 | Caudatum | 0 | 0 | Ethiopia | Eastern-Africa | -66.795 | 69.983 | -30.769 | 0.493 | -11.493 |
| --- | --- | --- | --- | --- | --- | --- | --- | --- | --- | --- | --- |
| SC0110 | PI533794 | Caudatum | 0 | 0 | Ethiopia | Eastern-Africa | -63.105 | 63.976 | -29.933 | -2.556 | 2.371 |
| SC0111 | PI534146 | Caudatum | 0 | 0 | Ethiopia | Eastern-Africa | -74.245 | 64.784 | -29.527 | -3.257 | 4.827 |
| SC0112 | PI533918 | Caudatum | 0 | 0 | Ethiopia | Eastern-Africa | -68.894 | 62.928 | -35.381 | -3.294 | 4.625 |
| SC0113 | PI576395 | Caudatum | 0 | 0 | Uganda | Eastern-Africa | -58.464 | 41.419 | 0.525 | -3.517 | 5.388 |
| SC0114 | PI533832 | Caudatum | 0 | 0 | Uganda | Eastern-Africa | -73.184 | 58.248 | -24.642 | -3.865 | 12.165 |
| SC0115 | PI533965 | Caudatum-Bicolor | 0 | 0 | Uganda | Eastern-Africa | -61.766 | 38.423 | -20.141 | -1.669 | 29.935 |
| SC0118 | PI533759 | Caudatum | 0 | 0 | Sudan | Northern-Africa | -57.582 | 37.459 | -23.316 | -6.917 | 24.524 |
| SC0119 | PI533973 | Caudatum | 0 | 0 | Zimbabwe | Eastern-Africa | -59.740 | 44.246 | -16.235 | -5.680 | 15.833 |
| SC0120 | PI533760 | Caudatum | 0 | 0 | Sudan | Northern-Africa | -66.834 | 57.479 | -27.046 | -0.535 | 0.110 |
| SC0121 | PI533961 | Caudatum | 0 | 0 | S.Africa | Southern-Africa | -33.843 | 10.493 | 41.583 | -9.426 | 26.894 |
| SC0123 | PI534147 | Durra-Bicolor | 0 | 0 | Ethiopia | Eastern-Africa | 50.903 | 7.824 | 2.754 | 22.708 | -15.477 |
| SC0124 | PI533919 | Durra-Bicolor | 0 | 0 | Ethiopia | Eastern-Africa | 50.037 | 5.792 | -17.543 | 93.340 | -14.098 |
| SC0126 | PI533795 | Durra-Bicolor | 0 | 0 | Ethiopia | Eastern-Africa | -49.719 | 41.562 | -19.305 | -4.490 | 21.798 |
| SC0127 | PI533920 | Durra-Bicolor | 0 | 0 | Ethiopia | Eastern-Africa | 43.668 | -0.653 | -4.178 | 146.112 | 1.995 |
| SC0131 | PI533796 | Durra-Bicolor | 0 | 0 | Ethiopia | Eastern-Africa | 28.816 | 13.886 | -6.662 | 60.504 | -17.355 |
| SC0132 | PI659695 | Durra-Bicolor | 0 | 0 | Ethiopia | Eastern-Africa | 61.901 | 15.361 | -29.831 | 9.167 | -32.471 |
| SC0134 | PI656114 | Durra-Bicolor | 0 | 0 | Ethiopia | Eastern-Africa | 61.890 | 14.319 | -19.098 | 33.557 | -45.098 |
| SC0135 | PI534148 | Durra-Bicolor | 0 | 0 | Ethiopia | Eastern-Africa | 52.991 | 14.095 | -15.096 | 39.754 | -30.746 |
| SC0136 | PI534149 | Durra-Bicolor | 0 | 0 | Ethiopia | Eastern-Africa | 35.944 | 1.216 | -0.431 | 125.428 | 0.409 |
| SC0137 | PI534150 | Durra-Bicolor | 0 | 0 | Ethiopia | Eastern-Africa | 23.629 | 0.265 | 2.067 | 100.455 | 2.825 |
| SC0138 | NA | Durra-Bicolor | 0 | 0 | Ethiopia | Eastern-Africa | 72.751 | 19.340 | -23.682 | 19.278 | -22.883 |
| SC0139 | PI534151 | Durra-Bicolor | 0 | 0 | Ethiopia | Eastern-Africa | 48.405 | 10.731 | -3.392 | 54.797 | -30.158 |
| SC0140 | PI576431 | Durra-Bicolor | 0 | 0 | Ethiopia | Eastern-Africa | 51.137 | 15.592 | -14.213 | 71.633 | -30.047 |
| SC0141 | PI534152 | Durra-Bicolor | 0 | 0 | Ethiopia | Eastern-Africa | 49.026 | 9.950 | -17.419 | 72.857 | -22.501 |
| SC0142 | PI534153 | Durra-Bicolor | 0 | 0 | Ethiopia | Eastern-Africa | 30.943 | 11.058 | 10.257 | 15.200 | -31.829 |
| SC0144 | PI533921 | Durra-Caudatum | 0 | 0 | Ethiopia | Eastern-Africa | 0.887 | 5.543 | 1.664 | -7.370 | 4.919 |
| SC0145 | PI656082 | Bicolor | 0 | 0 | Ethiopia | Eastern-Africa | 31.224 | -15.345 | 0.534 | 127.335 | 33.554 |
| SC0146 | PI534154 | Caudatum | 0 | 0 | Ethiopia | Eastern-Africa | -69.023 | 54.973 | -16.608 | 0.176 | 5.266 |
| SC0147 | PI533922 | Bicolor | 0 | 0 | Ethiopia | Eastern-Africa | 29.049 | -17.529 | -1.575 | 120.819 | 36.362 |
| SC0150 | NA | Durra-Bicolor | 0 | 0 | Ethiopia | Eastern-Africa | 51.811 | 6.529 | -7.871 | 78.417 | -25.644 |
| SC0154 | PI533797 | Durra-Bicolor | 0 | 0 | Ethiopia | Eastern-Africa | 53.427 | 7.598 | -0.307 | 105.300 | -13.238 |

Table S4.1. continued

| SC0155 | PI534155 | Durra-Bicolor | 0 | 0 | Ethiopia | Eastern-Africa | 51.121 | 1.598 | -16.229 | 73.685 | -2.119 |
| --- | --- | --- | --- | --- | --- | --- | --- | --- | --- | --- | --- |
| SC0156 | NA | Bicolor | 0 | 0 | Ethiopia | Eastern-Africa | 43.457 | -9.727 | -6.628 | 90.135 | 32.564 |
| SC0157 | NA | Unknown/Other | 0 | 0 | Ethiopia | Eastern-Africa | 44.217 | 9.048 | 19.828 | 2.617 | -52.043 |
| SC0158 | PI534156 | Durra | NA | 1 | Ethiopia | Eastern-Africa | 73.670 | 3.202 | -20.759 | 47.800 | 30.151 |
| SC0159 | NA | Unknown/Other | 0 | 0 | Ethiopia | Eastern-Africa | 47.469 | -0.042 | -2.521 | 93.826 | 15.652 |
| SC0161 | PI595728 | Durra-Caudatum | 0 | 0 | Ethiopia | Eastern-Africa | 52.913 | -3.105 | -8.520 | 51.287 | 17.750 |
| SC0165 | PI533923 | Caudatum | 0 | 0 | Ethiopia | Eastern-Africa | -43.531 | 31.409 | -11.235 | -3.008 | -0.181 |
| SC0166 | PI533924 | Durra-Bicolor | 0 | 0 | Ethiopia | Eastern-Africa | 41.553 | 0.538 | -9.570 | 155.584 | 8.732 |
| SC0167 | PI533925 | Durra-Bicolor | 0 | 0 | Ethiopia | Eastern-Africa | 42.423 | -0.572 | -5.584 | 157.988 | 8.283 |
| SC0170 | PI534157 | Caudatum | 0 | 0 | Ethiopia | Eastern-Africa | -79.138 | 53.244 | -23.422 | -7.130 | 9.181 |
| SC0171 | PI533798 | Caudatum | 0 | 0 | Ethiopia | Eastern-Africa | -41.049 | 33.447 | 26.311 | -10.486 | -22.103 |
| SC0172 | PI656117 | Caudatum | 0 | 0 | Ethiopia | Eastern-Africa | -74.611 | 66.904 | -27.096 | -5.721 | 0.773 |
| SC0173 | PI533799 | Caudatum | 0 | 0 | Ethiopia | Eastern-Africa | -77.823 | 67.787 | -29.905 | -3.545 | 5.160 |
| SC0175 | PI533800 | Caudatum | 0 | 0 | Ethiopia | Eastern-Africa | -66.321 | 59.760 | -27.357 | -5.932 | -2.957 |
| SC0176 | PI534158 | Caudatum | 0 | 1 | Ethiopia | Eastern-Africa | -68.513 | 58.187 | 1.171 | -6.210 | -8.049 |
| SC0178 | NA | Unknown/Other | 0 | 0 | Ethiopia | Eastern-Africa | 55.943 | 8.519 | -3.707 | 81.895 | -5.444 |
| SC0179 | NA | Unknown/Other | 0 | 0 | Ethiopia | Eastern-Africa | 71.457 | 7.909 | 14.614 | -23.366 | -20.766 |
| SC0180 | NA | Durra | 0 | 0 | Ethiopia | Eastern-Africa | 33.541 | 2.520 | -7.996 | -8.746 | 11.165 |
| SC0181 | PI534159 | Durra-Bicolor | 0 | 0 | Ethiopia | Eastern-Africa | 37.731 | -0.741 | -0.460 | 132.095 | 7.689 |
| SC0182 | PI533801 | Durra-Bicolor | 0 | 0 | Ethiopia | Eastern-Africa | 53.529 | -0.864 | -22.946 | 81.468 | 37.927 |
| SC0183 | PI534160 | Kafir | 0 | 0 | Mexico | Americas | -24.989 | 0.142 | 37.253 | -0.384 | 4.403 |
| SC0184 | PI597958 | Caudatum-Kafir | 0 | 0 | S.Africa | Southern-Africa | -18.895 | -2.019 | 35.098 | -0.473 | 26.585 |
| SC0185 | PI534161 | Caudatum | 0 | 1 | Nigeria | Western-Africa | -45.158 | 18.148 | -20.812 | -4.979 | -0.339 |
| SC0186 | PI533802 | Caudatum-Durra | 0 | 0 | Nigeria | Western-Africa | -24.218 | -55.658 | -27.310 | -2.447 | -31.921 |
| SC0187 | PI533803 | Caudatum-Durra | 0 | 0 | Nigeria | Western-Africa | -17.052 | -45.770 | -21.034 | -2.281 | -32.097 |
| SC0188 | PI533804 | Caudatum-Durra | 0 | 0 | Nigeria | Western-Africa | -21.833 | -42.929 | -17.436 | -1.723 | -30.809 |
| SC0190 | NA | Durra | 0 | 0 | India | India | 66.074 | 2.171 | 14.138 | -29.639 | 1.207 |
| SC0191 | NA | Durra-Bicolor | 0 | 0 | India | India | 67.874 | 11.273 | -16.885 | -17.866 | 3.323 |
| SC0192 | PI576390 | Durra | 0 | 0 | India | India | 75.064 | 17.415 | -14.481 | -26.816 | 6.267 |
| SC0193 | PI533747 | Durra-Bicolor | 0 | 0 | India | India | 97.203 | 23.633 | -29.077 | -26.601 | -3.758 |
| SC0196 | PI597943 | Durra | 0 | 0 | India | India | 74.922 | 10.027 | -24.301 | -21.631 | 20.263 |
| SC0199 | PI533810 | Durra | 1 | 1 | India | India | 108.122 | 25.354 | -25.019 | -31.300 | -2.289 |

Table S4.1. continued

| SC0200 | PI533932 | Durra | 0 | 0 | India | India | 106.236 | 29.234 | -22.477 | -30.684 | -0.404 |
| --- | --- | --- | --- | --- | --- | --- | --- | --- | --- | --- | --- |
| SC0201 | NA | Durra | 1 | 0 | India | India | 88.936 | 11.901 | -30.803 | -33.998 | 29.544 |
| SC0202 | PI533811 | Durra | 0 | 0 | India | India | 90.449 | 12.510 | -20.531 | -37.608 | 28.857 |
| SC0203 | PI533812 | Durra | 0 | 0 | India | India | 99.074 | 14.277 | -33.848 | -38.188 | 31.877 |
| SC0205 | PI533813 | Durra | 0 | 0 | India | India | 96.175 | 18.912 | -38.064 | -36.795 | 32.723 |
| SC0206 | PI533814 | Durra | 0 | 0 | India | India | 97.783 | 12.029 | -38.070 | -39.807 | 37.038 |
| SC0207 | PI533815 | Durra | 0 | 0 | India | India | 97.106 | 14.589 | -32.515 | -38.678 | 33.237 |
| SC0208 | PI533816 | Durra | 0 | 0 | India | India | 96.717 | 14.940 | -42.224 | -40.701 | 36.174 |
| SC0209 | PI533817 | Durra | 0 | 0 | India | India | 90.719 | 22.189 | -18.975 | -26.219 | 1.149 |
| SC0210 | PI533942 | Durra | 0 | 0 | India | India | 91.024 | 11.704 | -30.221 | -34.777 | 31.125 |
| SC0211 | PI533945 | Durra | 0 | 0 | India | India | 103.811 | 23.233 | -37.660 | -33.882 | 8.473 |
| SC0212 | PI533823 | Durra | 0 | 1 | India | India | 95.697 | 13.060 | -32.553 | -35.650 | 31.899 |
| SC0213 | PI576391 | Bicolor | 0 | 0 | India | India | 85.400 | 7.666 | -20.284 | -30.944 | 38.117 |
| SC0214 | PI533750 | Bicolor | 0 | 0 | India | India | 65.019 | 2.991 | -16.881 | -12.338 | 9.898 |
| SC0215 | PI533825 | Durra | 0 | 1 | India | India | 94.430 | 12.521 | -29.750 | -37.691 | 32.503 |
| SC0216 | PI533827 | Durra | 0 | 0 | India | India | 86.399 | 28.891 | -15.632 | -20.568 | -13.249 |
| SC0217 | PI533926 | Durra | 0 | 1 | India | India | 104.468 | 23.108 | -33.889 | -29.934 | -3.727 |
| SC0218 | PI534162 | Durra | 0 | 0 | China | Asia | 76.462 | 13.962 | -31.215 | -27.976 | 16.442 |
| SC0220 | PI533805 | Durra | 0 | 0 | India | India | 104.000 | 23.169 | -42.329 | -32.487 | 11.978 |
| SC0221 | PI533806 | Durra | 0 | 1 | India | India | 102.212 | 12.050 | -39.423 | -38.262 | 34.122 |
| SC0222 | PI533931 | Durra | 0 | 0 | India | India | 78.008 | 12.734 | -29.159 | -22.866 | 16.073 |
| SC0223 | PI533807 | Caudatum | 0 | 0 | Nigeria | Western-Africa | -58.225 | 39.553 | -20.659 | 1.173 | 9.663 |
| SC0224 | PI533927 | Bicolor | 0 | 0 | Ethiopia | Eastern-Africa | 22.836 | -12.579 | -5.270 | 103.190 | 28.796 |
| SC0226 | PI533828 | Durra-Bicolor | 0 | 0 | Ethiopia | Eastern-Africa | 55.789 | 11.954 | -18.989 | 4.932 | -31.506 |
| SC0227 | PI533829 | Durra | 0 | 0 | Ethiopia | Eastern-Africa | 64.216 | 11.699 | -24.296 | -4.109 | -18.610 |
| SC0228 | PI533756 | Caudatum | 0 | 0 | Ethiopia | Eastern-Africa | -59.600 | 57.472 | -14.887 | -6.639 | -5.003 |
| SC0229 | PI533808 | Caudatum | 0 | 0 | Nigeria | Western-Africa | -49.157 | -3.519 | -20.743 | -10.731 | -15.790 |
| SC0230 | PI533809 | Durra | 0 | 0 | India | India | 84.864 | 7.684 | -33.261 | -35.861 | 31.093 |
| SC0231 | PI533935 | Durra | 0 | 0 | India | India | 90.017 | 27.987 | -33.935 | -22.098 | -11.364 |
| SC0233 | PI533746 | Durra | 0 | 0 | India | India | 97.714 | 17.289 | -30.966 | -33.828 | 27.137 |
| SC0235 | NA | Guinea-Bicolor | 0 | 0 | Ethiopia | Eastern-Africa | 33.663 | 1.003 | -2.520 | 118.450 | 3.938 |
| SC0236 | NA | Durra | 0 | 0 | Unknown | Unknown | 50.709 | -3.194 | 27.867 | -9.875 | -7.546 |

Table S4.1. continued

| SC0237 | PI533834 | Caudatum | 0 | NA | Sudan | Northern-Africa | -73.789 | 61.580 | -25.757 | -6.005 | 12.979 |
| --- | --- | --- | --- | --- | --- | --- | --- | --- | --- | --- | --- |
| SC0239 | PI533837 | Caudatum | 0 | NA | Sudan | Northern-Africa | -61.684 | 45.621 | -22.291 | -2.059 | -7.553 |
| SC0240 | PI533842 | Durra | NA | NA | India | India | 102.230 | 15.937 | -33.404 | -24.165 | 14.095 |
| SC0241 | PI533843 | Guinea | 0 | NA | India | India | 28.080 | -12.886 | 39.344 | -10.074 | 40.666 |
| SC0242 | PI533844 | Guinea | 1 | 1 | Nepal | Asia | -3.462 | -31.246 | 65.239 | -9.831 | 86.529 |
| SC0243 | PI533845 | Guinea | 0 | 1 | Nepal | Asia | 20.564 | -20.181 | 41.039 | -17.018 | 56.841 |
| SC0244 | PI533847 | Guinea | 0 | 0 | India | India | -2.133 | -23.354 | 60.331 | -9.113 | 69.647 |
| SC0245 | PI534011 | Durra | 0 | 0 | India | India | 74.479 | 5.474 | -16.428 | -30.937 | 42.337 |
| SC0247 | NA | Guinea | 0 | 0 | India | India | 2.668 | -29.720 | 51.855 | -10.779 | 74.428 |
| SC0248 | PI533853 | Guinea | 0 | 0 | India | India | 9.031 | -27.908 | 46.068 | -11.895 | 82.183 |
| SC0249 | NA | Guinea | 0 | 0 | India | India | -16.226 | 15.073 | 31.940 | -1.697 | 27.033 |
| SC0250 | PI533764 | Guinea | 0 | 0 | India | India | 1.592 | -28.430 | 57.340 | -11.257 | 82.478 |
| SC0252 | PI533848 | Guinea | 0 | 1 | India | India | 1.229 | -28.863 | 61.662 | -11.835 | 76.620 |
| SC0253 | PI533765 | Guinea | 0 | 0 | India | India | 6.878 | -27.157 | 52.482 | -10.440 | 83.994 |
| SC0254 | PI533854 | Guinea | 0 | 1 | India | India | 11.326 | -24.618 | 45.275 | -9.003 | 80.467 |
| SC0256 | PI533818 | Guinea | 0 | 1 | Nigeria | Western-Africa | -39.643 | -115.711 | -43.720 | -12.963 | 4.040 |
| SC0257 | PI533819 | Guinea | 0 | 1 | Zimbabwe | Eastern-Africa | -23.761 | -64.104 | 4.585 | -2.019 | 8.889 |
| SC0258 | PI533749 | Guinea | 0 | 1 | Africa | Eastern-Africa | -21.361 | -28.327 | 76.150 | -3.292 | 64.592 |
| SC0259 | PI576334 | Guinea | 0 | 0 | Sudan | Northern-Africa | -28.100 | 13.602 | 59.703 | -11.940 | -8.805 |
| SC0260 | NA | Guinea | NA | NA | Mali | Western-Africa | -18.931 | -59.921 | 6.638 | -9.956 | 3.263 |
| SC0261 | PI533841 | Guinea | 0 | 0 | Nigeria | Western-Africa | -33.030 | -62.575 | -20.282 | -7.901 | 11.960 |
| SC0262 | PI534002 | Guinea | 0 | 0 | Mali | Western-Africa | -31.336 | -35.373 | -18.357 | -8.862 | 13.798 |
| SC0263 | NA | Guinea | 0 | 0 | Mali | Western-Africa | -23.852 | -74.553 | -17.640 | -10.372 | 7.854 |
| SC0265 | PI533766 | Guinea | 0 | 0 | BurkinaFaso | Western-Africa | -33.049 | -81.220 | -28.039 | -9.718 | 12.065 |
| SC0266 | NA | Guinea | 0 | 0 | BurkinaFaso | Western-Africa | -16.445 | -68.575 | -5.615 | -9.340 | -2.728 |
| SC0267 | PI533836 | Guinea | 0 | 0 | Sudan | Northern-Africa | -29.444 | -48.595 | -8.847 | -6.264 | 13.487 |
| SC0268 | PI533768 | Guinea | 0 | 0 | Sudan | Northern-Africa | -40.695 | 15.947 | -14.381 | -6.371 | 23.721 |
| SC0269 | PI534058 | Guinea | 0 | 0 | Nigeria | Western-Africa | -31.401 | -106.774 | -37.087 | -12.583 | -4.311 |
| SC0270 | PI534059 | Guinea | 0 | 0 | Nigeria | Western-Africa | -30.981 | -101.895 | -39.488 | -12.411 | 0.407 |
| SC0271 | PI533772 | Guinea | 0 | 0 | Nigeria | Western-Africa | -19.728 | -57.104 | -23.992 | -7.916 | -12.349 |
| SC0272 | PI533773 | Guinea | 0 | 0 | Nigeria | Western-Africa | -29.033 | -100.840 | -33.758 | -7.319 | -18.568 |
| SC0273 | PI534062 | Guinea | 0 | 0 | Nigeria | Western-Africa | 33.076 | -2.189 | -1.069 | 106.204 | 1.686 |

Table S4.1. continued

| SC0275 | PI534065 | Guinea | 0 | 0 | Nigeria | Western-Africa | -34.496 | -104.178 | -43.364 | -12.801 | 0.620 |
| --- | --- | --- | --- | --- | --- | --- | --- | --- | --- | --- | --- |
| SC0276 | NA | Guinea | 0 | 0 | Nigeria | Western-Africa | -24.426 | -12.955 | -5.538 | -2.166 | -23.551 |
| SC0277 | PI534067 | Guinea | 0 | 0 | Nigeria | Western-Africa | -36.921 | -107.135 | -35.138 | -13.285 | 1.208 |
| SC0278 | PI533763 | Guinea | 0 | 0 | Nigeria | Western-Africa | -28.055 | -71.628 | -23.349 | -5.625 | 10.982 |
| SC0279 | PI534070 | Guinea | 0 | 0 | Nigeria | Western-Africa | -39.065 | -113.730 | -42.245 | -12.009 | 0.180 |
| SC0280 | PI534085 | Guinea | 0 | 0 | Nigeria | Western-Africa | -35.131 | -103.116 | -26.968 | -11.777 | -3.596 |
| SC0281 | PI533786 | Guinea | 0 | 0 | Nigeria | Western-Africa | -12.653 | -45.478 | 17.487 | 6.123 | 20.596 |
| SC0282 | PI533784 | Guinea | 0 | 0 | Nigeria | Western-Africa | -34.839 | -98.082 | -43.122 | -11.461 | 1.042 |
| SC0283 | PI533869 | Guinea | 0 | 0 | Tanzania | Eastern-Africa | -16.013 | -22.988 | 72.473 | -5.618 | 43.412 |
| SC0284 | PI533767 | Guinea | 0 | 0 | BurkinaFaso | Western-Africa | -38.015 | -105.629 | -35.376 | -10.252 | 1.910 |
| SC0285 | PI533862 | Guinea | 0 | 0 | Chad | Middle-Africa | -38.590 | -111.778 | -42.777 | -13.931 | -0.460 |
| SC0287 | NA | Guinea | 0 | 0 | Nigeria | Western-Africa | -38.320 | -110.563 | -40.850 | -13.201 | 0.693 |
| SC0289 | PI533780 | Guinea | 0 | 0 | Nigeria | Western-Africa | -32.358 | -101.725 | -36.821 | -11.041 | -1.804 |
| SC0290 | PI533883 | Guinea | 0 | 0 | Nigeria | Western-Africa | -31.226 | -110.170 | -48.605 | -6.026 | -16.675 |
| SC0291 | PI533888 | Guinea | 0 | 0 | Nigeria | Western-Africa | -24.023 | -59.683 | 33.719 | -8.955 | 36.379 |
| SC0292 | PI533782 | Guinea | 0 | 0 | Nigeria | Western-Africa | -35.899 | -103.604 | -29.451 | -11.816 | -3.992 |
| SC0293 | PI533783 | Guinea | 0 | 0 | Nigeria | Western-Africa | -35.955 | -100.593 | -44.804 | -10.119 | -2.011 |
| SC0295 | PI656093 | Guinea | 0 | 1 | Nigeria | Western-Africa | -35.099 | -109.903 | -32.839 | -11.990 | -0.911 |
| SC0296 | PI533885 | Guinea | 0 | 1 | Nigeria | Western-Africa | -36.086 | -90.974 | -32.721 | -12.004 | 1.544 |
| SC0297 | NA | Guinea | 0 | 1 | Nigeria | Western-Africa | -34.893 | -110.195 | -39.061 | -14.263 | 1.351 |
| SC0298 | PI533887 | Guinea | 0 | 1 | Nigeria | Western-Africa | -23.121 | -70.061 | -33.089 | -9.709 | 0.466 |
| SC0299 | PI533785 | Guinea | 0 | 0 | Nigeria | Western-Africa | -26.920 | -89.382 | -31.248 | -10.602 | 0.191 |
| SC0300 | PI534095 | Guinea | 0 | 0 | Nigeria | Western-Africa | -36.880 | -90.417 | -34.160 | -10.375 | 4.123 |
| SC0301 | PI656094 | Guinea | 0 | 0 | Mali | Western-Africa | -28.128 | -84.831 | -29.663 | -11.856 | 7.242 |
| SC0303 | PI533839 | Guinea | 0 | 1 | Nigeria | Western-Africa | -3.872 | -54.520 | 2.459 | 20.688 | 19.052 |
| SC0305 | PI534037 | Durra | 0 | 0 | Chad | Middle-Africa | -14.266 | -43.688 | 15.896 | 18.400 | -0.135 |
| SC0306 | PI533958 | Bicolor | 0 | 0 | USA | Americas | 33.452 | -16.303 | -8.176 | 146.541 | 38.836 |
| SC0308 | PI533820 | Bicolor | 0 | 0 | Malawi | Eastern-Africa | -18.572 | -33.815 | 68.072 | -1.758 | 58.628 |
| SC0309 | PI533754 | Bicolor | 0 | 0 | Sudan | Northern-Africa | 27.417 | -4.524 | -16.824 | 4.820 | 8.914 |
| SC0311 | PI533753 | Bicolor | 0 | 1 | Sudan | Northern-Africa | 25.421 | -4.351 | -15.363 | 4.137 | 9.624 |
| SC0314 | NA | Bicolor-Guinea | 0 | 0 | India | India | 2.451 | -12.288 | 85.568 | -1.251 | -5.036 |
| SC0315 | PI533851 | Guinea-Bicolor | 0 | 1 | India | India | 15.066 | -10.884 | 49.907 | -5.285 | 52.563 |

Table S4.1. continued

| SC0317 | PI533855 | Guinea-Bicolor | 0 | 1 | India | India | 14.343 | -13.009 | 61.705 | -3.181 | 44.279 |
| --- | --- | --- | --- | --- | --- | --- | --- | --- | --- | --- | --- |
| SC0319 | PI533833 | Caudatum | 0 | 0 | Uganda | Eastern-Africa | -58.479 | 65.064 | -24.321 | 4.711 | -16.924 |
| SC0320 | PI533863 | Kafir | 0 | 0 | Chad | Middle-Africa | -37.024 | 17.674 | -15.419 | -1.537 | 11.011 |
| SC0322 | PI533821 | Caudatum | 0 | 0 | Tanzania | Eastern-Africa | -30.018 | -8.516 | 28.289 | 3.875 | 18.895 |
| SC0323 | PI576399 | Caudatum | 0 | 0 | Sudan | Northern-Africa | -38.712 | -4.987 | 24.859 | -0.362 | 11.736 |
| SC0324 | PI576396 | Caudatum | 0 | 0 | Uganda | Eastern-Africa | -67.872 | 50.352 | -11.560 | -6.321 | 9.348 |
| SC0325 | PI533957 | Caudatum | 0 | 0 | USA | Americas | -26.446 | -5.363 | 8.350 | 15.036 | 19.228 |
| SC0328 | PI534112 | Caudatum | 0 | 0 | Uganda | Eastern-Africa | -72.213 | 62.059 | -15.733 | -5.451 | 9.784 |
| SC0329 | PI533838 | Caudatum | 0 | 0 | Nigeria | Western-Africa | -43.150 | 9.553 | -9.857 | -4.852 | -10.842 |
| SC0330 | PI534106 | Caudatum | 0 | 0 | Uganda | Eastern-Africa | -64.690 | 49.605 | -12.179 | -3.699 | 11.925 |
| SC0331 | PI533824 | Durra-Caudatum | 0 | 0 | Nigeria | Western-Africa | 22.836 | -9.579 | -2.388 | 112.312 | 28.265 |
| SC0333 | PI533761 | Caudatum | 0 | 0 | Ethiopia | Eastern-Africa | -39.411 | 20.523 | -19.800 | -5.871 | 19.840 |
| SC0334 | PI533986 | Caudatum | 0 | 0 | Sudan | Northern-Africa | -46.404 | 24.370 | -19.391 | -2.598 | 15.999 |
| SC0335 | PI533865 | Caudatum | 0 | 0 | Sudan | Northern-Africa | -21.619 | 3.165 | -13.468 | -5.248 | 16.773 |
| SC0336 | PI533995 | Caudatum | 0 | 0 | Sudan | Northern-Africa | -12.257 | 7.731 | 12.806 | -4.652 | -8.379 |
| SC0337 | PI533868 | Caudatum | 0 | 0 | Sudan | Northern-Africa | -36.153 | 11.856 | -9.601 | -5.487 | 23.305 |
| SC0338 | PI534044 | Caudatum | 0 | 0 | Sudan | Northern-Africa | -59.501 | 36.810 | -17.727 | -4.098 | 24.493 |
| SC0339 | PI576412 | Caudatum | 0 | 0 | Sudan | Northern-Africa | -22.966 | 4.258 | -12.797 | 2.569 | 5.836 |
| SC0340 | PI533867 | Caudatum | 0 | 0 | Sudan | Northern-Africa | -21.994 | 10.233 | -21.673 | 0.515 | 9.961 |
| SC0342 | PI534064 | Caudatum | 0 | 0 | Nigeria | Western-Africa | -34.800 | -65.234 | -27.989 | -7.778 | 8.983 |
| SC0343 | NA | Caudatum | 0 | 0 | Nigeria | Western-Africa | -37.066 | 21.011 | 5.924 | -6.543 | 13.555 |
| SC0344 | PI533774 | Caudatum | 0 | 0 | Nigeria | Western-Africa | -22.584 | -17.432 | -17.647 | -6.037 | 9.297 |
| SC0345 | PI534073 | Caudatum | 0 | 0 | Nigeria | Western-Africa | -13.746 | -11.084 | -15.738 | -5.801 | 6.909 |
| SC0347 | PI534074 | Caudatum | 0 | 0 | Nigeria | Western-Africa | -26.117 | -77.871 | -31.001 | -12.660 | -18.145 |
| SC0348 | PI534075 | Caudatum | 0 | 0 | Nigeria | Western-Africa | -22.119 | -77.241 | -29.020 | -10.211 | -15.046 |
| SC0349 | PI595712 | Caudatum | 0 | 0 | Nigeria | Western-Africa | -19.277 | -7.790 | -13.298 | -3.651 | 7.089 |
| SC0350 | PI576357 | Caudatum | 0 | 0 | Nigeria | Western-Africa | -37.347 | -16.157 | -22.796 | -4.618 | 16.404 |
| SC0351 | PI534060 | Caudatum | 0 | 0 | Nigeria | Western-Africa | -32.548 | -96.471 | -32.725 | -8.541 | -1.570 |
| SC0352 | PI534042 | Caudatum | 0 | 0 | Sudan | Northern-Africa | -19.564 | 9.140 | -1.768 | -5.788 | 12.496 |
| SC0353 | PI533777 | Guinea-Caudatum | 0 | 0 | Sudan | Northern-Africa | -19.877 | -48.890 | -28.963 | 0.624 | -28.255 |
| SC0354 | PI533890 | Caudatum | 0 | 0 | Nigeria | Western-Africa | -34.061 | -94.113 | -28.832 | -12.949 | -1.964 |
| SC0356 | PI533895 | Caudatum | 0 | 0 | Nigeria | Western-Africa | -37.264 | -61.616 | -35.620 | -11.697 | 7.700 |

Table S4.1. continued

| SC0358 | PI533879 | Caudatum | 0 | 0 | Nigeria | Western-Africa | -19.116 | -72.581 | -33.310 | -9.070 | -30.111 |
| --- | --- | --- | --- | --- | --- | --- | --- | --- | --- | --- | --- |
| SC0362 | PI533775 | Caudatum | 0 | 0 | Nigeria | Western-Africa | -28.832 | -63.822 | -21.053 | -11.003 | -31.237 |
| SC0366 | PI533771 | Caudatum | 0 | 0 | Nigeria | Western-Africa | -21.229 | -69.973 | -36.294 | -6.051 | -33.468 |
| SC0367 | PI533872 | Caudatum | 0 | 0 | Nigeria | Western-Africa | -21.796 | -67.102 | -28.525 | -6.694 | -45.128 |
| SC0368 | PI533873 | Caudatum | 0 | 0 | Nigeria | Western-Africa | -29.042 | -68.497 | -29.396 | -8.462 | -44.179 |
| SC0369 | PI533874 | Caudatum | 0 | 0 | Nigeria | Western-Africa | -19.105 | -71.688 | -30.856 | -6.891 | -32.441 |
| SC0370 | PI533776 | Caudatum | 0 | 0 | Nigeria | Western-Africa | -29.150 | -66.082 | -35.906 | -13.246 | -28.983 |
| SC0371 | NA | Caudatum | 0 | 0 | Nigeria | Western-Africa | -30.361 | -61.703 | -33.444 | -12.916 | -32.999 |
| SC0372 | PI533878 | Caudatum | 0 | 0 | Nigeria | Western-Africa | -17.814 | -65.275 | -25.664 | -2.989 | -68.504 |
| SC0373 | PI656095 | Caudatum | 0 | 0 | Nigeria | Western-Africa | -39.304 | -54.987 | -33.116 | -12.844 | -32.369 |
| SC0374 | PI533778 | Caudatum | 0 | 0 | Nigeria | Western-Africa | -38.380 | -82.298 | -40.703 | -10.749 | -20.634 |
| SC0377 | PI534082 | Caudatum | 0 | 0 | Nigeria | Western-Africa | -38.630 | -90.203 | -29.754 | -10.864 | -18.676 |
| SC0380 | PI533781 | Caudatum | 0 | 0 | Nigeria | Western-Africa | -21.716 | -39.424 | -33.500 | 0.143 | -22.383 |
| SC0382 | PI534088 | Caudatum | 0 | 0 | Nigeria | Western-Africa | 32.272 | -21.896 | -30.948 | -14.727 | -25.724 |
| SC0384 | PI534089 | Caudatum | 0 | 0 | Nigeria | Western-Africa | -24.949 | -81.301 | -37.374 | -8.786 | -32.847 |
| SC0386 | PI656119 | Caudatum | 0 | 0 | Nigeria | Western-Africa | -22.209 | -76.574 | -13.478 | -7.671 | -37.116 |
| SC0387 | PI533891 | Caudatum | 0 | 0 | Nigeria | Western-Africa | -32.138 | -76.050 | -22.654 | -12.421 | -23.840 |
| SC0388 | PI533893 | Caudatum | 0 | 0 | Nigeria | Western-Africa | -68.518 | 62.235 | -27.891 | -0.482 | -2.444 |
| SC0389 | PI533894 | Caudatum | 0 | 0 | Nigeria | Western-Africa | -30.837 | -65.033 | -28.349 | -14.179 | -27.775 |
| SC0391 | PI656096 | Caudatum | 0 | 0 | Egypt | Northern-Africa | -33.539 | -58.942 | -34.043 | -13.376 | -35.746 |
| SC0392 | PI533770 | Caudatum | 0 | 0 | Nigeria | Western-Africa | -18.545 | -63.627 | -34.669 | -3.040 | -48.323 |
| SC0393 | PI534066 | Caudatum | 0 | 0 | Nigeria | Western-Africa | -51.177 | 15.948 | -20.156 | -8.435 | -14.753 |
| SC0394 | PI533880 | Caudatum | 0 | 0 | Nigeria | Western-Africa | -29.424 | -67.582 | -28.278 | -14.964 | -34.469 |
| SC0396 | PI533877 | Caudatum | 0 | 0 | Nigeria | Western-Africa | -29.398 | -64.854 | -36.381 | -13.431 | -27.427 |
| SC0397 | PI534076 | Caudatum | 0 | 0 | Nigeria | Western-Africa | -24.114 | -43.728 | -20.418 | -11.331 | -31.132 |
| SC0398 | PI533881 | Caudatum | 0 | 0 | Nigeria | Western-Africa | -31.033 | -65.303 | -28.531 | -12.494 | -36.246 |
| SC0399 | PI533882 | Caudatum | 0 | 0 | Nigeria | Western-Africa | -16.584 | -50.589 | -31.533 | -3.468 | -40.846 |
| SC0400 | NA | Caudatum | 0 | 1 | Nigeria | Western-Africa | -20.732 | -62.441 | 7.128 | -15.435 | -17.030 |
| SC0401 | PI533896 | Caudatum | 0 | 0 | Nigeria | Western-Africa | -29.893 | -65.558 | -30.170 | -10.714 | -22.398 |
| SC0402 | PI533897 | Caudatum | 0 | 0 | Nigeria | Western-Africa | -2.250 | -30.568 | -26.159 | -19.400 | -8.246 |
| SC0403 | PI533898 | Caudatum | 0 | 0 | Nigeria | Western-Africa | -34.877 | -55.224 | -30.709 | -9.690 | -32.697 |
| SC0405 | PI533835 | Caudatum | 0 | 0 | Sudan | Northern-Africa | -55.382 | 52.671 | -24.198 | 3.797 | -9.711 |

Table S4.1. continued

| SC0406 | PI597953 | Guinea | 0 | 0 | Nigeria | Western-Africa | -27.621 | -48.900 | -33.259 | -0.437 | -30.030 |
| --- | --- | --- | --- | --- | --- | --- | --- | --- | --- | --- | --- |
| SC0407 | PI533779 | Guinea-Caudatum | 0 | 0 | Nigeria | Western-Africa | -3.631 | -43.409 | -25.845 | 5.378 | -38.193 |
| SC0408 | PI533884 | Caudatum | 0 | 0 | Nigeria | Western-Africa | -14.202 | -63.657 | -20.285 | -2.989 | -26.966 |
| SC0409 | NA | Caudatum-Guinea | 0 | 0 | Nigeria | Western-Africa | -16.108 | -39.625 | -18.551 | 2.718 | -25.280 |
| SC0411 | PI533866 | Caudatum-Bicolor | 0 | 0 | Sudan | Northern-Africa | -10.305 | -5.305 | -4.620 | -2.201 | -21.964 |
| SC0412 | NA | Caudatum-Bicolor | 0 | 0 | Sudan | Northern-Africa | 17.913 | 12.365 | -12.382 | 11.530 | -15.200 |
| SC0413 | PI534079 | Caudatum-Bicolor | 0 | 0 | Nigeria | Western-Africa | -35.196 | -14.343 | -24.084 | 0.832 | -19.102 |
| SC0414 | PI533831 | Caudatum | 0 | 1 | Sudan | Northern-Africa | -29.422 | 32.549 | -14.754 | 4.075 | -3.934 |
| SC0417 | PI533861 | Caudatum | 0 | 1 | Senegal | Western-Africa | 46.989 | 15.852 | -22.489 | 4.932 | -42.059 |
| SC0418 | PI533822 | Caudatum | 0 | 0 | Tanzania | Eastern-Africa | -41.747 | 21.109 | 5.853 | 3.403 | 8.529 |
| SC0420 | PI533769 | Caudatum | 0 | 0 | Sudan | Northern-Africa | -34.859 | 9.927 | -7.920 | -2.935 | -17.779 |
| SC0422 | PI576421 | Caudatum | 0 | 0 | Uganda | Eastern-Africa | -35.069 | 4.650 | 11.370 | 5.677 | 7.759 |
| SC0423 | PI533758 | Caudatum | 0 | 0 | Sudan | Northern-Africa | -68.727 | 54.168 | -18.503 | -4.129 | 4.167 |
| SC0424 | PI533901 | Caudatum | 0 | 0 | Japan | Asia | -36.231 | 26.210 | -6.740 | -4.302 | -1.620 |
| SC0425 | PI533762 | Caudatum | 0 | 0 | Sudan | Northern-Africa | -33.388 | 21.699 | -20.983 | 1.366 | 3.101 |
| SC0426 | PI533900 | Caudatum | 0 | 0 | Japan | Asia | 3.429 | -25.314 | -23.894 | 8.211 | -46.465 |
| SC0427 | PI533899 | Caudatum | 0 | 0 | Nigeria | Western-Africa | 18.202 | -5.470 | -17.974 | 10.397 | -18.949 |
| SC0428 | PI534057 | Caudatum | 0 | 0 | Nigeria | Western-Africa | 5.148 | 7.555 | 14.495 | 5.863 | 9.198 |
| SC0430 | PI576398 | Durra | 0 | 1 | Ethiopia | Eastern-Africa | 43.228 | -11.179 | -5.405 | 128.904 | 34.085 |
| SC0431 | PI533846 | Durra | 0 | 0 | India | India | 91.008 | 13.743 | -12.879 | -32.291 | 17.191 |
| SC0432 | PI533941 | Durra | 0 | 0 | Tanzania | Eastern-Africa | -9.037 | -25.002 | 34.674 | -9.413 | 11.285 |
| SC0435 | PI576331 | Durra | 0 | 1 | India | India | 21.235 | -16.821 | 47.993 | -10.150 | 71.660 |
| SC0436 | NA | Durra | 0 | 1 | India | India | 84.569 | 9.887 | -19.885 | -31.858 | 27.101 |
| SC0437 | PI533947 | Durra | 0 | 1 | India | India | 86.449 | 12.126 | -25.029 | -30.914 | 26.324 |
| SC0438 | NA | Durra | 0 | 0 | Ethiopia | Eastern-Africa | 37.305 | -7.669 | 62.475 | -9.357 | -14.367 |
| SC0441 | PI534009 | Durra | 0 | 0 | India | India | 83.816 | 8.649 | -16.747 | -34.223 | 37.265 |
| SC0442 | PI534010 | Durra | 0 | 1 | India | India | 89.760 | 8.408 | -29.101 | -34.709 | 35.539 |
| SC0445 | NA | Durra | 0 | 1 | India | India | 12.375 | -18.313 | 48.265 | -14.516 | 79.939 |
| SC0448 | NA | Durra | 1 | 1 | India | India | 97.671 | 16.640 | -33.299 | -36.720 | 27.761 |
| SC0449 | PI597950 | Durra | 0 | 1 | India | India | 21.712 | -9.572 | 48.369 | -10.325 | 61.930 |
| SC0450 | PI533852 | Durra | 0 | 0 | India | India | 75.265 | 8.025 | -18.133 | -19.608 | 16.416 |
| SC0451 | PI533934 | Durra | 0 | 0 | India | India | -60.084 | 48.860 | -29.830 | 0.407 | 8.739 |

Table S4.1. continued

| SC0452 | PI533944 | Durra | 0 | 0 | India | India | 107.257 | 22.121 | -36.769 | -29.911 | 9.947 |
| --- | --- | --- | --- | --- | --- | --- | --- | --- | --- | --- | --- |
| SC0454 | PI576362 | Durra | 0 | 0 | India | India | 89.552 | 8.491 | -35.978 | -35.066 | 41.095 |
| SC0455 | PI534013 | Durra | 0 | 0 | India | India | 85.428 | 14.175 | -22.128 | -37.281 | 27.562 |
| SC0456 | NA | Durra | 0 | 0 | India | India | 80.951 | 11.338 | -7.592 | -32.221 | 18.490 |
| SC0457 | PI533849 | Durra-Bicolor | 0 | 0 | India | India | 81.213 | 16.698 | -0.653 | -21.929 | 9.486 |
| SC0458 | NA | Durra | 0 | 0 | India | India | 75.985 | 7.972 | 0.679 | -29.913 | 25.761 |
| SC0459 | PI533850 | Durra | 0 | 0 | India | India | 97.015 | 19.460 | -12.946 | -32.354 | 11.811 |
| SC0460 | PI534017 | Durra | 0 | 0 | India | India | 84.248 | 15.693 | -23.095 | -25.160 | 20.860 |
| SC0462 | PI534018 | Durra | 0 | 0 | India | India | 87.877 | 12.263 | -24.285 | -32.156 | 21.117 |
| SC0463 | NA | Durra | 0 | 0 | India | India | 88.231 | 13.549 | 0.918 | -34.370 | 10.995 |
| SC0464 | PI534031 | Durra | 0 | 0 | India | India | 83.710 | 9.368 | -17.168 | -36.778 | 24.587 |
| SC0465 | PI533997 | Guinea-Durra | 0 | 0 | Arabia | Arabia/MiddleEast | -15.341 | -25.221 | 37.379 | 2.151 | 16.328 |
| SC0466 | PI533981 | Durra-Bicolor | 0 | 0 | India | India | 9.054 | -5.481 | 68.079 | -8.168 | -24.077 |
| SC0467 | PI533943 | Durra-Bicolor | 0 | 0 | India | India | 83.899 | 15.326 | 7.777 | -27.581 | 0.724 |
| SC0468 | PI533946 | Durra-Bicolor | 0 | 0 | India | India | 64.506 | 13.343 | 33.582 | -13.701 | 4.506 |
| SC0470 | NA | Durra | 0 | 0 | India | India | 83.597 | 14.103 | 17.566 | -23.361 | -13.907 |
| SC0471 | PI534014 | Durra | 0 | 0 | India | India | 67.295 | 14.411 | -25.453 | -28.273 | 17.716 |
| SC0472 | PI534016 | Durra | 0 | 0 | India | India | 105.476 | 24.748 | -31.084 | -28.655 | 12.942 |
| SC0473 | PI534028 | Durra | 0 | 1 | India | India | 96.431 | 26.436 | -18.619 | -30.481 | -4.933 |
| SC0475 | PI534022 | Durra | 0 | 1 | India | India | 105.711 | 23.811 | -27.829 | -32.596 | 9.225 |
| SC0477 | PI534026 | Durra | 1 | 1 | India | India | 107.361 | 20.568 | -24.043 | -33.570 | 11.193 |
| SC0479 | NA | Durra | 1 | 1 | India | India | 100.331 | 29.733 | -24.401 | -32.889 | 2.917 |
| SC0480 | PI656097 | Durra | 0 | 1 | India | India | 111.528 | 29.432 | -33.952 | -34.221 | 7.542 |
| SC0482 | PI533859 | Durra | 0 | 1 | India | India | 113.682 | 29.067 | -30.322 | -34.480 | 3.234 |
| SC0483 | PI534034 | Durra | 1 | 1 | India | India | 110.708 | 32.518 | -24.460 | -32.170 | -5.809 |
| SC0484 | PI534036 | Durra | 0 | 1 | India | India | 94.976 | 18.996 | -30.476 | -1.450 | 7.848 |
| SC0485 | NA | Durra | 0 | NA | India | India | 62.900 | 5.953 | 36.873 | -19.759 | -4.783 |
| SC0489 | PI533856 | Durra | 1 | 1 | India | India | 110.026 | 30.565 | -23.895 | -32.893 | -8.663 |
| SC0490 | PI534024 | Durra | 0 | 0 | India | India | 96.242 | 18.550 | -27.491 | 2.356 | 2.812 |
| SC0491 | PI534025 | Durra | 1 | 1 | India | India | 111.713 | 25.529 | -28.460 | -33.762 | -2.463 |
| SC0492 | PI534027 | Durra | 0 | 1 | India | India | 108.891 | 30.440 | -25.428 | -31.434 | -1.789 |
| SC0493 | PI533857 | Durra | 0 | 1 | India | India | 113.427 | 31.293 | -30.622 | -31.329 | -2.285 |

Table S4.1. continued

| SC0494 | PI534030 | Durra | 0 | 0 | India | India | 109.850 | 31.801 | -24.551 | -32.621 | -5.503 |
| --- | --- | --- | --- | --- | --- | --- | --- | --- | --- | --- | --- |
| SC0496 | PI534032 | Durra | 0 | 1 | India | India | 107.338 | 29.891 | -19.823 | -33.732 | -0.890 |
| SC0497 | PI534033 | Durra | 0 | 1 | India | India | 106.381 | 28.507 | -22.051 | -29.063 | -11.624 |
| SC0498 | PI656099 | Durra | 0 | 1 | India | India | 117.228 | 35.018 | -29.654 | -33.294 | -5.418 |
| SC0499 | PI533858 | Durra | 1 | 1 | India | India | 110.242 | 24.052 | -29.650 | -35.210 | 6.850 |
| SC0500 | PI656100 | Durra | 0 | 1 | India | India | 107.276 | 28.454 | -23.043 | -29.026 | -10.566 |
| SC0501 | PI533860 | Caudatum | 0 | 0 | India | India | -5.904 | 34.948 | -26.757 | -3.764 | -9.437 |
| SC0502 | PI533996 | Caudatum | 0 | 0 | Sudan | Northern-Africa | -23.753 | 19.528 | -17.129 | -8.109 | 16.706 |
| SC0504 | PI533864 | Caudatum | 0 | 0 | Sudan | Northern-Africa | -45.094 | 29.300 | -16.632 | -0.193 | 13.719 |
| SC0505 | NA | Unknown/Other | 0 | 0 | Ethiopia | Eastern-Africa | 40.326 | -1.008 | -1.266 | 133.360 | -2.833 |
| SC0508 | PI533999 | Durra-Bicolor | 0 | 0 | Ethiopia | Eastern-Africa | 41.286 | -6.792 | -6.808 | 141.787 | 1.265 |
| SC0512 | PI534008 | Guinea | 0 | 0 | India | India | 32.026 | -13.545 | 32.969 | -17.141 | 61.706 |
| SC0514 | PI534019 | Guinea | 0 | 0 | India | India | 18.716 | -17.805 | 51.959 | -9.091 | 62.686 |
| SC0515 | PI534012 | Guinea | 0 | NA | India | India | -0.153 | -26.026 | 59.564 | -12.768 | 78.453 |
| SC0516 | PI576408 | Guinea | 0 | 0 | India | India | 16.139 | -19.874 | 43.888 | -10.022 | 65.084 |
| SC0517 | NA | Guinea | 0 | 0 | Ghana | Western-Africa | -9.148 | -50.458 | 18.686 | -9.933 | 35.791 |
| SC0519 | PI534078 | Guinea | 0 | 1 | Nigeria | Western-Africa | -32.523 | -107.541 | -39.237 | -6.944 | -18.293 |
| SC0520 | PI534080 | Guinea | 0 | 1 | Nigeria | Western-Africa | -34.975 | -114.502 | -32.816 | -10.194 | -2.379 |
| SC0521 | NA | Guinea | 0 | 0 | Nigeria | Western-Africa | -25.865 | -98.973 | -10.608 | -11.836 | -6.395 |
| SC0522 | PI534081 | Guinea | 0 | 0 | Nigeria | Western-Africa | -38.167 | -113.798 | -43.832 | -12.300 | -0.315 |
| SC0523 | PI534083 | Guinea | 0 | 1 | Nigeria | Western-Africa | -39.234 | -115.649 | -41.853 | -12.944 | 2.668 |
| SC0525 | PI656101 | Guinea | 0 | 0 | Nigeria | Western-Africa | -32.396 | -103.638 | -29.686 | -14.914 | -6.500 |
| SC0526 | PI534086 | Guinea | 0 | 0 | Nigeria | Western-Africa | -32.425 | -105.270 | -32.166 | -15.536 | -2.228 |
| SC0527 | PI534087 | Guinea | 0 | 0 | Nigeria | Western-Africa | -31.059 | -97.422 | -32.896 | -11.974 | -7.816 |
| SC0528 | PI533889 | Guinea | 0 | 0 | Nigeria | Western-Africa | -31.527 | -95.177 | -42.596 | -8.390 | -21.755 |
| SC0529 | PI534090 | Guinea | 0 | 0 | Nigeria | Western-Africa | -35.084 | -105.482 | -31.409 | -11.573 | -2.765 |
| SC0530 | PI533892 | Guinea | 0 | NA | Nigeria | Western-Africa | -33.420 | -103.078 | -35.932 | -14.642 | -2.323 |
| SC0532 | PI597951 | Guinea | 0 | 0 | BurkinaFaso | Western-Africa | -23.614 | -96.992 | -29.153 | -6.138 | -30.683 |
| SC0534 | PI533886 | Guinea | 0 | 0 | Nigeria | Western-Africa | -29.239 | -112.802 | -37.529 | -11.117 | -1.391 |
| SC0536 | PI534084 | Guinea | 0 | 0 | Nigeria | Western-Africa | -34.168 | -112.690 | -37.859 | -11.400 | -0.158 |
| SC0537 | PI597954 | Guinea | 0 | 0 | Nigeria | Western-Africa | -30.203 | -108.255 | -36.943 | -6.934 | -2.811 |
| SC0538 | PI534091 | Guinea | 0 | 0 | Nigeria | Western-Africa | -21.561 | -71.021 | -16.785 | -13.982 | 3.114 |

Table S4.1. continued

| SC0540 | NA | Guinea | 0 | 0 | Nigeria | Western-Africa | -32.104 | -96.975 | -9.858 | -12.516 | -4.059 |
| --- | --- | --- | --- | --- | --- | --- | --- | --- | --- | --- | --- |
| SC0541 | PI534094 | Guinea | 0 | 0 | Nigeria | Western-Africa | -31.298 | -113.396 | -40.594 | -12.923 | -1.007 |
| SC0542 | NA | Guinea | 0 | 0 | Nigeria | Western-Africa | -14.670 | -20.335 | 28.721 | -11.706 | -6.634 |
| SC0543 | PI534071 | Guinea | 0 | 0 | Nigeria | Western-Africa | -26.495 | -107.453 | -32.340 | -13.646 | -6.244 |
| SC0544 | PI534069 | Guinea | 0 | 0 | Nigeria | Western-Africa | -33.826 | -114.587 | -35.245 | -10.542 | -1.594 |
| SC0545 | PI534072 | Guinea | 0 | 0 | Nigeria | Western-Africa | -25.497 | -105.701 | -32.426 | -11.984 | -4.831 |
| SC0546 | PI534077 | Guinea | 0 | 0 | Nigeria | Western-Africa | -34.623 | -105.681 | -33.414 | -12.345 | 0.704 |
| SC0547 | NA | Guinea | 0 | 0 | Nigeria | Western-Africa | -27.793 | -95.264 | -26.976 | -11.961 | -0.538 |
| SC0549 | PI533840 | Guinea | 0 | 0 | Nigeria | Western-Africa | -34.609 | -110.181 | -41.253 | -13.320 | -0.395 |
| SC0550 | NA | Guinea | 0 | 0 | BurkinaFaso | Western-Africa | -4.547 | -56.914 | -0.488 | 23.460 | 24.364 |
| SC0551 | NA | Guinea | 0 | 0 | Nigeria | Western-Africa | -34.797 | -112.677 | -46.434 | -14.457 | -1.199 |
| SC0553 | PI534063 | Durra | 0 | 0 | Nigeria | Western-Africa | -33.123 | -97.606 | -40.607 | -5.788 | -20.813 |
| SC0556 | NA | Kafir | 0 | 0 | Bechuanaland | Unknown | 7.504 | -5.210 | 107.045 | -12.588 | -38.675 |
| SC0557 | PI533939 | Caudatum | 1 | 0 | Mozambique | Eastern-Africa | -25.936 | -9.642 | 26.809 | 0.200 | 20.649 |
| SC0558 | PI533938 | Caudatum | 0 | 0 | Zaire | Middle-Africa | -16.210 | -10.963 | 37.317 | 2.343 | 7.663 |
| SC0559 | PI534001 | Caudatum | 0 | NA | Ethiopia | Eastern-Africa | -51.583 | 45.508 | -9.649 | 0.594 | -2.818 |
| SC0562 | PI533987 | Caudatum | 0 | NA | Sudan | Northern-Africa | -37.987 | 15.944 | -24.187 | -5.858 | 18.486 |
| SC0563 | PI533876 | Caudatum | 0 | 0 | Nigeria | Western-Africa | -27.898 | -1.420 | -12.491 | -6.433 | 14.377 |
| SC0564 | PI534053 | Caudatum | 0 | 0 | Uganda | Eastern-Africa | -37.041 | 17.673 | -19.105 | -3.789 | 16.223 |
| SC0565 | PI534055 | Caudatum | 0 | 0 | Niger | Western-Africa | -24.617 | -51.494 | 3.853 | -9.168 | 31.454 |
| SC0566 | PI533871 | Caudatum | 0 | 0 | Nigeria | Western-Africa | -36.632 | -82.283 | -32.984 | -10.106 | 5.808 |
| SC0567 | PI534093 | Caudatum | 0 | 0 | Nigeria | Western-Africa | -22.719 | -79.791 | -26.804 | -14.061 | 2.144 |
| SC0568 | PI576343 | Caudatum | 0 | 0 | Nigeria | Western-Africa | -22.204 | -76.939 | -20.420 | -11.705 | -8.201 |
| SC0569 | PI534092 | Caudatum | 0 | 0 | Nigeria | Western-Africa | -31.252 | -70.207 | -32.810 | -15.120 | -34.761 |
| SC0572 | PI533980 | Caudatum | 0 | 0 | China | Asia | -33.202 | 32.114 | -18.589 | 3.050 | -3.450 |
| SC0574 | PI534114 | Caudatum | 0 | 0 | Pakistan | Asia | -61.860 | 38.595 | -14.852 | -4.717 | 23.643 |
| SC0575 | PI533992 | Caudatum | 0 | 0 | Sudan | Northern-Africa | -27.319 | 27.665 | -20.409 | 3.134 | -2.678 |
| SC0577 | PI533978 | Caudatum | 0 | 0 | Sudan | Northern-Africa | -25.884 | 27.081 | -21.150 | 1.181 | -6.980 |
| SC0578 | PI533870 | Caudatum | 0 | 0 | Nigeria | Western-Africa | -23.619 | -61.062 | -22.370 | 0.322 | -18.161 |
| SC0580 | PI534006 | Durra | 1 | 0 | India | India | 107.140 | 20.994 | -32.247 | -40.270 | 32.022 |
| SC0581 | NA | Durra | 0 | 0 | India | India | 81.942 | 11.296 | -13.486 | -29.852 | 21.397 |
| SC0582 | NA | Durra-Bicolor | 0 | 0 | India | India | 42.094 | 0.163 | 31.717 | -6.156 | 23.206 |

Table S4.1. continued

| SC0584 | PI534015 | Durra | 0 | 1 | India | India | 106.548 | 30.149 | -20.159 | -33.341 | -2.094 |
| --- | --- | --- | --- | --- | --- | --- | --- | --- | --- | --- | --- |
| SC0586 | PI534035 | Durra | 1 | 1 | India | India | 111.723 | 32.598 | -27.838 | -33.321 | -0.538 |
| SC0587 | PI534021 | Durra | 1 | 1 | India | India | 112.641 | 21.969 | -36.766 | -33.589 | 12.859 |
| SC0589 | PI534023 | Durra | 1 | 1 | India | India | 115.445 | 24.332 | -31.424 | -34.557 | 4.107 |
| SC0590 | PI534102 | Caudatum | 0 | 0 | Uganda | Eastern-Africa | -15.107 | -2.748 | 20.511 | 6.056 | 11.230 |
| SC0593 | PI576344 | Durra-Bicolor | 0 | 0 | Ethiopia | Eastern-Africa | 16.593 | -4.716 | 18.913 | 89.624 | -2.463 |
| SC0598 | PI576337 | Unknown/Other | 0 | 0 | Uganda | Eastern-Africa | -28.868 | 13.357 | 47.445 | -17.128 | -3.309 |
| SC0599 | PI534163 | Caudatum | 0 | 0 | USA | Americas | -58.530 | 37.530 | 5.712 | -8.562 | 18.258 |
| SC0600 | PI576336 | Guinea | 0 | 0 | Sudan | Northern-Africa | -36.386 | -43.805 | -21.282 | -9.084 | 15.290 |
| SC0601 | PI534103 | Guinea | 0 | 0 | Uganda | Eastern-Africa | -33.437 | -3.333 | 34.331 | -7.805 | 36.178 |
| SC0602 | NA | Guinea | 0 | 0 | Uganda | Eastern-Africa | -2.152 | 8.327 | 79.783 | -7.859 | -24.834 |
| SC0603 | PI533936 | Guinea | 0 | 0 | Tanzania | Eastern-Africa | -0.755 | -28.496 | 90.175 | -11.654 | 8.921 |
| SC0604 | NA | Guinea | 0 | 0 | Africa | Eastern-Africa | -24.429 | -45.069 | 10.278 | -9.902 | 17.284 |
| SC0605 | PI534096 | Guinea | 0 | 1 | Kenya | Eastern-Africa | -7.148 | -49.950 | 2.406 | 23.279 | 23.822 |
| SC0606 | PI597946 | Guinea-Bicolor | 0 | 0 | China | Asia | -13.867 | 23.957 | 26.814 | 4.564 | -17.617 |
| SC0609 | PI576332 | Bicolor | 0 | 0 | China | Asia | 4.701 | -20.104 | 99.281 | -7.473 | 18.365 |
| SC0610 | PI656103 | Bicolor | 0 | 0 | China | Asia | 57.444 | -1.341 | -17.601 | -6.842 | 26.267 |
| SC0614 | PI533940 | Bicolor | 0 | 0 | Tanzania | Eastern-Africa | 12.109 | -21.362 | 90.479 | -15.348 | 6.279 |
| SC0615 | NA | Bicolor-Kafir | 0 | 0 | India | India | 41.559 | 1.614 | 49.300 | -9.558 | -8.348 |
| SC0616 | NA | Bicolor | 0 | 0 | India | India | 65.563 | -5.277 | -5.458 | -26.662 | 24.481 |
| SC0618 | NA | Bicolor | 0 | 1 | Burma | Asia | 71.262 | -1.924 | 1.968 | -27.001 | 36.155 |
| SC0620 | PI576341 | Bicolor | 0 | 1 | S.Africa | Southern-Africa | 8.245 | -32.122 | 35.841 | -1.576 | 26.499 |
| SC0621 | PI656104 | Bicolor | NA | 1 | India | India | 63.529 | -1.813 | 2.109 | -16.568 | 33.906 |
| SC0623 | PI533956 | Durra | 0 | 0 | Congo | Middle-Africa | -6.252 | -5.278 | 45.445 | 1.222 | -0.870 |
| SC0624 | PI576366 | Durra | 0 | 0 | India | India | 52.560 | 4.971 | 1.385 | -10.359 | 20.755 |
| SC0625 | PI534097 | Kafir | 0 | 1 | Japan | Asia | 16.349 | -11.260 | 100.444 | -8.747 | -11.440 |
| SC0626 | PI534098 | Kafir | 0 | 0 | Japan | Asia | 7.743 | -20.783 | 104.419 | -9.017 | 2.468 |
| SC0627 | PI576345 | Kafir | 0 | 0 | S.Africa | Southern-Africa | -4.416 | -6.457 | 115.072 | -10.654 | -16.960 |
| SC0628 | PI533979 | Kafir | 0 | 0 | S.Africa | Southern-Africa | 8.070 | -12.367 | 123.771 | -10.002 | -27.493 |
| SC0629 | PI597947 | Kafir | 0 | 0 | Botswana | Southern-Africa | -8.881 | -11.678 | 110.921 | -12.863 | -3.426 |
| SC0630 | PI533937 | Kafir | 0 | 0 | Zambia | Eastern-Africa | -9.035 | -10.377 | 122.502 | -14.039 | -7.833 |
| SC0631 | NA | Kafir | 0 | 0 | Bechuanaland | Unknown | 0.478 | -16.257 | 115.654 | -15.839 | -5.097 |

Table S4.1. continued

| SC0632 | PI576400 | Kafir | 0 | 0 | Unknown | Unknown | 1.039 | -20.199 | 137.562 | -19.539 | -19.157 |
| --- | --- | --- | --- | --- | --- | --- | --- | --- | --- | --- | --- |
| SC0634 | PI533929 | Kafir | 0 | 0 | Mexico | Americas | 8.708 | -8.352 | 101.480 | -13.390 | 8.405 |
| SC0635 | PI576329 | Kafir | 0 | 0 | USA | Americas | 1.334 | -17.707 | 134.345 | -18.410 | -13.815 |
| SC0636 | NA | Kafir-Caudatum | 0 | 0 | India | India | 61.557 | 5.686 | 40.467 | -24.695 | -24.319 |
| SC0637 | PI534105 | Kafir-Caudatum | 0 | 0 | Uganda | Eastern-Africa | -52.656 | 44.836 | -2.512 | -2.232 | -2.828 |
| SC0639 | PI656105 | Kafir-Caudatum | 0 | 0 | India | India | -40.451 | 35.843 | 24.765 | -4.211 | -9.975 |
| SC0640 | NA | Kafir-Caudatum | NA | 0 | India | India | -44.184 | 40.704 | 21.196 | -6.974 | -8.505 |
| SC0641 | PI534104 | Kafir-Caudatum | 0 | 0 | Uganda | Eastern-Africa | -64.451 | 57.843 | -14.196 | -3.782 | 5.837 |
| SC0642 | PI534109 | Kafir-Caudatum | 0 | 0 | Uganda | Eastern-Africa | -65.186 | 59.524 | -12.666 | -3.077 | 2.340 |
| SC0643 | PI534110 | Kafir-Caudatum | 0 | 0 | Uganda | Eastern-Africa | -57.631 | 52.731 | -1.427 | -4.538 | 4.064 |
| SC0644 | PI534111 | Kafir-Caudatum | 0 | 0 | Uganda | Eastern-Africa | -64.383 | 56.369 | -19.623 | -4.980 | 7.304 |
| SC0645 | PI534108 | Kafir-Caudatum | 0 | 0 | Uganda | Eastern-Africa | -57.706 | 53.596 | -1.329 | -3.278 | 3.950 |
| SC0646 | PI534107 | Kafir-Caudatum | 0 | 0 | Uganda | Eastern-Africa | -2.259 | -9.020 | 108.425 | -11.243 | -23.741 |
| SC0647 | PI533952 | Kafir-Caudatum | 0 | 0 | S.Africa | Southern-Africa | -4.032 | -11.317 | 114.347 | -8.979 | -16.283 |
| SC0648 | PI533955 | Kafir-Caudatum | 0 | 0 | S.Africa | Southern-Africa | 1.095 | -17.526 | 114.092 | -9.699 | -4.720 |
| SC0649 | PI576338 | Kafir-Caudatum | 0 | 0 | Zimbabwe | Eastern-Africa | -1.380 | -13.809 | 125.563 | -11.053 | -11.770 |
| SC0650 | PI576340 | Kafir-Caudatum | 0 | 0 | S.Africa | Southern-Africa | 53.032 | 1.780 | 34.452 | -21.247 | 18.865 |
| SC0652 | NA | Kafir-Caudatum | 0 | 0 | India | India | 3.107 | -11.718 | 124.463 | -12.195 | -18.389 |
| SC0653 | PI533953 | Kafir-Caudatum | 0 | 0 | S.Africa | Southern-Africa | -17.632 | 6.351 | 11.712 | -7.626 | -5.273 |
| SC0654 | PI533975 | Kafir-Caudatum | 0 | 0 | Mozambique | Eastern-Africa | 6.999 | -13.107 | 123.498 | -10.132 | -23.106 |
| SC0655 | PI533976 | Kafir-Caudatum | 0 | 0 | S.Africa | Southern-Africa | -52.606 | 41.218 | -12.381 | 1.655 | 5.214 |
| SC0657 | PI533982 | Kafir-Caudatum | 0 | 0 | S.Africa | Southern-Africa | 1.949 | -13.306 | 122.270 | -10.897 | -5.842 |
| SC0659 | PI576333 | Guinea-Kafir | 0 | 0 | USA | Americas | -1.789 | -20.166 | 119.124 | -11.063 | 0.729 |
| SC0663 | PI533948 | Guinea-Kafir | 0 | 0 | USA | Americas | -3.568 | -18.477 | 121.233 | -11.586 | -8.762 |
| SC0671 | PI534054 | Kafir | 0 | 0 | Kenya | Eastern-Africa | -12.058 | -24.451 | 80.073 | 1.578 | 31.006 |
| SC0672 | PI595702 | Kafir-Caudatum | 0 | 0 | Zimbabwe | Eastern-Africa | -2.757 | -12.431 | 110.411 | -7.821 | -1.375 |
| SC0673 | PI576339 | Kafir | 0 | 0 | Zimbabwe | Eastern-Africa | -1.798 | -16.848 | 117.962 | -9.549 | -0.119 |
| SC0679 | PI534046 | Guinea-Caudatum | 0 | 0 | Sudan | Northern-Africa | -24.611 | 9.928 | 9.562 | 20.777 | 12.160 |
| SC0680 | PI576374 | Caudatum | 0 | 0 | Tanzania | Eastern-Africa | -73.248 | 58.948 | -23.807 | -7.407 | 23.789 |
| SC0681 | PI533994 | Caudatum | 0 | 0 | Sudan | Northern-Africa | -73.495 | 55.047 | -13.024 | -6.359 | 7.821 |
| SC0682 | PI576392 | Caudatum | 0 | 0 | S.Africa | Southern-Africa | -1.743 | -14.330 | 116.395 | -14.827 | -9.376 |
| SC0683 | PI533963 | Caudatum | 0 | 0 | Sudan | Northern-Africa | -63.025 | 50.972 | -10.238 | -7.207 | 4.077 |

Table S4.1. continued

| SC0685 | NA | Caudatum | 0 | 0 | FrenchEquatorial | Middle-Africa | -57.886 | 49.359 | -17.514 | -0.653 | -10.658 |
| --- | --- | --- | --- | --- | --- | --- | --- | --- | --- | --- | --- |
| SC0686 | PI534113 | Caudatum | 0 | 0 | Uganda | Eastern-Africa | -74.137 | 61.616 | -21.771 | -7.690 | 11.664 |
| SC0687 | PI534038 | Caudatum | 0 | 0 | Sudan | Northern-Africa | -77.065 | 57.825 | -23.612 | -5.364 | 4.090 |
| SC0689 | PI533969 | Caudatum | 0 | 0 | Uganda | Eastern-Africa | -58.530 | 50.110 | -14.776 | -4.951 | 0.883 |
| SC0690 | PI533971 | Caudatum | 0 | 0 | Uganda | Eastern-Africa | -73.414 | 61.113 | -17.333 | -7.383 | 10.286 |
| SC0691 | PI534050 | Caudatum | 0 | 0 | Cent.Afr.Rep. | Middle-Africa | -59.585 | 43.438 | -13.463 | -4.925 | 3.818 |
| SC0692 | PI533966 | Caudatum | 0 | 0 | Uganda | Eastern-Africa | -69.923 | 61.874 | -14.391 | -2.880 | 2.014 |
| SC0693 | PI534052 | Caudatum | 0 | 0 | Uganda | Eastern-Africa | -78.134 | 61.575 | -16.597 | -9.786 | 12.856 |
| SC0694 | PI534056 | Caudatum | 0 | 0 | Nigeria | Western-Africa | -68.830 | 52.364 | -12.160 | -5.514 | 5.668 |
| SC0695 | PI656106 | Caudatum | 0 | 0 | Tanzania | Eastern-Africa | -65.337 | 44.054 | -9.212 | -8.910 | 25.387 |
| SC0700 | PI576346 | Caudatum | 0 | 0 | S.Africa | Southern-Africa | -19.102 | -21.118 | 73.402 | -14.381 | 19.345 |
| SC0701 | PI533985 | Caudatum | 0 | 0 | Sudan | Northern-Africa | -60.475 | 41.503 | -26.987 | -4.282 | 20.948 |
| SC0702 | PI656107 | Caudatum | 0 | 0 | Sudan | Northern-Africa | -38.163 | 29.912 | -15.758 | -1.752 | 19.497 |
| SC0704 | PI534099 | Caudatum | 0 | 0 | Japan | Asia | -67.760 | 48.138 | -24.118 | -6.205 | 22.626 |
| SC0705 | PI576420 | Caudatum | 0 | 0 | Japan | Asia | -59.327 | 36.815 | -14.531 | -4.206 | 22.388 |
| SC0706 | PI659693 | Caudatum | 0 | 0 | Sudan | Northern-Africa | -66.525 | 56.020 | -17.520 | -3.656 | 0.891 |
| SC0707 | PI533959 | Caudatum | 0 | 0 | Unknown | Unknown | -58.781 | 49.744 | -13.738 | -5.984 | 6.829 |
| SC0708 | PI533970 | Caudatum | 0 | 0 | Uganda | Eastern-Africa | -63.105 | 55.566 | -23.672 | -3.185 | 13.408 |
| SC0709 | PI533990 | Caudatum | 0 | 0 | Sudan | Northern-Africa | -20.421 | 19.221 | 37.587 | -4.599 | -21.117 |
| SC0712 | PI576372 | Caudatum | 0 | 0 | Japan | Asia | -68.851 | 45.867 | -23.530 | -4.708 | 14.881 |
| SC0713 | NA | Caudatum | 0 | 0 | Sudan | Northern-Africa | -61.546 | 49.584 | -22.443 | -3.936 | -2.682 |
| SC0715 | PI534039 | Caudatum | 0 | 0 | Sudan | Northern-Africa | -68.239 | 57.876 | -23.320 | -5.944 | 1.564 |
| SC0716 | PI576355 | Caudatum | 0 | 0 | Sudan | Northern-Africa | -55.828 | 42.943 | -24.229 | -6.849 | 11.684 |
| SC0719 | PI534047 | Caudatum | 0 | 0 | Sudan | Northern-Africa | -45.569 | 38.958 | -16.618 | -0.369 | -8.964 |
| SC0720 | PI659696 | Caudatum | 0 | 0 | Kenya | Eastern-Africa | -47.314 | 28.189 | 35.846 | -9.822 | 3.208 |
| SC0721 | PI534100 | Caudatum | 0 | 0 | Japan | Asia | -20.365 | 7.392 | 66.601 | -13.083 | -22.947 |
| SC0723 | PI533988 | Caudatum | 0 | 0 | Sudan | Northern-Africa | -76.805 | 61.118 | -20.773 | -4.485 | 1.686 |
| SC0724 | PI533993 | Caudatum | 0 | 0 | Sudan | Northern-Africa | -61.027 | 50.213 | -32.467 | -1.928 | 5.947 |
| SC0725 | PI534101 | Caudatum | 0 | 0 | Japan | Asia | -52.456 | 38.311 | -16.555 | -6.552 | 12.587 |
| SC0726 | PI534051 | Caudatum | 0 | 0 | Cent.Afr.Rep. | Middle-Africa | -68.554 | 58.317 | -22.843 | -5.103 | 4.728 |
| SC0727 | PI534045 | Caudatum | 0 | 0 | Sudan | Northern-Africa | -65.195 | 54.606 | -15.975 | -3.702 | 3.790 |
| SC0728 | PI533968 | Caudatum | 0 | 0 | Uganda | Eastern-Africa | -45.197 | 43.361 | -14.919 | 1.271 | -7.888 |

Table S4.1. continued

| SC0730 | PI576411 | Caudatum | 0 | 0 | Sudan | Northern-Africa | -60.679 | 53.784 | -31.933 | -0.820 | 3.980 |
| --- | --- | --- | --- | --- | --- | --- | --- | --- | --- | --- | --- |
| SC0731 | PI576356 | Caudatum | 0 | 0 | Sudan | Northern-Africa | -63.340 | 53.781 | -24.093 | -6.096 | 3.451 |
| SC0732 | PI534049 | Caudatum | 0 | 0 | Sudan | Northern-Africa | -70.738 | 59.231 | -20.552 | -5.656 | 4.756 |
| SC0733 | PI534061 | Caudatum | 0 | 0 | Nigeria | Western-Africa | -56.038 | 45.472 | -30.004 | 2.782 | 9.194 |
| SC0734 | PI576394 | Caudatum | 0 | 0 | Sudan | Northern-Africa | -7.544 | -4.882 | 105.241 | -14.820 | -22.459 |
| SC0736 | PI534040 | Caudatum | 0 | 0 | Sudan | Northern-Africa | -19.822 | 19.032 | 52.031 | -2.068 | -11.472 |
| SC0737 | PI533983 | Caudatum | 0 | 0 | Sudan | Northern-Africa | -71.544 | 56.401 | -26.591 | -3.004 | 9.518 |
| SC0738 | PI597952 | Caudatum | 0 | 1 | Sudan | Northern-Africa | -47.665 | 38.791 | -6.814 | -6.478 | -0.886 |
| SC0741 | NA | Caudatum | 0 | 0 | Nigeria | Western-Africa | 86.521 | 20.168 | -4.167 | -21.864 | -17.887 |
| SC0747 | NA | Caudatum | 0 | 0 | via(Mexico) | Americas | -25.337 | 29.729 | 29.222 | -6.702 | -3.256 |
| SC0748 | PI533991 | Caudatum | 0 | 0 | Sudan | Northern-Africa | -53.013 | 52.481 | -4.289 | -4.306 | -10.589 |
| SC0749 | PI576373 | Caudatum-Bicolor | 0 | 0 | Japan | Asia | -4.146 | 19.166 | 46.009 | -0.854 | -36.789 |
| SC0751 | PI576354 | Caudatum | 0 | 0 | Sudan | Northern-Africa | -9.501 | -19.651 | 58.227 | -4.938 | 39.400 |
| SC0753 | NA | Caudatum-Kafir | 0 | 0 | India | India | -18.940 | 17.409 | 50.483 | -4.340 | -23.736 |
| SC0755 | PI576350 | Caudatum | 0 | 1 | USA | Americas | 5.895 | -11.025 | 42.870 | 11.673 | 12.164 |
| SC0756 | PI576368 | Caudatum | 0 | 0 | Sudan | Northern-Africa | -52.276 | 52.831 | -25.366 | 2.555 | -2.586 |
| SC0757 | PI576352 | Caudatum | 0 | 0 | Botswana | Southern-Africa | -29.295 | 16.180 | 65.134 | -6.747 | -4.610 |
| SC0759 | NA | Caudatum-Kafir | 0 | 0 | via(Mexico) | Americas | -37.091 | 25.921 | 5.609 | -4.647 | 7.587 |
| SC0760 | PI533949 | Caudatum | 0 | 0 | Sudan | Northern-Africa | -53.321 | 46.112 | -15.824 | -0.929 | -2.428 |
| SC0761 | PI533928 | Caudatum | 0 | 0 | Mexico | Americas | 3.885 | -15.354 | 110.494 | -10.034 | -9.067 |
| SC0762 | PI576363 | Caudatum | 0 | 0 | India | India | -66.979 | 59.263 | -24.867 | -1.448 | 3.788 |
| SC0763 | PI534041 | Caudatum | 0 | 0 | Sudan | Northern-Africa | -49.862 | 39.010 | -1.414 | -2.810 | -1.778 |
| SC0764 | PI533974 | Caudatum | 0 | 0 | Mozambique | Eastern-Africa | -55.814 | 47.743 | 3.253 | -6.600 | 2.069 |
| SC0770 | PI576328 | Caudatum | 0 | 0 | Swaziland | Southern-Africa | -49.163 | 25.871 | 11.582 | -5.574 | -5.842 |
| SC0773 | PI576351 | Caudatum | 0 | 0 | China | Asia | -62.825 | 58.155 | -24.796 | -2.344 | 2.407 |
| SC0774 | PI576353 | Caudatum | 0 | 0 | Sudan | Northern-Africa | -58.566 | 55.527 | -15.121 | -1.239 | 0.278 |
| SC0777 | NA | Caudatum-Kafir | 0 | 0 | Sudan | Northern-Africa | -52.004 | 44.830 | -0.610 | -3.939 | -3.128 |
| SC0779 | PI576413 | Caudatum | 0 | 0 | Sudan | Northern-Africa | -11.297 | 4.296 | 75.868 | -7.184 | -19.077 |
| SC0780 | PI533984 | Caudatum | 0 | 0 | Sudan | Northern-Africa | -55.339 | 46.060 | -23.617 | 0.877 | 7.915 |
| SC0781 | PI576371 | Caudatum | 0 | 0 | Nigeria | Western-Africa | -60.994 | 56.700 | -1.839 | -3.727 | 1.032 |
| SC0782 | PI576364 | Caudatum | 0 | 0 | India | India | 1.417 | -10.163 | 101.282 | -11.698 | 2.095 |
| SC0784 | PI576369 | Caudatum | 0 | 0 | Cent.Afr.Rep. | Middle-Africa | -60.685 | 40.450 | -18.683 | -4.405 | 8.052 |

Table S4.1. continued

| SC0790 | PI656120 | Caudatum | 0 | 0 | India | India | -47.787 | 46.873 | 1.687 | -9.828 | -17.319 |
| --- | --- | --- | --- | --- | --- | --- | --- | --- | --- | --- | --- |
| SC0797 | PI576397 | Caudatum | 0 | 1 | Zimbabwe | Eastern-Africa | -5.817 | -12.976 | 84.922 | -11.750 | 9.490 |
| SC0798 | PI533989 | Caudatum | 0 | 0 | Sudan | Northern-Africa | -67.998 | 55.919 | -21.336 | -6.135 | 7.647 |
| SC0800 | PI595709 | Caudatum | 0 | 0 | Sudan | Northern-Africa | -75.300 | 59.004 | -24.816 | -5.515 | 3.902 |
| SC0803 | PI533964 | Caudatum | 0 | 0 | Sudan | Northern-Africa | -75.349 | 61.207 | -28.331 | -5.201 | 8.515 |
| SC0804 | PI534043 | Caudatum | 0 | 0 | Sudan | Northern-Africa | -66.977 | 57.786 | -22.917 | -6.134 | 6.998 |
| SC0805 | PI533967 | Caudatum | 1 | 0 | Uganda | Eastern-Africa | -61.562 | 47.713 | -11.589 | -6.997 | 14.398 |
| SC0807 | PI576370 | Caudatum | 0 | 0 | Zimbabwe | Eastern-Africa | 4.157 | 14.869 | 6.296 | 3.744 | -23.680 |
| SC0808 | PI597944 | Caudatum-Durra | 0 | 0 | Uganda | Eastern-Africa | -30.699 | 31.147 | 11.278 | -1.145 | -17.188 |
| SC0810 | PI533930 | Caudatum | 0 | 0 | Sudan | Northern-Africa | -60.915 | 52.100 | -7.751 | -2.135 | 2.879 |
| SC0814 | NA | Durra | 0 | 0 | via(Mexico) | Americas | 62.087 | 3.904 | 21.052 | -24.690 | -3.238 |
| SC0817 | PI576330 | Durra | 0 | 0 | India | India | 90.006 | 15.897 | -30.678 | -33.328 | 16.817 |
| SC0819 | PI595700 | Durra | 1 | 0 | India | India | 91.318 | 13.762 | -27.167 | -36.409 | 30.489 |
| SC0821 | PI595701 | Kafir | 0 | 0 | S.Africa | Southern-Africa | 1.673 | -18.650 | 110.720 | -14.331 | 10.667 |
| SC0823 | NA | Durra | 0 | 0 | Mozambique | Eastern-Africa | 40.862 | -2.019 | -4.751 | 144.934 | 4.247 |
| SC0826 | PI576402 | Durra | 0 | 0 | India | India | 85.529 | 17.958 | -30.432 | -33.531 | 28.551 |
| SC0827 | PI576403 | Durra | 0 | 0 | India | India | 81.975 | 19.762 | -24.493 | -26.289 | 17.674 |
| SC0830 | PI595704 | Durra | 0 | 0 | India | India | 77.574 | 13.243 | -20.607 | -24.077 | 17.275 |
| SC0831 | PI576404 | Durra | 0 | 0 | India | India | 94.036 | 14.886 | -26.905 | -32.912 | 24.764 |
| SC0832 | PI534005 | Durra | 0 | 0 | India | India | 102.509 | 22.575 | -33.446 | -38.681 | 19.604 |
| SC0833 | PI656108 | Durra | 0 | 0 | India | India | 95.567 | 14.238 | -22.446 | -35.517 | 31.091 |
| SC0834 | NA | Durra | 0 | 0 | India | India | 84.333 | 16.574 | -27.160 | -25.311 | 16.290 |
| SC0835 | PI534007 | Durra | 0 | 0 | India | India | 98.255 | 12.617 | -24.070 | -36.378 | 27.398 |
| SC0837 | NA | Durra | 0 | 0 | India | India | 77.698 | 2.761 | 8.952 | -31.022 | 3.888 |
| SC0839 | PI576406 | Durra | 0 | 0 | India | India | 96.902 | 13.934 | -27.943 | -38.165 | 24.075 |
| SC0841 | PI595708 | Durra | 0 | 0 | India | India | 41.906 | -0.821 | 36.913 | -18.397 | 16.414 |
| SC0842 | PI576367 | Durra | 0 | 0 | India | India | 2.650 | -15.934 | 112.928 | -8.170 | -3.756 |
| SC0847 | NA | Durra | 0 | 0 | India | India | 88.885 | 17.080 | -23.772 | -33.897 | 18.506 |
| SC0848 | NA | Durra | 0 | 0 | India | India | 100.366 | 15.282 | -34.368 | -37.805 | 28.763 |
| SC0851 | PI576342 | Durra | 0 | 0 | Egypt | Northern-Africa | 22.062 | -4.050 | 34.577 | -4.033 | -12.553 |
| SC0852 | PI534000 | Durra | 0 | 0 | Ethiopia | Eastern-Africa | 47.955 | 5.595 | -19.927 | -2.821 | -7.863 |
| SC0854 | NA | Durra | 0 | 0 | India | India | 58.464 | 6.899 | 23.729 | -21.898 | -2.032 |

Table S4.1. continued

| SC0855 | PI597945 | Durra | 0 | 0 | Egypt | Northern-Africa | 32.852 | 11.306 | -2.216 | 11.584 | -28.950 |
| --- | --- | --- | --- | --- | --- | --- | --- | --- | --- | --- | --- |
| SC0858 | NA | Durra | 0 | 0 | India | India | 78.508 | 19.609 | 12.391 | -23.090 | -25.303 |
| SC0859 | PI534003 | Durra | 1 | 0 | India | India | 96.569 | 16.813 | -28.772 | -28.921 | 10.376 |
| SC0860 | NA | Durra | 0 | 0 | India | India | 91.223 | 24.043 | -23.466 | -17.674 | -12.793 |
| SC0863 | PI576360 | Durra | 0 | 0 | India | India | 91.287 | 27.073 | -20.848 | -21.050 | -4.959 |
| SC0864 | NA | Durra | 0 | 0 | India | India | 67.891 | 13.409 | 12.511 | -19.143 | -15.223 |
| SC0865 | PI534004 | Durra | 0 | 0 | India | India | 71.178 | 18.797 | -11.068 | -19.504 | -3.864 |
| SC0868 | NA | Durra | 0 | 0 | India | India | -7.994 | 1.142 | 30.834 | -8.949 | -6.720 |
| SC0870 | NA | Durra | 0 | 0 | India | India | 84.241 | 19.166 | 4.420 | -25.534 | -14.890 |
| SC0871 | NA | Durra | 0 | 0 | India | India | 96.455 | 26.743 | -5.681 | -23.288 | -22.354 |
| SC0875 | PI576358 | Durra | 0 | 0 | India | India | 91.089 | 26.030 | -19.246 | -25.645 | -2.420 |
| SC0876 | PI595706 | Durra | 0 | 0 | India | India | 106.268 | 29.363 | -27.483 | -29.377 | 2.974 |
| SC0877 | NA | Durra | 0 | 0 | India | India | 50.449 | 8.749 | 51.152 | -14.859 | -38.857 |
| SC0878 | NA | Durra | 0 | 0 | India | India | 75.423 | 18.757 | 5.335 | -16.449 | -18.177 |
| SC0888 | PI659753 | Durra | 0 | 1 | India | India | 101.350 | 22.029 | -29.375 | -28.593 | 6.131 |
| SC0891 | PI595707 | Durra | 0 | NA | India | India | 100.055 | 28.019 | -29.087 | -27.558 | -3.663 |
| SC0893 | NA | Durra | 0 | 0 | India | India | 59.379 | 11.094 | 51.368 | -18.093 | -36.602 |
| SC0895 | NA | Durra | 0 | 0 | India | India | 80.377 | 21.571 | 8.301 | -23.917 | -22.421 |
| SC0899 | PI534020 | Durra | 0 | 0 | India | India | 72.623 | 16.575 | -31.284 | -21.297 | 25.744 |
| SC0902 | PI576365 | Durra | 0 | 0 | India | India | 87.618 | 26.457 | -20.863 | -19.324 | -8.278 |
| SC0905 | PI534068 | Durra | 0 | 0 | Nigeria | Western-Africa | 95.089 | 30.034 | -18.989 | -27.406 | -4.506 |
| SC0906 | PI534048 | Caudatum | 0 | 0 | Sudan | Northern-Africa | -46.961 | 62.176 | -22.645 | -1.614 | -7.378 |
| SC0910 | PI576359 | Guinea-Durra | 0 | 1 | India | India | 64.992 | 6.574 | -0.718 | -23.326 | 34.898 |
| SC0913 | PI576410 | Durra-Bicolor | 0 | 0 | Sudan | Northern-Africa | -35.201 | 18.147 | 36.665 | -1.782 | 3.758 |
| SC0914 | NA | Durra-Bicolor | 0 | 0 | India | India | -51.097 | 53.796 | -13.064 | 3.121 | -3.492 |
| SC0919 | PI576407 | Durra | 0 | 0 | India | India | 95.988 | 27.363 | -22.739 | -22.562 | -9.931 |
| SC0921 | NA | Durra | 0 | 1 | India | India | 69.583 | 2.917 | 17.386 | -30.877 | 10.643 |
| SC0923 | NA | Durra | 0 | 1 | India | India | -12.800 | 1.564 | 9.970 | -6.007 | -0.595 |
| SC0924 | PI534029 | Durra | NA | NA | India | India | 98.025 | 24.869 | -14.261 | -30.957 | -8.393 |
| SC0929 | PI595699 | Durra | 0 | 0 | India | India | 96.547 | 26.506 | -22.833 | -26.007 | -8.363 |
| SC0935 | PI576388 | Durra | 0 | NA | USA | Americas | 36.746 | 22.003 | -13.562 | 5.031 | -56.942 |
| SC0937 | PI576348 | Bicolor | 0 | 1 | USA | Americas | 12.749 | -13.496 | 17.451 | 10.310 | 23.693 |

Table S4.1. continued

| SC0941 | PI576347 | Bicolor | 0 | 1 | USA | Americas | 1.120 | -24.266 | 50.914 | 1.949 | 40.858 |
| --- | --- | --- | --- | --- | --- | --- | --- | --- | --- | --- | --- |
| SC0942 | PI576349 | Bicolor | 0 | 1 | USA | Americas | 11.115 | -13.374 | 13.599 | 13.698 | 25.278 |
| SC0947 | PI656121 | Unknown/Other | 0 | 0 | India | India | -45.527 | 6.086 | -11.375 | -4.050 | 11.454 |
| SC0949 | PI533998 | Bicolor | 0 | 0 | USA | Americas | -20.225 | 15.805 | 26.483 | -1.311 | 3.228 |
| SC0950 | PI576389 | Caudatum-Bicolor | 0 | 0 | USA | Americas | 7.540 | -20.786 | 89.698 | -11.953 | 19.049 |
| SC0951 | PI576335 | Guinea | 0 | 0 | Sudan | Northern-Africa | -8.305 | -24.704 | 104.729 | -10.125 | 18.907 |
| SC0956 | PI576416 | Guinea | 0 | 0 | Nigeria | Western-Africa | -33.682 | -69.008 | -26.485 | -10.509 | 12.148 |
| SC0958 | PI576361 | Durra | 0 | 0 | India | India | 68.997 | 30.133 | -22.577 | -9.649 | -35.243 |
| SC0963 | PI533977 | Caudatum | 0 | 0 | S.Africa | Southern-Africa | -64.146 | 43.119 | -19.300 | 0.360 | 17.654 |
| SC0964 | PI533972 | Caudatum | 0 | 0 | Uganda | Eastern-Africa | -81.822 | 62.134 | -25.533 | -6.212 | 26.188 |
| SC0965 | NA | Unknown/Other | 0 | 0 | Uganda | Eastern-Africa | 28.863 | -0.385 | -11.640 | 17.522 | 12.276 |
| SC0968 | PI656110 | Unknown/Other | 0 | 0 | Zimbabwe | Eastern-Africa | 53.344 | 5.367 | -8.625 | 69.037 | -19.085 |
| SC0969 | PI534164 | Durra | 0 | 0 | Uganda | Eastern-Africa | -44.989 | 34.043 | -11.643 | -10.261 | 15.070 |
| SC0970 | PI576386 | Guinea-Caudatum | 0 | 0 | Uganda | Eastern-Africa | -55.071 | 38.975 | -17.230 | -4.243 | 23.411 |
| SC0971 | PI656111 | Unknown/Other | 1 | 1 | PuertoRico | Americas | -3.542 | -11.496 | 10.789 | -7.695 | 17.177 |
| SC0972 | PI534165 | Caudatum | 0 | 0 | Sudan | Northern-Africa | -75.453 | 64.198 | -30.219 | -4.052 | 2.080 |
| SC0975 | PI576379 | Durra-Bicolor | 0 | 0 | Ethiopia | Eastern-Africa | 40.109 | -0.809 | -2.966 | 148.530 | 1.671 |
| SC0979 | PI576428 | Caudatum | 0 | 0 | Ethiopia | Eastern-Africa | -74.359 | 62.731 | -15.641 | -8.883 | -6.970 |
| SC0982 | PI576380 | Caudatum | 0 | 0 | Ethiopia | Eastern-Africa | -66.655 | 60.899 | -25.716 | 0.767 | -7.189 |
| SC0984 | PI534115 | Caudatum | 0 | 0 | Ethiopia | Eastern-Africa | -80.869 | 67.741 | -27.974 | -8.309 | -2.297 |
| SC0987 | PI534116 | Durra-Bicolor | 0 | 0 | Ethiopia | Eastern-Africa | 32.616 | -0.496 | 6.450 | 78.985 | -15.251 |
| SC0991 | PI534117 | Bicolor | 0 | 0 | Uganda | Eastern-Africa | -19.782 | -15.055 | 38.834 | 10.361 | -3.815 |
| SC0997 | PI534166 | Unknown/Other | 0 | 0 | Israel | Arabia/MiddleEast | -33.831 | 15.990 | 48.586 | -13.398 | -6.934 |
| SC0998 | PI534167 | Durra-Bicolor | 0 | 0 | Unknown | Unknown | 27.481 | 2.135 | -8.401 | -0.658 | -8.665 |
| SC0999 | NA | Unknown/Other | 0 | 0 | Ethiopia | Eastern-Africa | 44.316 | -7.250 | -13.017 | 147.577 | 17.957 |
| SC1014 | PI576375 | Durra-Bicolor | 0 | 0 | Ethiopia | Eastern-Africa | 49.784 | 9.049 | -10.389 | 14.385 | -50.720 |
| SC1015 | NA | Unknown/Other | 0 | 0 | Ethiopia | Eastern-Africa | 52.749 | 17.596 | -9.205 | 19.166 | -50.901 |
| SC1017 | PI576376 | Durra-Bicolor | 0 | 0 | Ethiopia | Eastern-Africa | 59.498 | 21.472 | -19.282 | 15.260 | -51.504 |
| SC1019 | PI656071 | Caudatum | 0 | 0 | Ethiopia | Eastern-Africa | -45.132 | 44.126 | -18.634 | -3.231 | 1.905 |
| SC1021 | NA | Durra | 0 | 0 | Ethiopia | Eastern-Africa | 52.845 | 7.298 | 23.670 | 2.542 | -24.673 |
| SC1022 | PI576377 | Durra-Bicolor | 0 | 0 | Ethiopia | Eastern-Africa | 53.060 | -3.692 | -10.585 | 83.709 | 12.924 |
| SC1023 | NA | Unknown/Other | 0 | 0 | Ethiopia | Eastern-Africa | 42.914 | -0.139 | 26.560 | 50.460 | -29.194 |

Table S4.1. continued

| SC1024 | NA | Durra | 0 | 0 | Ethiopia | Eastern-Africa | 69.703 | 14.653 | -7.727 | 6.567 | -43.869 |
| --- | --- | --- | --- | --- | --- | --- | --- | --- | --- | --- | --- |
| SC1025 | PI576378 | Durra-Bicolor | 0 | 0 | Ethiopia | Eastern-Africa | -2.261 | 23.767 | -13.180 | 43.247 | 3.707 |
| SC1031 | PI597956 | Durra-Bicolor | 0 | 0 | Ethiopia | Eastern-Africa | 52.822 | 5.799 | -13.043 | 2.813 | -17.500 |
| SC1033 | PI576426 | Durra-Bicolor | 0 | 0 | Ethiopia | Eastern-Africa | 60.847 | 17.975 | -12.064 | 22.198 | -50.479 |
| SC1038 | PI576381 | Durra-Bicolor | 0 | 0 | Ethiopia | Eastern-Africa | 55.267 | 18.663 | -14.866 | 7.427 | -55.017 |
| SC1039 | PI576382 | Durra-Bicolor | 0 | 0 | Ethiopia | Eastern-Africa | 65.509 | 20.722 | -26.401 | 13.131 | -49.106 |
| SC1040 | PI595724 | Durra-Bicolor | 0 | 0 | Ethiopia | Eastern-Africa | 87.043 | 22.918 | -19.007 | -15.429 | -9.374 |
| SC1046 | PI576423 | Durra | 0 | 0 | Ethiopia | Eastern-Africa | 69.620 | 21.785 | -17.545 | 5.849 | -58.479 |
| SC1047 | PI656072 | Durra | 0 | 0 | Ethiopia | Eastern-Africa | 67.263 | 16.291 | -19.754 | 11.268 | -57.285 |
| SC1049 | PI576424 | Durra | 0 | 0 | Ethiopia | Eastern-Africa | 66.525 | 17.349 | -18.079 | 8.551 | -58.052 |
| SC1055 | PI595739 | Caudatum | 0 | 0 | Sudan | Northern-Africa | -58.826 | 59.479 | -21.638 | 2.008 | -11.340 |
| SC1056 | PI576387 | Caudatum | 0 | 0 | Sudan | Northern-Africa | -69.863 | 57.363 | -14.590 | -3.031 | 0.309 |
| SC1057 | PI595740 | Caudatum | 0 | 0 | Uganda | Eastern-Africa | -73.370 | 53.248 | -18.172 | -7.174 | 11.056 |
| SC1058 | NA | Unknown/Other | 0 | 0 | Cameroon | Middle-Africa | 74.821 | 3.028 | -9.081 | -24.573 | 28.182 |
| SC1063 | PI595741 | Guinea | 0 | 0 | Senegal | Western-Africa | -16.577 | -53.576 | 27.706 | -9.898 | 27.301 |
| SC1065 | PI597959 | Caudatum | 0 | 0 | Senegal | Western-Africa | -72.593 | 54.655 | -25.030 | -6.730 | 16.534 |
| SC1067 | PI576432 | Unknown/Other | 0 | 0 | Senegal | Western-Africa | -31.618 | 3.860 | -19.121 | 0.807 | 1.658 |
| SC1069 | PI576384 | Unknown/Other | 0 | 0 | Nigeria | Western-Africa | -9.922 | -48.461 | 62.439 | -9.831 | -22.701 |
| SC1070 | PI576385 | Unknown/Other | 0 | NA | Nigeria | Western-Africa | -6.510 | -53.911 | 40.290 | -6.598 | -51.413 |
| SC1072 | PI576433 | Kafir | 0 | 0 | Nigeria | Western-Africa | 13.756 | 3.019 | 45.724 | -17.182 | 5.694 |
| SC1074 | PI656073 | Kafir | 0 | 0 | Nigeria | Western-Africa | -19.614 | -57.244 | 17.545 | -13.630 | -10.650 |
| SC1076 | PI597960 | Caudatum | 0 | 0 | Nigeria | Western-Africa | -23.227 | -51.428 | -23.221 | -6.199 | -30.336 |
| SC1077 | PI597961 | Caudatum | 0 | 0 | Nigeria | Western-Africa | -60.945 | 59.921 | -23.040 | 2.903 | -5.661 |
| SC1079 | PI595714 | Caudatum | 0 | 0 | Sudan | Northern-Africa | -66.665 | 51.437 | -23.292 | -4.233 | 7.721 |
| SC1080 | PI576422 | Kafir | 0 | 0 | S.Africa | Southern-Africa | 1.628 | -15.267 | 128.113 | -10.113 | -9.034 |
| SC1082 | NA | Unknown/Other | 1 | 0 | Nigeria | Western-Africa | -8.639 | -39.448 | 31.823 | 12.687 | -5.105 |
| SC1083 | PI595729 | Guinea | 0 | 0 | Nigeria | Western-Africa | -16.173 | -50.166 | 10.262 | 18.365 | 8.500 |
| SC1084 | PI595730 | Guinea | 0 | 0 | Nigeria | Western-Africa | -17.864 | -45.881 | 28.742 | 6.616 | 34.693 |
| SC1085 | PI576401 | Durra | 0 | 0 | India | India | 87.262 | 25.513 | -21.831 | -16.932 | -7.277 |
| SC1088 | PI597948 | Durra | NA | NA | India | India | -45.839 | 35.320 | -10.891 | -6.395 | 21.759 |
| SC1089 | PI595705 | Durra | 0 | 1 | India | India | 75.074 | 28.326 | -23.898 | -15.585 | -6.536 |
| SC1097 | NA | Durra | 0 | 1 | India | India | 58.701 | 11.816 | 55.177 | -16.125 | -51.048 |

Table S4.1. continued

| SC1101 | PI595703 | Durra-Kafir | 0 | 1 | India | India | 88.971 | 24.851 | -21.767 | -18.193 | -11.865 |
| --- | --- | --- | --- | --- | --- | --- | --- | --- | --- | --- | --- |
| SC1103 | PI576434 | Bicolor | 0 | 0 | Nigeria | Western-Africa | -55.011 | 29.927 | -23.046 | -4.555 | 24.367 |
| SC1104 | PI576435 | Bicolor | 0 | 0 | Uganda | Eastern-Africa | -49.612 | 51.962 | 2.692 | -1.043 | -6.446 |
| SC1107 | NA | Bicolor | 0 | 0 | Japan | Asia | 33.082 | -9.537 | 40.481 | -7.290 | 22.563 |
| SC1108 | PI597949 | Guinea | 0 | 0 | India | India | 0.448 | -27.985 | 59.170 | -10.491 | 83.679 |
| SC1109 | PI576405 | Guinea-Bicolor | 0 | 1 | India | India | -13.085 | -20.965 | 73.162 | 5.689 | 42.575 |
| SC1111 | PI576409 | Caudatum | 0 | 0 | Sudan | Northern-Africa | -43.268 | 29.257 | -15.802 | 1.298 | -0.363 |
| SC1114 | NA | Caudatum | 0 | 0 | Sudan | Northern-Africa | -19.206 | 4.660 | 16.844 | -9.798 | -12.546 |
| SC1116 | PI576414 | Caudatum | 0 | 0 | Nigeria | Western-Africa | -31.272 | 12.812 | 47.046 | -12.978 | -13.860 |
| SC1117 | PI595710 | Caudatum | 0 | 0 | Nigeria | Western-Africa | -35.029 | -8.281 | -10.549 | -7.535 | -2.859 |
| SC1118 | PI595711 | Guinea | 0 | 1 | Nigeria | Western-Africa | -23.141 | -88.952 | -32.479 | 19.464 | 7.956 |
| SC1119 | NA | Guinea | 0 | 0 | Nigeria | Western-Africa | -37.228 | -77.052 | -27.014 | -15.055 | -1.150 |
| SC1120 | NA | Caudatum | 0 | 0 | Nigeria | Western-Africa | -19.910 | -0.871 | -19.848 | -2.842 | 0.558 |
| SC1123 | PI576417 | Guinea | 0 | 0 | Nigeria | Western-Africa | -24.733 | -80.579 | -7.424 | -9.704 | 28.171 |
| SC1124 | PI576418 | Guinea | 0 | 0 | Nigeria | Western-Africa | -39.314 | -113.355 | -48.181 | -13.018 | 3.197 |
| SC1125 | PI576419 | Guinea | 0 | 0 | Nigeria | Western-Africa | -38.564 | -109.877 | -43.297 | -12.867 | 1.631 |
| SC1133 | NA | Caudatum | 0 | 0 | Kenya | Eastern-Africa | -27.279 | 0.471 | 21.017 | -7.450 | 13.001 |
| SC1154 | PI595720 | Durra-Bicolor | NA | 0 | Ethiopia | Eastern-Africa | 63.500 | 18.969 | -27.727 | 9.307 | -53.157 |
| SC1155 | PI576425 | Durra-Bicolor | 0 | 0 | Ethiopia | Eastern-Africa | 62.672 | 21.370 | -31.645 | 13.474 | -51.715 |
| SC1156 | PI595721 | Durra-Bicolor | 0 | 0 | Ethiopia | Eastern-Africa | 61.804 | 15.199 | -22.066 | 12.093 | -45.269 |
| SC1157 | PI595722 | Durra-Bicolor | 0 | 0 | Ethiopia | Eastern-Africa | 62.981 | 15.425 | -19.920 | 20.042 | -46.087 |
| SC1158 | PI597957 | Durra | 0 | 0 | Ethiopia | Eastern-Africa | 58.842 | 7.334 | -15.718 | 8.913 | -50.063 |
| SC1159 | PI595723 | Durra | 0 | 0 | Ethiopia | Eastern-Africa | 60.119 | 18.915 | -25.291 | 6.448 | -41.627 |
| SC1160 | PI576427 | Durra-Bicolor | 0 | 0 | Ethiopia | Eastern-Africa | 22.715 | 0.979 | 20.999 | 99.194 | -15.066 |
| SC1166 | NA | Unknown/Other | 0 | 0 | Ethiopia | Eastern-Africa | 52.902 | 5.245 | 7.663 | 13.722 | -43.841 |
| SC1170 | NA | Durra | 0 | 0 | India | India | 54.408 | 5.274 | 37.568 | -2.589 | -42.593 |
| SC1172 | PI576436 | Caudatum | 0 | 0 | USA | Americas | -39.283 | 31.574 | 32.504 | -7.446 | 0.779 |
| SC1177 | PI595742 | Bicolor | 0 | 0 | Ethiopia | Eastern-Africa | 33.347 | -7.041 | 0.366 | 138.575 | 16.526 |
| SC1178 | NA | Unknown/Other | 0 | 0 | Nigeria | Western-Africa | -43.971 | 21.878 | -4.062 | 0.116 | -9.916 |
| SC1179 | NA | Unknown/Other | 0 | NA | Nigeria | Western-Africa | 77.628 | 15.992 | -2.234 | -28.523 | -7.788 |
| SC1184 | PI595719 | Durra-Bicolor | 0 | 0 | Ethiopia | Eastern-Africa | -3.137 | -4.756 | 48.825 | -0.116 | -39.352 |
| SC1186 | PI595731 | Kafir | 0 | 0 | Sudan | Northern-Africa | 47.916 | 3.717 | -14.691 | 0.008 | -12.137 |

Table S4.1. continued

| SC1193 | NA | Durra | 0 | 0 | Ethiopia | Eastern-Africa | 58.736 | 12.547 | -2.813 | -14.623 | -15.440 |
| --- | --- | --- | --- | --- | --- | --- | --- | --- | --- | --- | --- |
| SC1201 | PI595743 | Guinea-Caudatum | 0 | 0 | Unknown | Unknown | -2.708 | -9.780 | 54.422 | -4.022 | -28.635 |
| SC1203 | PI576437 | Bicolor | 0 | 0 | Brazil | Americas | -35.417 | 19.385 | 14.692 | 2.806 | -4.001 |
| SC1205 | PI597965 | Caudatum | 0 | 0 | Senegal | Western-Africa | -72.588 | 58.809 | -31.525 | -6.619 | 4.753 |
| SC1211 | PI595744 | Kafir-Caudatum | 0 | 0 | Guatemala | Americas | -20.467 | 4.618 | 22.542 | 0.067 | -0.506 |
| SC1212 | PI597966 | Caudatum | 0 | 0 | Venezuela | Americas | -73.517 | 61.711 | -9.200 | -8.928 | 7.516 |
| SC1214 | PI595745 | Guinea-Caudatum | 0 | 0 | BurkinaFaso | Western-Africa | -45.664 | 40.356 | -20.721 | -3.588 | 9.180 |
| SC1215 | PI656112 | Unknown/Other | 0 | 0 | Niger | Western-Africa | 11.826 | 6.649 | 48.042 | -1.225 | -40.615 |
| SC1216 | NA | Unknown/Other | 0 | 0 | Niger | Western-Africa | 7.142 | -13.296 | 61.472 | -6.580 | 54.878 |
| SC1218 | PI656074 | Unknown/Other | 0 | 0 | Unknown | Unknown | -18.783 | 11.585 | -8.922 | 7.523 | -13.003 |
| SC1222 | PI595713 | Caudatum | 0 | 0 | Japan | Asia | -35.611 | 20.548 | -18.461 | 7.372 | 12.997 |
| SC1229 | PI576415 | Guinea | 0 | 0 | Nigeria | Western-Africa | -35.303 | -68.968 | -29.678 | -11.176 | 9.151 |
| SC1237 | PI597955 | Caudatum-Kafir | 0 | 0 | Uganda | Eastern-Africa | -24.718 | -3.469 | 19.735 | 3.611 | 8.112 |
| SC1246 | PI595718 | Kafir-Caudatum | 0 | 0 | Chad | Middle-Africa | -33.455 | 32.152 | -12.751 | -0.658 | -3.881 |
| SC1251 | PI656075 | Caudatum | 0 | 0 | Sudan | Northern-Africa | -64.150 | 46.990 | -3.602 | -7.649 | 3.868 |
| SC1261 | PI595715 | Caudatum | 0 | 0 | Sudan | Northern-Africa | -68.154 | 58.143 | -23.825 | -6.093 | 1.526 |
| SC1262 | PI595716 | Caudatum | 0 | 0 | Sudan | Northern-Africa | -72.053 | 60.327 | -18.760 | -7.074 | 4.967 |
| SC1263 | PI595717 | Caudatum | 0 | 0 | Sudan | Northern-Africa | -54.399 | 54.469 | -20.539 | -2.978 | -4.203 |
| SC1271 | PI656076 | Caudatum | 0 | 0 | Ethiopia | Eastern-Africa | -80.129 | 65.158 | -21.644 | -6.858 | -6.347 |
| SC1277 | PI656113 | Durra | 0 | 0 | India | India | 77.466 | 25.626 | -15.575 | -7.884 | -32.648 |
| SC1287 | PI595746 | Caudatum | 0 | 1 | India | India | -73.022 | 62.921 | -11.846 | -5.118 | -5.295 |
| SC1293 | PI595747 | Caudatum | 0 | 0 | Senegal | Western-Africa | -53.291 | 11.761 | -19.695 | -8.063 | -8.686 |
| SC1300 | PI595732 | Caudatum | 0 | 0 | Ethiopia | Eastern-Africa | -80.286 | 67.486 | -23.191 | -6.396 | -2.190 |
| SC1302 | PI597962 | Caudatum | 0 | 0 | Ethiopia | Eastern-Africa | -82.916 | 67.909 | -24.492 | -6.937 | 5.525 |
| SC1305 | PI595733 | Caudatum | 0 | 0 | Ethiopia | Eastern-Africa | -66.684 | 60.403 | -8.334 | -3.545 | -11.553 |
| SC1307 | PI595734 | Caudatum | 0 | 1 | Ethiopia | Eastern-Africa | -64.088 | 65.436 | -18.371 | -1.782 | -16.480 |
| SC1313 | PI595735 | Caudatum | 0 | 0 | Ethiopia | Eastern-Africa | -69.537 | 57.734 | -22.970 | -4.500 | 9.177 |
| SC1314 | PI595736 | Caudatum | 0 | 0 | Ethiopia | Eastern-Africa | -69.468 | 53.228 | -23.920 | -3.694 | 13.997 |
| SC1316 | PI595737 | Caudatum | 0 | 0 | Ethiopia | Eastern-Africa | -80.780 | 65.246 | -22.495 | -6.258 | -2.433 |
| SC1317 | PI595738 | Caudatum | 0 | 0 | Ethiopia | Eastern-Africa | -76.472 | 61.986 | -23.955 | -4.292 | 3.951 |
| SC1318 | PI597963 | Caudatum | 0 | 0 | Ethiopia | Eastern-Africa | -63.166 | 65.084 | -29.341 | 1.163 | -6.218 |
| SC1319 | PI597964 | Caudatum | 0 | 0 | Ethiopia | Eastern-Africa | -75.346 | 66.018 | -24.512 | -2.933 | 1.603 |

Table S4.1. continued

| SC1320 | PI597967 | Caudatum | 0 | 0 | Ethiopia | Eastern-Africa | -72.228 | 56.787 | -18.903 | -6.525 | -0.059 |
| --- | --- | --- | --- | --- | --- | --- | --- | --- | --- | --- | --- |
| SC1321 | PI597968 | Caudatum | 0 | 0 | Sudan | Northern-Africa | -18.477 | 29.654 | -3.146 | -2.413 | -8.809 |
| SC1322 | PI597969 | Durra-Bicolor | 0 | 0 | Sudan | Northern-Africa | 21.211 | 5.976 | -9.403 | 9.751 | -11.043 |
| SC1325 | PI597970 | Caudatum | 0 | 0 | Sudan | Northern-Africa | -3.182 | 17.346 | -25.085 | 5.753 | 0.193 |
| SC1328 | PI597971 | Caudatum | 0 | 0 | Sudan | Northern-Africa | -35.327 | 30.740 | -5.128 | -1.892 | -6.295 |
| SC1329 | PI597972 | Durra-Caudatum | 0 | 0 | Sudan | Northern-Africa | 17.589 | 9.376 | -8.318 | 12.392 | -25.036 |
| SC1330 | PI597973 | Durra-Caudatum | 0 | 0 | Sudan | Northern-Africa | 13.897 | 0.365 | -25.287 | 1.271 | 7.964 |
| SC1332 | PI597974 | Guinea | 0 | 0 | Mali | Western-Africa | -21.300 | -66.657 | -21.289 | -11.693 | 13.947 |
| SC1333 | PI597975 | Guinea | 0 | 0 | Mali | Western-Africa | -38.043 | -112.422 | -42.188 | -11.566 | 0.164 |
| SC1337 | PI597976 | Guinea | 0 | 0 | Mali | Western-Africa | -24.988 | -78.518 | -27.167 | -9.218 | 11.170 |
| SC1338 | PI595748 | Guinea | 0 | 0 | Mali | Western-Africa | -24.465 | -73.982 | -19.792 | -12.684 | 5.855 |
| SC1339 | PI597977 | Guinea | 0 | 0 | Mali | Western-Africa | -28.427 | -74.316 | -21.317 | -8.608 | 5.138 |
| SC1341 | PI597978 | Guinea | 0 | 0 | Mali | Western-Africa | -26.797 | -77.978 | -23.370 | -8.884 | 9.635 |
| SC1342 | PI597979 | Guinea | 0 | 1 | Mali | Western-Africa | -24.795 | -76.807 | -21.321 | -10.348 | 11.742 |
| SC1345 | PI597980 | Guinea | 0 | 0 | Mali | Western-Africa | -70.616 | 55.570 | -26.191 | -5.842 | 9.764 |
| SC1351 | PI597981 | Caudatum | 0 | 0 | Sudan | Northern-Africa | -40.129 | 24.925 | -14.487 | -0.537 | 10.497 |
| SC1356 | PI597982 | Caudatum | 0 | 0 | Sudan | Northern-Africa | -42.108 | 23.853 | -7.484 | -2.156 | 14.504 |
| SC1416 | PI656077 | Unknown/Other | 0 | 0 | Niger | Western-Africa | -10.807 | 0.319 | 10.854 | -7.057 | 1.279 |
| SC1424 | PI656078 | Caudatum | 0 | 1 | Mali | Western-Africa | -12.687 | -39.351 | 18.614 | 12.330 | 24.913 |
| SC1426 | PI656079 | Bicolor | 0 | 1 | Mali | Western-Africa | -29.806 | -76.852 | -25.685 | -9.783 | 8.153 |
| SC1429 | PI656080 | Bicolor | 0 | 1 | Unknown | Unknown | -20.897 | -32.749 | 79.889 | -4.851 | 62.705 |
| SC1439 | PI656081 | Guinea | 0 | 1 | Gambia | Western-Africa | 0.804 | -48.222 | 6.994 | 32.005 | 8.598 |
| SC1440 | PI656115 | Guinea | 1 | 0 | Gambia | Western-Africa | -9.052 | -50.092 | 8.061 | 20.793 | 19.427 |
| SC1441 | NA | Caudatum | 0 | NA | Zambia | Eastern-Africa | -19.888 | -27.791 | 32.200 | -2.373 | 33.518 |
| SC1442 | NA | Guinea | 0 | 0 | Malawi | Eastern-Africa | -17.198 | -42.834 | 67.219 | -1.514 | 60.884 |
| SC1446 | NA | Kafir | 0 | 0 | Zambia | Eastern-Africa | -21.796 | -31.763 | 34.485 | -2.976 | 39.181 |
| SC1451 | PI656083 | Caudatum | 0 | 0 | Malawi | Eastern-Africa | -51.754 | 32.055 | -11.736 | -4.647 | 2.185 |
| SC1463 | PI656084 | Caudatum | NA | 0 | No.Kordofan,Sudan | Northern-Africa | -56.206 | 45.265 | -18.136 | -3.892 | 3.367 |
| SC1465 | PI656085 | Unknown/Other | 0 | 0 | No.Kordofan,Sudan | Northern-Africa | -60.132 | 55.746 | -13.431 | -0.968 | -8.744 |
| SC1471 | PI656086 | Unknown/Other | 0 | 0 | No.Kordofan,Sudan | Northern-Africa | 8.150 | 22.359 | -15.944 | 9.892 | -17.575 |
| SC1476 | PI656087 | Unknown/Other | 0 | 0 | No.Kordofan,Sudan | Northern-Africa | -34.985 | 16.492 | 43.136 | -13.621 | -5.892 |
| SC1484 | PI656088 | Unknown/Other | 0 | 0 | Somalia | Eastern-Africa | 81.057 | 18.545 | -23.757 | -8.714 | -23.034 |

Table S4.1. continued

| SC1489 | PI656089 | Durra | 0 | 0 | Somalia | Eastern-Africa | -61.336 | 64.649 | -3.530 | 1.044 | -22.602 |
| --- | --- | --- | --- | --- | --- | --- | --- | --- | --- | --- | --- |
| SC1494 | PI656090 | Unknown/Other | 0 | 0 | Sudan | Northern-Africa | -30.330 | 13.140 | -10.590 | 8.113 | -0.912 |
| SC1506 | PI656116 | Unknown/Other | 0 | 0 | Mali | Western-Africa | -25.931 | 1.861 | 41.959 | -7.212 | 8.062 |
| SRN39 | PI656027 | Breeding-line | 0 | 1 | Breeding-line | Breeding-line | -34.563 | 47.354 | -24.245 | -2.744 | -8.952 |
| SURENO | PI561472 | Breeding-line | 0 | 1 | Breeding-line | Breeding-line | -49.604 | 43.423 | 29.547 | -4.758 | -18.222 |
| SanChiSan | PI542718 | Breeding-line | 0 | 0 | Breeding-line | Breeding-line | 63.580 | 16.970 | -11.578 | -7.517 | -6.745 |
| Segaolane | PI656023 | Breeding-line | 0 | 0 | Breeding-line | Breeding-line | -4.789 | -10.536 | 122.752 | -9.887 | -14.194 |
| ShanQuiRed | PI656025 | Breeding-line | 0 | 0 | Breeding-line | Breeding-line | 56.410 | 15.637 | -6.930 | -5.594 | -3.628 |
| Soberano | PI656026 | Breeding-line | 0 | 0 | Breeding-line | Breeding-line | -72.110 | 67.260 | -15.753 | -1.933 | -15.642 |
| SpurFeter | PI655973 | Breeding-line | 0 | 0 | Breeding-line | Breeding-line | -24.954 | 38.136 | -19.178 | 3.894 | -21.098 |
| Tx2741 | PI655979 | Breeding-line | 0 | 0 | Breeding-line | Breeding-line | -34.445 | 11.072 | 6.723 | -4.068 | -38.855 |
| Tx2911 | PI607931 | Breeding-line | 0 | 0 | Breeding-line | Breeding-line | -29.609 | 24.618 | 31.761 | -0.806 | -23.410 |
